# Comparative Analyses of Base Compositions, DNA Sizes, and Dinucleotide Frequency Profiles in Archaeal and Bacterial Chromosomes and Plasmids

**DOI:** 10.1155/2012/342482

**Published:** 2012-03-26

**Authors:** Hiromi Nishida

**Affiliations:** Agricultural Bioinformatics Research Unit, Graduate School of Agricultural Sciences, University of Tokyo, Tokyo 113-8657, Japan

## Abstract

In the present paper, I compared guanine-cytosine (GC) contents, DNA sizes, and dinucleotide frequency profiles in 109 archaeal chromosomes, 59 archaeal plasmids, 1379 bacterial chromosomes, and 854 bacterial plasmids. In more than 80% of archaeal and bacterial plasmids, the GC content was lower than that of the host chromosome. Furthermore, most of the differences in GC content found between a plasmid and its host chromosome were less than 10%, and the GC content in plasmids and host chromosomes was highly correlated (Pearson's correlation coefficient *r* = 0.965 in bacteria and 0.917 in archaea). These results support the hypothesis that horizontal gene transfers have occurred frequently via plasmid distribution during evolution. GC content and chromosome size were more highly correlated in bacteria (*r* = 0.460) than in archaea (*r* = 0.195). Interestingly, there was a tendency for archaea with plasmids to have higher GC content in the chromosome and plasmid than those without plasmids. Thus, the dinucleotide frequency profile of the archaeal plasmids has a bias toward high GC content.

## 1. Introduction

DNA base composition, specifically guanine-cytosine (GC) content, is a bacterial taxonomic marker. For example, actinobacteria have high, whereas clostridia have low GC-containing genomes [1]. In addition, assessing the dinucleotide frequency profile, a genome signature, of a genomic DNA sequence is a powerful tool to compare different chromosomes and plasmids [2–6]. In bacterial chromosomes, GC content and DNA size are correlated [7–10]. In bacterial phages, plasmids, and inserted sequences, the GC contents are lower than those of their host chromosomes [11].

Replication of and transcription from plasmid DNA are controlled mainly by factors encoded by the chromosome of the host organism. Therefore, it is hypothesized that the GC content and genome signature of a plasmid are similar to those of the chromosome of the host organism. In addition, it is believed that horizontal gene transfers have occurred frequently via plasmid distribution during evolution [12]. For example, a cell-cell communication system may be distributed among the genus *Streptomyces* using horizontal gene transfer via plasmids [13].

Prokaryotes consist of 2 evolutionarily distinct groups: archaea and bacteria [14]. Comparative genomics in bacteria is very advanced, while the whole genome sequence data of archaea is currently limited. Due to recent developments in DNA sequence technology, more than 100 archaeal genome sequences have been elucidated. In this study, I compared GC contents, DNA sizes, and dinucleotide frequency profiles in archaeal and bacterial chromosomes and plasmids.

## 2. Materials and Methods

In this study, 109 archaeal chromosomes, 59 archaeal plasmids, 1379 bacterial chromosomes, and 854 bacterial plasmids were used from the database OligoWeb, searching oligonucleotide frequencies (http://insilico.ehu.es/oligoweb/). According to the annotation of the database OligoWeb, chromosomes and plasmids were distinguished. Pearson's correlation coefficient calculation, statistical tests, and drawing plots were performed using the software R (http://www.r-project.org/).

## 3. Results

**Figure 1:**
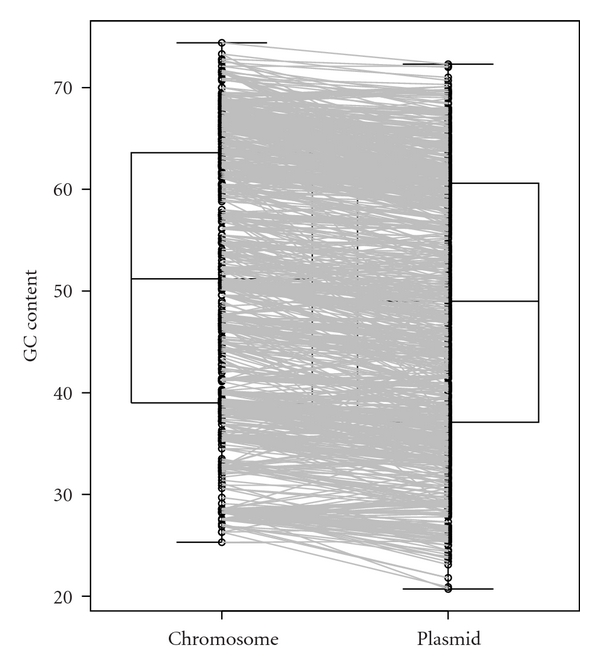
Boxplot of GC contents in bacterial plasmids and host chromosomes. Circles indicate the GC content (%) of each plasmid or chromosome, and lines link each plasmid to its host chromosome. The data set was shown in Supplementary Table S1 available online at doi:10.1155/2012/342482.

**Figure 2:**
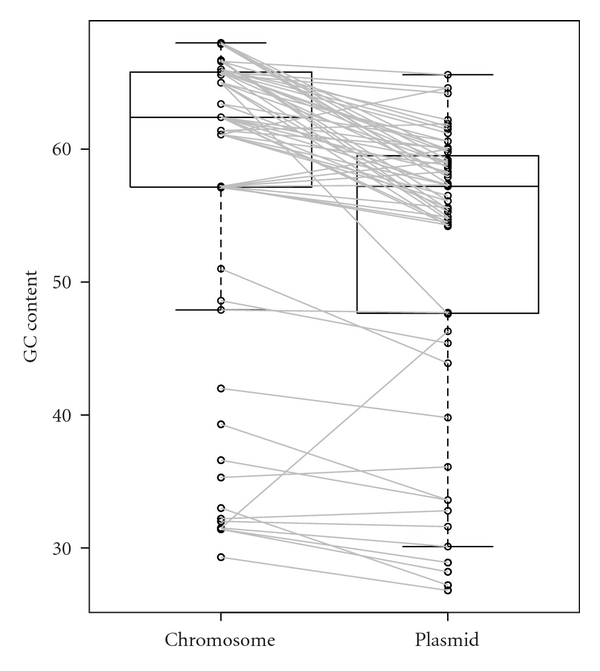
Boxplot of GC contents of archaeal plasmids and host chromosomes. Circles indicate the GC content (%) of each plasmid or chromosome, and lines link each plasmid to its host chromosome. The data set was shown in Supplementary Table S2.

**Figure 3:**
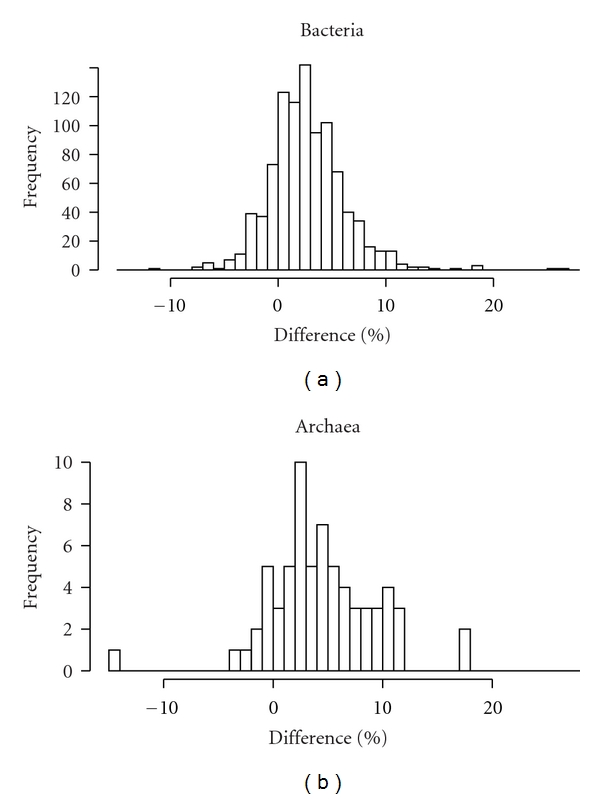
Histogram showing the difference between GC contents of plasmids and host chromosomes. Frequency means the number of pairs of chromosome and plasmid.

The 59 archaeal plasmids and 854 bacterial plasmids are distributed into 26 and 393 organisms, respectively. Some of the archaea and bacteria have 2 or 3 chromosomes. Therefore, in total, the 26 archaeal host organisms and 393 bacterial host organisms have 28 and 441 chromosomes, respectively. The GC contents of bacterial plasmids were found to be lower than those of the host chromosomes (Figure 1, Supplementary Table S1), which is consistent with a previous study [11]. In addition, the GC contents of archaeal plasmids were also lower than those of the host chromosomes (Figure 2, Supplementary Table S2). Furthermore, 777 (81.5%) of the 953 pairs of bacterial chromosome and plasmid, and 57 (85.1%) of the 67 pairs of archaeal chromosome and plasmid showed that the plasmid GC content is lower than that of its host chromosome (Figure 3). In addition, 746 (78.3%) of the 953 bacterial pairs and 47 (70.1%) of the 67 archaeal pairs showed less than 10% difference between GC content of the plasmid and its host chromosome (Figure 3).

**Figure 4:**
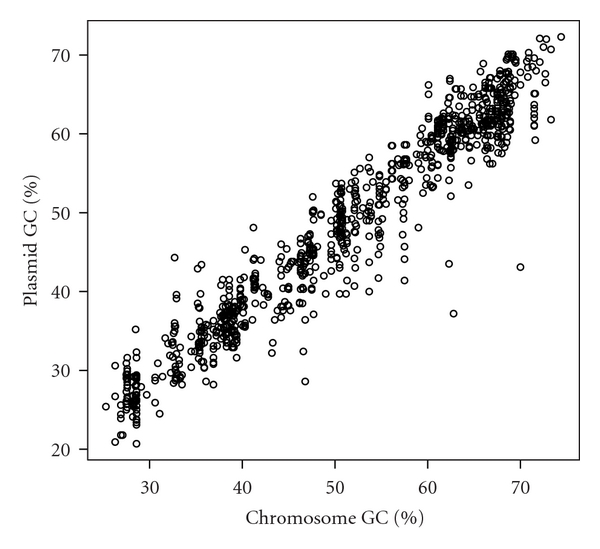
Scatter plot of GC contents of bacterial plasmids and host chromosomes. The Pearson's correlation coefficient is 0.965. The data set was shown in Supplementary Table S1.

**Figure 5:**
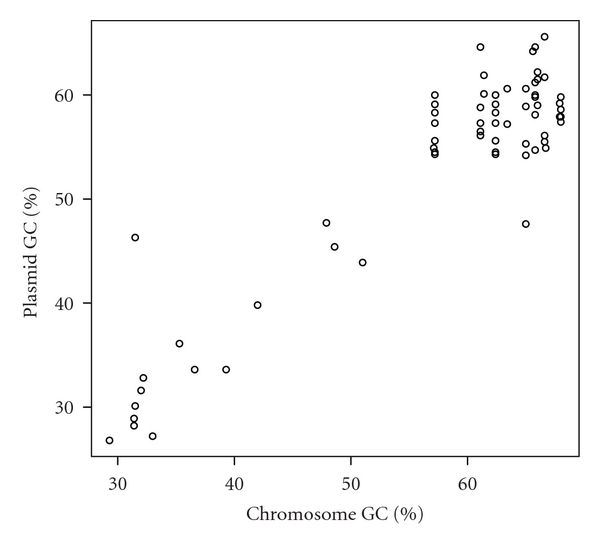
Scatter plot of GC contents of archaeal plasmids and host chromosomes. The Pearson's correlation coefficient is 0.917. The data set was shown in Supplementary Table S2.

**Figure 6:**
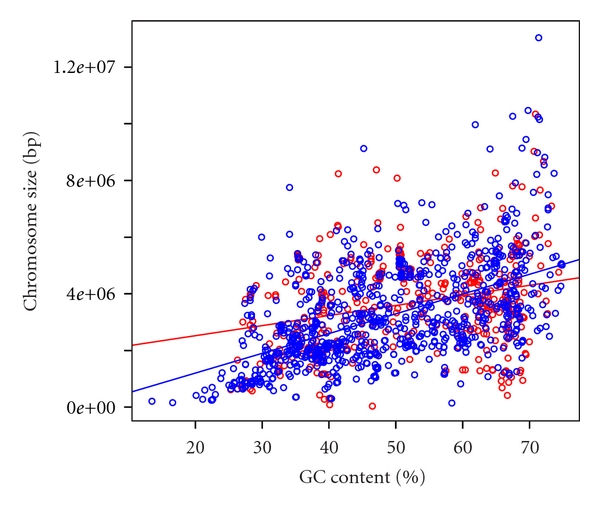
Scatter plot of GC contents and chromosome sizes in bacteria. Red and blue circles indicate chromosomes with and without plasmids, respectively. Red and blue lines indicate the regression lines. The data set was shown in Supplementary Table S3.

**Figure 7:**
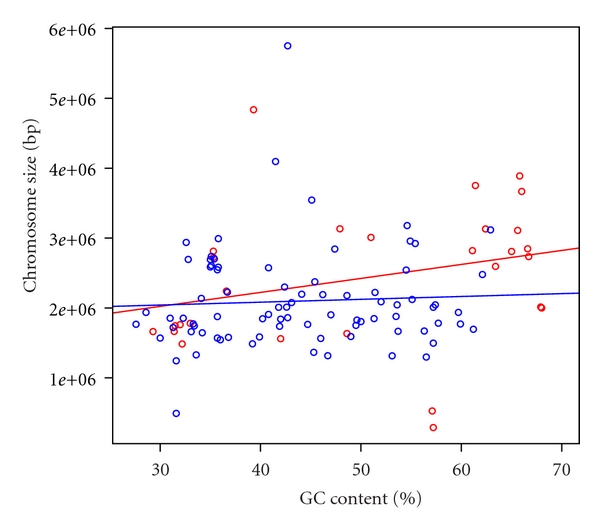
Scatter plot of GC contents and chromosome sizes in archaea. Red and blue circles indicate chromosomes with and without plasmids, respectively. Red and blue lines indicate the regression lines. The data set was shown in Supplementary Table S4.

**Figure 8:**
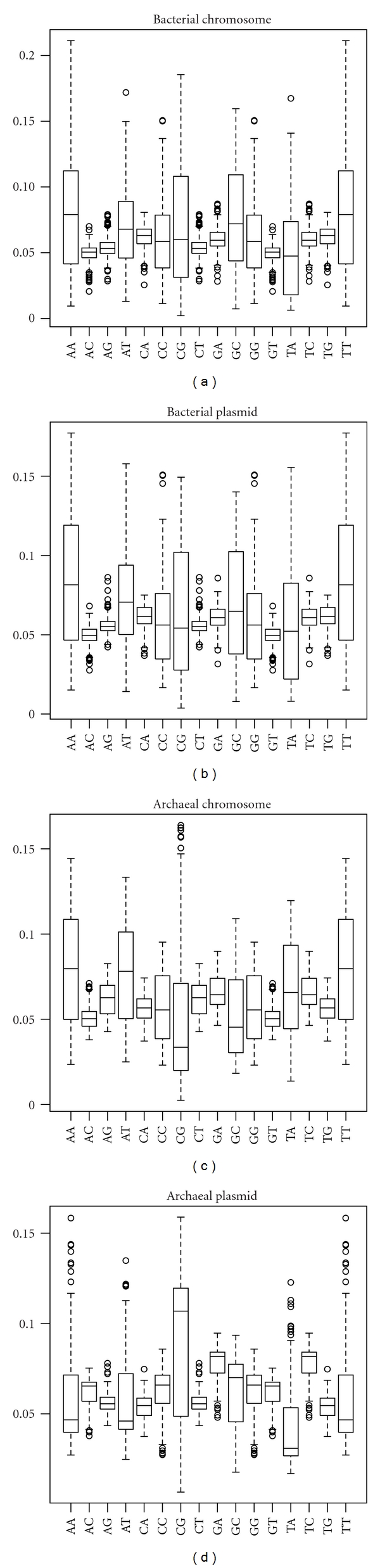
Boxplots of dinucleotide frequency profiles in chromosomes and plasmids of archaea and bacteria. Archaeal chromosomes, archaeal plasmids, bacterial chromosomes, and bacterial plasmids had frequency profiles of 109, 59, 1379, and 854, respectively.

 The GC contents in plasmids and the host chromosomes were highly correlated in both bacteria and archaea (Pearson's correlation coefficient *r* = 0.965 and *r* = 0.917, respectively; Figures 4 and 5, resp.). Furthermore, in terms of size, the GC content and chromosome size were more highly correlated in bacteria than archaea (Figures 6 and 7, Supplementary Tables S3 and S4). Pearson's correlation coefficients between GC content and chromosome size of archaea and bacteria were 0.195 and 0.460, respectively. In archaea, organisms with high GC content chromosome tend to have plasmid (Figures 2 and 7). Thus, the dinucleotide frequency profile of the archaeal plasmids has a bias toward high GC content (Figure 8).

## 4. Discussion

I hypothesize that GC content, a genomic signature, of a plasmid is related to host specificity and host range. Here, I showed that the GC content of a plasmid is lower than that of its host chromosome (Figures 1 and 2). However, in most cases, the difference in GC content between a plasmid and its host chromosome was less than 10% (Figure 3), strongly suggesting that host organisms cannot maintain and regulate plasmids with very different base compositions.

On the other hand, some organisms had a great difference in GC content between their chromosomes and plasmids. For example, in bacteria, *Frankia* symbiont of *Datisca glomerata* has the greatest difference (GC content of the chromosome is 70%; that of the plasmid pFSYMDG02 is 43.1%), and *Desulfovibrio magneticus* RS-1 has the second greatest difference (GC content of the chromosome is 62.8%; that of the plasmid pDMC2 is 37.2%) (Supplementary Table S1). I am so interested in the regulation system for these plasmids.

 In this analysis, there was a tendency for plasmid-containing archaea to have higher GC content in the host chromosome and plasmid than those without plasmids (Figures 2, 5, and 7). I have no idea why archaea with mid- and low-GC chromosome tend to lack plasmids. The GC content bias was not found in bacteria (Figures 1, 4, and 6). Thus, although the dinucleotide frequency profiles between the bacterial chromosomes and plasmids were similar, those between the archaeal chromosomes and plasmids were different (Figure 8).

GC content and chromosome size in bacteria are weakly correlated (*r* = 0.460), which is consistent with previous reports [7–10]. However, the GC content and chromosome size in archaea are less correlated (*r* = 0.195). Considering these results, the relationship between GC content and chromosome size may differ in archaea and bacteria. In order to understand the high GC content bias of archaeal plasmids and elucidate the relationship between GC content and chromosome size in archaea, more archaeal genome sequence data are needed.

## Supplementary Material

Supplementary Table S1: Pairs of chromosome and plasmid in bacteria.
Supplementary Table S2: Pairs of chromosome and plasmid in archaea.
Supplementary Table S3: Bacterial chromosomes compared in this analysis.
Supplementary Table S4: Archaeal chromosomes compared in this analysis.
