# Supplementary material for: Comparative Analyses of Base Compositions, DNA Sizes, and Dinucleotide Frequency Profiles in Archaeal and Bacterial Chromosomes and Plasmids
Source: Int J Evol Biol. 2012 Mar 26;2012:342482. doi: 10.1155/2012/342482 (PMC3321278; doi:10.1155/2012/342482)
Supplement: Supplementary file 1 — Supplementary Table S1: Pairs of chromosome and plasmid in bacteria. Supplementary Table S2: Pairs of chromosome and plasmid in archaea. Supplementary Table S3: Bacterial chromosomes compared in this analysis. Supplementary Table S4: Archaeal chromosomes compared in this analysis. [file 342482.f1.pdf]

Supplementary Table S1. Pairs of chromosome and plasmid in bacteria.

Organism	Chromosome GC %	Chromosome size (bp)	Plasmid	Plasmid GC %	Plasmid size (bp)
'Nostoc azollae' 0708	38.4	5354700	'Nostoc azollae' 0708 plasmid pAzo01	35.7	109570
'Nostoc azollae' 0708	38.4	5354700	'Nostoc azollae' 0708 plasmid pAzo02	33.5	21875
Acaryochloris marina MBIC11017	47.3	6503724	Acaryochloris marina MBIC11017 plasmid pREB1	47.3	374161
Acaryochloris marina MBIC11017	47.3	6503724	Acaryochloris marina MBIC11017 plasmid pREB2	45.3	356087
Acaryochloris marina MBIC11017	47.3	6503724	Acaryochloris marina MBIC11017 plasmid pREB3	45.2	273121
Acaryochloris marina MBIC11017	47.3	6503724	Acaryochloris marina MBIC11017 plasmid pREB4	45.9	226680
Acaryochloris marina MBIC11017	47.3	6503724	Acaryochloris marina MBIC11017 plasmid pREB5	44.7	177162
Acaryochloris marina MBIC11017	47.3	6503724	Acaryochloris marina MBIC11017 plasmid pREB6	47.1	172728
Acaryochloris marina MBIC11017	47.3	6503724	Acaryochloris marina MBIC11017 plasmid pREB7	45.6	155110
Acaryochloris marina MBIC11017	47.3	6503724	Acaryochloris marina MBIC11017 plasmid pREB8	45.4	120693
Acaryochloris marina MBIC11017	47.3	6503724	Acaryochloris marina MBIC11017 plasmid pREB9	42.5	2133

Achromobacter xylosoxidans A8	66	7013095	Achromobacter xylosoxidans A8 plasmid pA81	62.2	98156
Achromobacter xylosoxidans A8	66	7013095	Achromobacter xylosoxidans A8 plasmid pA82	61.3	247895
Acidiphilium cryptum JF-5	68	3389227	Acidiphilium cryptum JF-5 plasmid pACRY01	62.3	203589
Acidiphilium cryptum JF-5	68	3389227	Acidiphilium cryptum JF-5 plasmid pACRY02	61.9	187422
Acidiphilium cryptum JF-5	68	3389227	Acidiphilium cryptum JF-5 plasmid pACRY03	62.1	88953
Acidiphilium cryptum JF-5	68	3389227	Acidiphilium cryptum JF-5 plasmid pACRY04	59.6	37415
Acidiphilium cryptum JF-5	68	3389227	Acidiphilium cryptum JF-5 plasmid pACRY05	60.9	37155
Acidiphilium cryptum JF-5	68	3389227	Acidiphilium cryptum JF-5 plasmid pACRY06	61	8781
Acidiphilium cryptum JF-5	68	3389227	Acidiphilium cryptum JF-5 plasmid pACRY07	57.5	5629
Acidiphilium cryptum JF-5	68	3389227	Acidiphilium cryptum JF-5 plasmid pACRY08	58.7	4909
Acidiphilium multivorum AIU301	67.6	3749411	Acidiphilium multivorum AIU301	62.9	271573
Acidiphilium multivorum AIU301	67.6	3749411	Acidiphilium multivorum AIU301	61.9	65564
Acidiphilium multivorum AIU301	67.6	3749411	Acidiphilium multivorum AIU301	61.2	54248
Acidiphilium multivorum AIU301	67.6	3749411	Acidiphilium multivorum AIU301	60	40588
Acidiphilium multivorum AIU301	67.6	3749411	Acidiphilium multivorum AIU301	58.9	14328
Acidiphilium multivorum AIU301	67.6	3749411	Acidiphilium multivorum AIU301	59.6	12125

Acidiphilium multivorum AIU301	67.6	3749411	Acidiphilium multivorum AIU301	57.6	5178
Acidiphilium multivorum AIU301	67.6	3749411	Acidiphilium multivorum AIU301	60.9	1729
Acidobacterium sp. MP5ACTX9	60.5	4309153	Acidobacterium sp. MP5ACTX9 plasmid pACIX901	59.6	475658
Acidobacterium sp. MP5ACTX9	60.5	4309153	Acidobacterium sp. MP5ACTX9 plasmid pACIX902	59.4	300292
Acidobacterium sp. MP5ACTX9	60.5	4309153	Acidobacterium sp. MP5ACTX9 plasmid pACIX903	55.9	188167
Acidobacterium sp. MP5ACTX9	60.5	4309153	Acidobacterium sp. MP5ACTX9 plasmid pACIX904	53.3	115493
Acidobacterium sp. MP5ACTX9	60.5	4309153	Acidobacterium sp. MP5ACTX9 plasmid pACIX905	56.4	115221
Acidovorax sp. JS42	66.2	4448856	Acidovorax sp. JS42 plasmid pAOVO01	62.2	72689
Acidovorax sp. JS42	66.2	4448856	Acidovorax sp. JS42 plasmid pAOVO02	64.6	63609
Acinetobacter baumannii AB0057	39.2	4050513	Acinetobacter baumannii AB0057	34.4	8729
Acinetobacter baumannii ACICU	39	3904116	Acinetobacter baumannii ACICU plasmid pACICU1	37.8	28279
Acinetobacter baumannii ACICU	39	3904116	Acinetobacter baumannii ACICU plasmid pACICU2	33.4	64366
Acinetobacter baumannii ATCC 17978	38.9	3976747	Acinetobacter baumannii ATCC 17978 plasmid pAB1	36.2	13408

Acinetobacter baumannii ATCC 17978	38.9	3976747	Acinetobacter baumannii ATCC 17978 plasmid pAB2	35.2	11302
Acinetobacter baumannii AYE	39.4	3936291	Acinetobacter baumannii plasmid	34.5	5644
Acinetobacter baumannii AYE	39.4	3936291	Acinetobacter baumannii plasmid	34.4	9661
Acinetobacter baumannii AYE	39.4	3936291	Acinetobacter baumannii plasmid	37.5	94413
Acinetobacter baumannii AYE	39.4	3936291	Acinetobacter baumannii plasmid	37.6	2726
Acinetobacter baumannii SDF	39.2	3421954	Acinetobacter baumannii plasmid	34.5	6106
Acinetobacter baumannii SDF	39.2	3421954	Acinetobacter baumannii plasmid	34.9	25014
Acinetobacter baumannii SDF	39.2	3421954	Acinetobacter baumannii plasmid	34.4	24922
Actinobacillus pleuropneumoniae serovar 7 str. AP76	41.2	2331981	Actinobacillus pleuropneumoniae serovar 7 str. AP76 plasmid APP7 A	41.5	5685
Actinobacillus pleuropneumoniae serovar 7 str. AP76	41.2	2331981	Actinobacillus pleuropneumoniae serovar 7 str. AP76 plasmid ABB7 B	48.1	4236
Actinobacillus pleuropneumoniae serovar 7 str. AP76	41.2	2331981	Actinobacillus pleuropneumoniae serovar 7 str. AP76 plasmid APP7 C	41.7	3533
Agrobacterium radiobacter K84	60.3	4005130	Agrobacterium radiobacter K84 plasmid	57	388169
Agrobacterium radiobacter K84	60.3	4005130	Agrobacterium radiobacter K84 plasmid	59	184668
Agrobacterium radiobacter K84	60.3	4005130	Agrobacterium radiobacter K84 plasmid	53.5	44420

Agrobacterium radiobacter K84	59.8	2650913	Agrobacterium radiobacter K84 plasmid	57	388169
Agrobacterium radiobacter K84	59.8	2650913	Agrobacterium radiobacter K84 plasmid	59	184668
Agrobacterium radiobacter K84	59.8	2650913	Agrobacterium radiobacter K84 plasmid	53.5	44420
Agrobacterium sp. H13-3	58.8	2823930	Agrobacterium sp. H13-3 plasmid pAspH13-3a	57.4	601551
Agrobacterium vitis S4	57.7	3726375	Agrobacterium vitis S4 plasmid pTiS4	56.7	258824
Agrobacterium vitis S4	57.7	3726375	Agrobacterium vitis S4 plasmid pAtS4a	56.8	78730
Agrobacterium vitis S4	57.7	3726375	Agrobacterium vitis S4 plasmid pAtS4b	56.2	130435
Agrobacterium vitis S4	57.7	3726375	Agrobacterium vitis S4 plasmid pAtS4c	58.6	211620
Agrobacterium vitis S4	57.7	3726375	Agrobacterium vitis S4 plasmid pAtS4e	56.6	631775
Agrobacterium vitis S4 chromosome 2	57.5	1283187	Agrobacterium vitis S4 plasmid pTiS4	56.7	258824
Agrobacterium vitis S4 chromosome 2	57.5	1283187	Agrobacterium vitis S4 plasmid pAtS4a	56.8	78730
Agrobacterium vitis S4 chromosome 2	57.5	1283187	Agrobacterium vitis S4 plasmid pAtS4b	56.2	130435
Agrobacterium vitis S4 chromosome 2	57.5	1283187	Agrobacterium vitis S4 plasmid pAtS4c	58.6	211620
Agrobacterium vitis S4 chromosome 2	57.5	1283187	Agrobacterium vitis S4 plasmid pAtS4e	56.6	631775
Alicycliphilus denitrificans BC	68.3	4637013	Alicycliphilus denitrificans BC	58.3	119718
Alicycliphilus denitrificans BC	68.3	4637013	Alicycliphilus denitrificans BC	64.4	78982
Alicycliphilus denitrificans K601	68	4995263	Alicycliphilus denitrificans K601	62.2	75488

Alicyclobacillus acidocaldarius subsp. acidocaldarius DSM 446	62.3	3018755	Alicyclobacillus acidocaldarius subsp. acidocaldarius DSM 446 plasmid pAACI01	54.1	91726
Alicyclobacillus acidocaldarius subsp. acidocaldarius DSM 446	62.3	3018755	/Alicyclobacillus acidocaldarius subsp. acidocaldarius DSM 446 plasmid pAACI02	43.5	64508
Alicyclobacillus acidocaldarius subsp. acidocaldarius DSM 446	62.3	3018755	/Alicyclobacillus acidocaldarius subsp. acidocaldarius DSM 446 plasmid pAACI03	43.5	64508
Aliivibrio salmonicida LFI1238 chromosome 1	39.2	3325165	Aliivibrio salmonicida LFI1238 plasmid pVSAL320	37.3	30807
Aliivibrio salmonicida LFI1238 chromosome 1	39.2	3325165	Aliivibrio salmonicida LFI1238 plasmid pVSAL54	38.1	5360
Aliivibrio salmonicida LFI1238 chromosome 1	39.2	3325165	Aliivibrio salmonicida LFI1238 plasmid pVSAL43	35.6	4327
Aliivibrio salmonicida LFI1238 chromosome 2	38.2	1206461	Aliivibrio salmonicida LFI1238 plasmid pVSAL320	37.3	30807
Aliivibrio salmonicida LFI1238 chromosome 2	38.2	1206461	Aliivibrio salmonicida LFI1238 plasmid pVSAL54	38.1	5360
Aliivibrio salmonicida LFI1238 chromosome 2	38.2	1206461	Aliivibrio salmonicida LFI1238 plasmid pVSAL43	35.6	4327
Aliivibrio salmonicida LFI1238 chromosome	40.1	83540	Aliivibrio salmonicida LFI1238 plasmid pVSAL320	37.3	30807

Aliivibrio salmonicida LFI1238 chromosome	40.1	83540	Aliivibrio salmonicida LFI1238 plasmid pVSAL54	38.1	5360
Aliivibrio salmonicida LFI1238 chromosome	40.1	83540	Aliivibrio salmonicida LFI1238 plasmid pVSAL43	35.6	4327
Allochromatium vinosum DSM 180	64.4	3526903	Allochromatium vinosum DSM 180 plasmid pALVIN01	61.9	102242
Allochromatium vinosum DSM 180	64.4	3526903	Allochromatium vinosum DSM 180 plasmid pALVIN02	53.5	39929
Ammonifex degensii KC4	59.4	2129237	Ammonifex degensii KC4 plasmid pADEG01	60.7	27830
Amycolicicoccus subflavus DQS3-9A1	62.2	4738809	Amycolicicoccus subflavus DQS3-9A1 plasmid pAS9A-1	63.9	17897
Amycolicicoccus subflavus DQS3-9A1	62.2	4738809	Amycolicicoccus subflavus DQS3-9A1 plasmid pAS9A-2	61.9	106784
Anabaena variabilis ATCC 29413	46.5	37151	Anabaena variabilis ATCC 29413 plasmid A	40.5	366354
Anabaena variabilis ATCC 29413	46.5	37151	Anabaena variabilis ATCC 29413 plasmid B	38.5	35762
Anabaena variabilis ATCC 29413	46.5	37151	Anabaena variabilis ATCC 29413 plasmid C	42	300758
Anabaena variabilis ATCC 29413	41.4	6365727	Anabaena variabilis ATCC 29413 plasmid A	40.5	366354
Anabaena variabilis ATCC 29413	41.4	6365727	Anabaena variabilis ATCC 29413 plasmid B	38.5	35762
Anabaena variabilis ATCC 29413	41.4	6365727	Anabaena variabilis ATCC 29413 plasmid C	42	300758
Anaerocellum thermophilum DSM 6725	35.2	2919718	Anaerocellum thermophilum DSM 6725 plasmid pATHE01	38.5	8291

Anaerocellum thermophilum DSM 6725	35.2	2919718	Anaerocellum thermophilum DSM 6725 plasmid pATHE02	42.9	3653
Anaerococcus prevotii DSM 20548	36.1	1883067	Anaerococcus prevotii DSM 20548 plasmid pAPRE01	28.6	115566
Aquifex aeolicus	43.5	1551335	Aquifex aeolicus plasmid ece1	36.4	39456
Arthrobacter arilaitensis Re117	59.3	3859257	Arthrobacter arilaitensis Re117 plasmid pRE117-	58.8	50407
Arthrobacter arilaitensis Re117	59.3	3859257	Arthrobacter arilaitensis plasmid pRE117-2	55.5	8528
Arthrobacter aurescens TC1	62.3	4597686	Arthrobacter aurescens TC1 plasmid TC1	64.6	328237
Arthrobacter aurescens TC1	62.3	4597686	Arthrobacter aurescens TC1 plasmid TC2	61.3	300725
Arthrobacter chlorophenolicus A6	66.3	4395537	Arthrobacter chlorophenolicus A6 plasmid pACHL01	64.4	426858
Arthrobacter chlorophenolicus A6	66.3	4395537	Arthrobacter chlorophenolicus A6 plasmid pACHL02	61.3	158475
Arthrobacter phenanthrenivorans Sphe3	65.6	4250414	Arthrobacter phenanthrenivorans Sphe3 plasmid	62	190450
Arthrobacter phenanthrenivorans Sphe3	65.6	4250414	Arthrobacter phenanthrenivorans Sphe3 plasmid	62.4	94456
Arthrobacter sp. FB24	65.5	4698945	Arthrobacter sp. FB24 plasmid 1	65	159538
Arthrobacter sp. FB24	65.5	4698945	Arthrobacter sp. FB24 plasmid 2	63.3	115507
Arthrobacter sp. FB24	65.5	4698945	Arthrobacter sp. FB24 plasmid 3	64.7	96488

Aster yellows witches-broom phytoplasma AYWB	26.9	706569	Aster yellows witches-broom phytoplasma AYWB plasmid pAYWB-I	25.6	3972
Aster yellows witches-broom phytoplasma AYWB	26.9	706569	Aster yellows witches'-broom phytoplasma AYWB plasmid pAYWB-II	23.9	4009
Aster yellows witches-broom phytoplasma AYWB	26.9	706569	Aster yellows witches'-broom phytoplasma AYWB plasmid pAYWB-III	21.8	5104
Aster yellows witches-broom phytoplasma AYWB	26.9	706569	Aster yellows witches'-broom phytoplasma AYWB plasmid pAYWB-IV	24.4	4316
Asticcacaulis excentricus CB 48	59.2	2588221	Asticcacaulis excentricus CB 48	59.8	244260
Asticcacaulis excentricus CB 48	59.2	2588221	Asticcacaulis excentricus CB 48	57.3	160346
Asticcacaulis excentricus CB 48	60.4	1315949	Asticcacaulis excentricus CB 48	59.8	244260
Asticcacaulis excentricus CB 48	60.4	1315949	Asticcacaulis excentricus CB 48	57.3	160346
Azospirillum sp. B510	67.8	3311395	Azospirillum sp. B510 plasmid pAB510a	67.6	1455109
Azospirillum sp. B510	67.8	3311395	Azospirillum sp. B510 plasmid pAB510b	67.5	723779
Azospirillum sp. B510	67.8	3311395	Azospirillum sp. B510 plasmid pAB510c	67.4	681723
Azospirillum sp. B510	67.8	3311395	Azospirillum sp. B510 plasmid pAB510d	68	628837
Azospirillum sp. B510	67.8	3311395	Azospirillum sp. B510 plasmid pAB510e	67.5	537299

Azospirillum sp. B510	67.8	3311395	Azospirillum sp. B510 plasmid pAB510f	65.9	261596
Bacillus anthracis CI	35.4	5196054	Bacillus anthracis CI plasmid pCI-XO1	32.5	181907
Bacillus anthracis CI	35.4	5196054	Bacillus anthracis CI plasmid pCI-XO2	33.1	94469
Bacillus anthracis CI	35.4	5196054	Bacillus anthracis CI plasmid pBAslCI14	37.9	14219
Bacillus anthracis str. A0248	35.4	5227419	Bacillus anthracis str. A0248 plasmid pXO1	32.5	181677
Bacillus anthracis str. A0248	35.4	5227419	Bacillus anthracis str. A0248 plasmid pXO2	33	94830
Bacillus anthracis str. Ames 0581	35.4	5227419	Bacillus anthracis str. Ames 0581 plasmid	32.5	181677
Bacillus anthracis str. Ames 0581	35.4	5227419	Bacillus anthracis str. Ames 0581 plasmid	33	94830
Bacillus anthracis str. CDC 684	35.4	5230115	Bacillus anthracis str. CDC 684 plasmid pX01	32.5	181773
Bacillus anthracis str. CDC 684	35.4	5230115	Bacillus anthracis str. CDC 684 plasmid pX02	33.1	94875
Bacillus cereus 03BB102	35.4	5269628	Bacillus cereus 03BB102 plasmid	32.2	179680
Bacillus cereus AH187	35.6	5269030	Bacillus cereus AH187 plasmid pAH187 12	31.1	12481
Bacillus cereus AH187	35.6	5269030	Bacillus cereus AH187 plasmid pAH187 270	34.2	270082
Bacillus cereus AH187	35.6	5269030	Bacillus cereus AH187 plasmid pAH187 45	35.5	45173
Bacillus cereus AH187	35.6	5269030	Bacillus cereus AH187 plasmid pAH187 3	34.9	3091
Bacillus cereus AH820	35.4	5302683	Bacillus cereus AH820 plasmid pAH820 10	33.6	10915
Bacillus cereus AH820	35.4	5302683	Bacillus cereus AH820 plasmid pAH820 3	34.9	3091

Bacillus cereus AH820	35.4	5302683	Bacillus cereus AH820 plasmid pAH820_272	33.6	272145
Bacillus cereus ATCC 14579	35.3	5411809	Bacillus cereus ATCC14579 plasmid pBClin15	38	15274
Bacillus cereus G9842	35.3	5387334	Bacillus cereus G9842 plasmid pG9842_140	32.9	140001
Bacillus cereus G9842	35.3	5387334	Bacillus cereus G9842 plasmid pG9842_209	33.5	209488
Bacillus cereus Q1	35.6	5214195	Bacillus cereus Q1 plasmid pBc53	35.1	52766
Bacillus cereus subsp. cytotoxis NVH 391-98	35.9	4087024	Bacillus cereus subsp. cytotoxis NVH 391-98 plasmid pBC9801	30.3	7135
Bacillus megaterium QM B1551	38.3	5097129	Bacillus megaterium QM B1551 plasmid	34.8	5428
Bacillus megaterium QM B1551	38.3	5097129	Bacillus megaterium QM B1551 plasmid	34.5	9098
Bacillus megaterium QM B1551	38.3	5097129	Bacillus megaterium QM B1551 plasmid	35.5	26587
Bacillus megaterium QM B1551	38.3	5097129	Bacillus megaterium QM B1551 plasmid	36.5	53865
Bacillus megaterium QM B1551	38.3	5097129	Bacillus megaterium QM B1551 plasmid	33.9	66985
Bacillus megaterium QM B1551	38.3	5097129	Bacillus megaterium QM B1551 plasmid	33	99694
Bacillus megaterium QM B1551	38.3	5097129	Bacillus megaterium QM B1551 plasmid	33.5	164406
Bacillus pseudofirmus OF4	40.3	3858997	Bacillus pseudofirmus OF4 plasmid pBpOF4-	36	285222
Bacillus pseudofirmus OF4	40.3	3858997	Bacillus pseudofirmus OF4 plasmid pBpOF4-	35.5	105029
Bacillus thuringiensis BMB171	35.3	5330088	Bacillus thuringiensis BMB171 plasmid pBMB171	33.3	312963

Bacillus thuringiensis str. Al Hakam	35.4	5257091	Bacillus thuringiensis str. Al Hakam plasmid	36.2	55939
Bacillus weihenstephanensis KBAB4	35.6	5262775	Bacillus weihenstephanensis KBAB4 plasmid pBWB401	33.7	417054
Bacillus weihenstephanensis KBAB4	35.6	5262775	Bacillus weihenstephanensis KBAB4 plasmid pBWB402	33.3	75107
Bacillus weihenstephanensis KBAB4	35.6	5262775	Bacillus weihenstephanensis KBAB4 plasmid pBWB403	43.4	64977
Bacillus weihenstephanensis KBAB4	35.6	5262775	Bacillus weihenstephanensis KBAB4 plasmid pBWB404	35.4	52830
Bacteroides fragilis NCTC 9343	43.2	5205140	Bacteroides fragilis NCTC 9343 plasmid	32.2	36560
Bacteroides fragilis YCH46	43.3	5277274	Bacteroides fragilis YCH46 plasmid	33.5	33716
Bacteroides salanitronis DSM 18170	46.6	4242803	Bacteroides salanitronis DSM 18170 plasmid pBACSA01	32.4	40303
Bacteroides salanitronis DSM 18170	46.6	4242803	Bacteroides salanitronis DSM 18170 plasmid pBACSA02	43.2	19280
Bacteroides salanitronis DSM 18170	46.6	4242803	Bacteroides salanitronis DSM 18170 plasmid pBACSA03	40.3	6277
Bartonella grahamii as4aup	38.1	2341328	Bartonella grahamii as4aup plasmid pBGR3	36.4	28192
Bartonella tribocorum CIP 105476	38.9	2619061	Bartonella tribocorum CIP 105476 plasmidBtr	35	23343

Beijerinckia indica subsp. indica ATCC 9039	57.1	4170153	Beijerinckia indica subsp. indica ATCC 9039 plasmid pBIND01	56.2	181736
Beijerinckia indica subsp. indica ATCC 9039	57.1	4170153	Beijerinckia indica subsp. indica ATCC 9039 plasmid pBIND02	54.3	66727
Bifidobacterium longum DJO10A	60.1	2375792	Bifidobacterium longum DJO10A plasmid pDOJH10L	62.2	10073
Bifidobacterium longum DJO10A	60.1	2375792	Bifidobacterium longum DJO10A plasmid pDOJH10S	66.2	3661
Bifidobacterium longum subsp. infantis 157F	60.1	2400312	Bifidobacterium longum subsp. infantis 157F plasmid p157F-NC1	61.9	4895
Bifidobacterium longum subsp. infantis 157F	60.1	2400312	Bifidobacterium longum subsp. infantis 157F plasmid p157F-NC2	65	3624
Blattabacterium sp. (Periplaneta americana) str. BPLAN	28.2	636994	Blattabacterium sp. (Periplaneta americana) str. BPLAN plasmid pBPLAN	28.5	3448
Borrelia afzelii PKo	28.3	905394	Borrelia afzelii PKo plasmid cp30	29.2	30017
Borrelia afzelii PKo	28.3	905394	Borrelia afzelii PKo plasmid cp27	25.9	26533
Borrelia burgdorferi	28.6	910724	Borrelia burgdorferi plasmid cp32-1	29.4	30750
Borrelia burgdorferi	28.6	910724	Borrelia burgdorferi plasmid cp32-3	28.9	30223
Borrelia burgdorferi	28.6	910724	Borrelia burgdorferi plasmid cp32-4	29.3	30299
Borrelia burgdorferi	28.6	910724	Borrelia burgdorferi plasmid cp32-6	29.3	29838

Borrelia burgdorferi	28.6	910724	Borrelia burgdorferi plasmid cp32-7	29.1	30800
Borrelia burgdorferi	28.6	910724	Borrelia burgdorferi plasmid cp32-8	29.1	30885
Borrelia burgdorferi	28.6	910724	Borrelia burgdorferi plasmid cp32-9	29.3	30651
Borrelia burgdorferi	28.6	910724	Borrelia burgdorferi plasmid lp21	20.7	18753
Borrelia burgdorferi	28.6	910724	Borrelia burgdorferi plasmid lp56	27.3	52971
Borrelia burgdorferi	28.6	910724	Borrelia burgdorferi plasmid lp5	23.8	5228
Borrelia burgdorferi	28.6	910724	Borrelia burgdorferi plasmid lp17	23.1	16823
Borrelia burgdorferi	28.6	910724	Borrelia burgdorferi plasmid lp25	23.4	24177
Borrelia burgdorferi	28.6	910724	Borrelia burgdorferi plasmid lp28-1	32.3	26921
Borrelia burgdorferi	28.6	910724	Borrelia burgdorferi plasmid lp28-2	31.6	29766
Borrelia burgdorferi	28.6	910724	Borrelia burgdorferi plasmid lp28-3	25	28601
Borrelia burgdorferi	28.6	910724	Borrelia burgdorferi plasmid lp28-4	24.5	27323
Borrelia burgdorferi	28.6	910724	Borrelia burgdorferi plasmid lp36	26.9	36849
Borrelia burgdorferi	28.6	910724	Borrelia burgdorferi plasmid lp38	26.1	38829
Borrelia burgdorferi	28.6	910724	Borrelia burgdorferi plasmid lp54	28.2	53561
Borrelia burgdorferi	28.6	910724	Borrelia burgdorferi plasmid cp26	26.3	26498
Borrelia burgdorferi	28.6	910724	Borrelia burgdorferi plasmid cp9	23.7	9386

Borrelia burgdorferi ZS7	28.5	906707	Borrelia burgdorferi ZS7 plasmid ZS7 cp32-	28	48168
Borrelia burgdorferi ZS7	28.5	906707	Borrelia burgdorferi ZS7 plasmid ZS7_cp32-	29.3	30467
Borrelia burgdorferi ZS7	28.5	906707	Borrelia burgdorferi ZS7 plasmid ZS7_cp26	26.3	26514
Borrelia burgdorferi ZS7	28.5	906707	Borrelia burgdorferi ZS7 plasmid ZS7 cp32-	29.1	30330
Borrelia burgdorferi ZS7	28.5	906707	Borrelia burgdorferi ZS7 plasmid ZS7 cp32-	29.3	29806
Borrelia burgdorferi ZS7	28.5	906707	Borrelia burgdorferi ZS7 plasmid ZS7 cp32-	29	30964
Borrelia duttonii Ly	27.6	931674	Borrelia duttonii Ly plasmid pl11	25.3	11226
Borrelia duttonii Ly	27.6	931674	Borrelia duttonii Ly plasmid pl15	28.1	15049
Borrelia duttonii Ly	27.6	931674	Borrelia duttonii Ly plasmid pl28	28.9	28343
Borrelia duttonii Ly	27.6	931674	Borrelia duttonii Ly plasmid pl165	26.6	163884
Borrelia duttonii Ly	27.6	931674	Borrelia duttonii Ly plasmid pl35	31.6	35286
Borrelia duttonii Ly	27.6	931674	Borrelia duttonii Ly plasmid pl36	29.5	35831
Borrelia duttonii Ly	27.6	931674	Borrelia duttonii Ly plasmid pl40	29.9	40353
Borrelia duttonii Ly	27.6	931674	Borrelia duttonii Ly plasmid pl41	29.4	41259
Borrelia duttonii Ly	27.6	931674	Borrelia duttonii Ly plasmid pl42	30.2	42153
Borrelia duttonii Ly	27.6	931674	Borrelia duttonii Ly plasmid pl70	30.2	66407
Borrelia duttonii Ly	27.6	931674	Borrelia duttonii Ly plasmid pl23	26.4	23303

Borrelia duttonii Ly	27.6	931674	Borrelia duttonii Ly plasmid pl23b	29	23036
Borrelia duttonii Ly	27.6	931674	Borrelia duttonii Ly plasmid pl26	28.9	26917
Borrelia duttonii Ly	27.6	931674	Borrelia duttonii Ly plasmid pl31	27.8	31021
Borrelia duttonii Ly	27.6	931674	Borrelia duttonii Ly plasmid pl32	30.2	31663
Borrelia duttonii Ly	27.6	931674	Borrelia duttonii Ly plasmid pl27	28.5	27476
Borrelia recurrentis A1	27.5	930981	Borrelia recurrentis A1 plasmid pl124	26.1	123937
Borrelia recurrentis A1	27.5	930981	Borrelia recurrentis A1 plasmid pl23	25.7	22945
Borrelia recurrentis A1	27.5	930981	Borrelia recurrentis A1 plasmid pl33	29.5	33213
Borrelia recurrentis A1	27.5	930981	Borrelia recurrentis A1 plasmid pl35	27.3	35315
Borrelia recurrentis A1	27.5	930981	Borrelia recurrentis A1 plasmid pl37	29.4	36869
Borrelia recurrentis A1	27.5	930981	Borrelia recurrentis A1 plasmid pl53	29	52772
Borrelia recurrentis A1	27.5	930981	Borrelia recurrentis A1 plasmid pl6	30.9	6131
Brachyspira hyodysenteriae WA1	27.1	3000694	Brachyspira hyodysenteriae WA1 plasmid pBHWA1	21.8	35940
Bradyrhizobium sp. BTAi1	64.9	8264687	Bradyrhizobium sp. BTAi1 plasmid	60.7	228826
Buchnera aphidicola (Baizongia pistaciae)	25.3	615980	Buchnera aphidicola str. Bp (Baizongia pistaciae) plasmid pBBp1	25.4	2399
Buchnera sp. APS	26.3	640681	Buchnera sp. APS plasmid pTrp	30.6	7258

Buchnera sp. APS	26.3	640681	Buchnera sp. APS plasmid pLeu	26.7	7786
Burkholderia ambifaria MC40-6 chromosome 1	66.9	3443583	Burkholderia ambifaria MC40-6 plasmid pBMC401	61.2	301592
Burkholderia ambifaria MC40-6 chromosome 2	66.5	2769414	Burkholderia ambifaria MC40-6 plasmid pBMC401	61.2	301592
Burkholderia ambifaria MC40-6 chromosome 3	65.9	1127947	Burkholderia ambifaria MC40-6 plasmid pBMC401	61.2	301592
Burkholderia cenocepacia HI2424	66.8	3483902	Burkholderia cenocepacia HI2424	61.8	164857
Burkholderia cenocepacia HI2424	66.9	2998664	Burkholderia cenocepacia HI2424	61.8	164857
Burkholderia cenocepacia HI2424	67.3	1055417	Burkholderia cenocepacia HI2424	61.8	164857
Burkholderia cenocepacia J2315	66.7	3870082	Burkholderia cenocepacia J2315	62.8	92661
Burkholderia cenocepacia J2315	67.3	3217062	Burkholderia cenocepacia J2315	62.8	92661
Burkholderia cenocepacia J2315	66.9	875977	Burkholderia cenocepacia J2315	62.8	92661
Burkholderia cenocepacia MC0-3	66.7	3532883	Burkholderia cepacia AMMD plasmid 1	65.8	43581
Burkholderia cenocepacia MC0-3	66.6	3213911	Burkholderia cepacia AMMD plasmid 1	65.8	43581
Burkholderia cenocepacia MC0-3	66.2	1224595	Burkholderia cepacia AMMD plasmid 1	65.8	43581
Burkholderia cepacia AMMD chromosome 1	66.9	3556545	Burkholderia cepacia AMMD plasmid 1	65.8	43581
Burkholderia cepacia AMMD chromosome 2	66.8	2646969	Burkholderia cepacia AMMD plasmid 1	65.8	43581
Burkholderia cepacia AMMD chromosome 3	66.5	1281472	Burkholderia cepacia AMMD plasmid 1	65.8	43581

Burkholderia gladioli BSR3	67.5	4413616	Burkholderia gladioli BSR3 plasmid bgla 1p	63.3	276215
Burkholderia gladioli BSR3	67.5	4413616	Burkholderia gladioli BSR3 plasmid bgla_2p	62.8	129399
Burkholderia gladioli BSR3	67.5	4413616	Burkholderia gladioli BSR3 plasmid bgla_3p	59.6	128650
Burkholderia gladioli BSR3	67.5	4413616	Burkholderia gladioli BSR3 plasmid bgla 4p	62.4	403586
Burkholderia gladioli BSR3 chromosome 2	68.6	3700833	Burkholderia gladioli BSR3 plasmid bgla 1p	63.3	276215
Burkholderia gladioli BSR3 chromosome 2	68.6	3700833	Burkholderia gladioli BSR3 plasmid bgla 2p	62.8	129399
Burkholderia gladioli BSR3 chromosome 2	68.6	3700833	Burkholderia gladioli BSR3 plasmid bgla 3p	59.6	128650
Burkholderia gladioli BSR3 chromosome 2	68.6	3700833	Burkholderia gladioli BSR3 plasmid bgla_4p	62.4	403586
Burkholderia glumae BGR1	68.1	3906529	Burkholderia glumae BGR1 plasmid bglu 1p	60.6	133591
Burkholderia glumae BGR1	68.1	3906529	Burkholderia glumae BGR1 plasmid bglu 2p	63.2	141792
Burkholderia glumae BGR1	68.1	3906529	Burkholderia glumae BGR1 plasmid bglu 3p	62.7	141067
Burkholderia glumae BGR1	68.1	3906529	Burkholderia glumae BGR1 plasmid bglu 4p	62.7	134349
Burkholderia glumae BGR1 chromosome 2	68.8	2827355	Burkholderia glumae BGR1 plasmid bglu 1p	60.6	133591
Burkholderia glumae BGR1 chromosome 2	68.8	2827355	Burkholderia glumae BGR1 plasmid bglu 2p	63.2	141792
Burkholderia glumae BGR1 chromosome 2	68.8	2827355	Burkholderia glumae BGR1 plasmid bglu 3p	62.7	141067
Burkholderia glumae BGR1 chromosome 2	68.8	2827355	Burkholderia glumae BGR1 plasmid bglu 4p	62.7	134349
Burkholderia multivorans ATCC 17616 JGI	66.9	3448466	Burkholderia multivorans ATCC 17616 JGI plasmid	61.3	167422

Burkholderia multivorans ATCC 17616 JGI chromosome	67.1	2472928	Burkholderia multivorans ATCC 17616 JGI plasmid	61.3	167422
Burkholderia multivorans ATCC 17616 JGI chromosome	65.8	919806	Burkholderia multivorans ATCC 17616 JGI plasmid	61.3	167422
Burkholderia multivorans ATCC 17616 Tohoku	66.9	3448421	Burkholderia multivorans ATCC 17616 Tohoku plasmid	61.3	167422
Burkholderia multivorans ATCC 17616 Tohoku	67.1	2473162	Burkholderia multivorans ATCC 17616 Tohoku plasmid	61.3	167422
Burkholderia multivorans ATCC 17616 Tohoku	65.8	919805	Burkholderia multivorans ATCC 17616 Tohoku plasmid	61.3	167422
Burkholderia phymatum STM815	63	3479187	Burkholderia phymatum STM815 plasmid pBPHY01	61.9	1904893
Burkholderia phymatum STM815	63	3479187	Burkholderia phymatum STM815 plasmid pBPHY02	59.2	595108
Burkholderia phymatum STM815 chromosome 2	62.3	2697374	Burkholderia phymatum STM815 plasmid pBPHY01	61.9	1904893
Burkholderia phymatum STM815 chromosome 2	62.3	2697374	Burkholderia phymatum STM815 plasmid pBPHY02	59.2	595108
Burkholderia phytofirmans PsJN	62.6	4467537	Burkholderia phytofirmans PsJN	58.3	121122
Burkholderia phytofirmans PsJN	62.1	3625999	Burkholderia phytofirmans PsJN	58.3	121122
Burkholderia sp. CCGE1002	64.1	3518940	Burkholderia sp. CCGE1002 plasmid pBC201	59.2	489136

Burkholderia sp. CCGE1002 chromosome 2	63.2	2593966	Burkholderia sp. CCGE1002 plasmid pBC201	59.2	489136
Burkholderia sp. CCGE1002 chromosome 3	62.6	1282816	Burkholderia sp. CCGE1002 plasmid pBC201	59.2	489136
Burkholderia vietnamiensis G4	66.5	3652814	Burkholderia vietnamiensis G4	61.7	88096
Burkholderia vietnamiensis G4	66.5	3652814	Burkholderia vietnamiensis G4	61.8	265616
Burkholderia vietnamiensis G4	66.5	3652814	Burkholderia vietnamiensis G4	61	107231
Burkholderia vietnamiensis G4	66.5	3652814	Burkholderia vietnamiensis G4	59.8	226679
Burkholderia vietnamiensis G4	66.5	3652814	Burkholderia vietnamiensis G4	58.2	397868
Burkholderia vietnamiensis G4	66.8	2411759	Burkholderia vietnamiensis G4	61.7	88096
Burkholderia vietnamiensis G4	66.8	2411759	Burkholderia vietnamiensis G4	61.8	265616
Burkholderia vietnamiensis G4	66.8	2411759	Burkholderia vietnamiensis G4	61	107231
Burkholderia vietnamiensis G4	66.8	2411759	Burkholderia vietnamiensis G4	59.8	226679
Burkholderia vietnamiensis G4	66.8	2411759	Burkholderia vietnamiensis G4	58.2	397868
Burkholderia vietnamiensis G4	66.4	1241007	Burkholderia vietnamiensis G4	61.7	88096
Burkholderia vietnamiensis G4	66.4	1241007	Burkholderia vietnamiensis G4	61.8	265616
Burkholderia vietnamiensis G4	66.4	1241007	Burkholderia vietnamiensis G4	61	107231
Burkholderia vietnamiensis G4	66.4	1241007	Burkholderia vietnamiensis G4	59.8	226679

Burkholderia vietnamiensis G4	66.4	1241007	Burkholderia vietnamiensis G4	58.2	397868
Butyrivibrio proteoclasticus B316	40.2	3554804	Butyrivibrio proteoclasticus B316	39	361399
Butyrivibrio proteoclasticus B316	40.2	3554804	Butyrivibrio proteoclasticus B316	38.1	186325
Butyrivibrio proteoclasticus B316	40	302358	Butyrivibrio proteoclasticus B316	39	361399
Butyrivibrio proteoclasticus B316	40	302358	Butyrivibrio proteoclasticus B316	38.1	186325
Caldicellulosiruptor kristjanssonii 177R1B	36.1	2786473	Caldicellulosiruptor kristjanssonii 177R1B plasmid pCALKR01	35.8	15970
Calditerrivibrio nitroreducens DSM 19672	35.8	2157835	Calditerrivibrio nitroreducens DSM 19672 plasmid	31.1	58717
Campylobacter concisus 13826	39.4	2052007	Campylobacter concisus 13826 plasmid	31.6	30949
Campylobacter concisus 13826	39.4	2052007	Campylobacter concisus 13826 plasmid	33.5	16457
Campylobacter hominis ATCC BAA-381	31.7	1711273	Campylobacter hominis ATCC BAA-381 plasmid pCH4	34.1	3678
Campylobacter jejuni subsp. jejuni 81-176	30.6	1616554	Campylobacter jejuni subsp. jejuni 81-176 plasmid pVir	25.9	37473
Campylobacter jejuni subsp. jejuni 81-176	30.6	1616554	Campylobacter jejuni subsp. jejuni 81-176 plasmid pTet	29.1	45025
Campylobacter jejuni subsp. jejuni ICDCCJ07001	30.6	1664840	Campylobacter jejuni subsp. jejuni ICDCCJ07001 plasmid pTet	28.7	44084

Campylobacter lari RM2100	29.7	1525460	Campylobacter lari RM2100 megaplasmid pCL2100	26.9	46201
Candidatus Accumulibacter phosphatis clade IIA str. UW-1	64.1	5058518	Candidatus Accumulibacter phosphatis clade IIA str. UW-1 plasmid pAph01	61.6	167595
Candidatus Accumulibacter phosphatis clade IIA str. UW-1	64.1	5058518	Candidatus Accumulibacter phosphatis clade IIA str. UW-1 plasmid pAph02	60.7	42325
Candidatus Accumulibacter phosphatis clade IIA str. UW-1	64.1	5058518	Candidatus Accumulibacter phosphatis clade IIA str. UW-1 plasmid pAph03	59.3	37695
Candidatus Azobacteroides pseudotrichonymphae genomovar. CFP2	32.7	1114206	Candidatus Azobacteroides pseudotrichonymphae genomovar. CFP2 plasmid pCFPG1	36	37560
Candidatus Azobacteroides pseudotrichonymphae genomovar. CFP2	32.7	1114206	Candidatus Azobacteroides pseudotrichonymphae genomovar. CFP2 plasmid pCFPG2	35	37111
Candidatus Azobacteroides pseudotrichonymphae genomovar. CFP2	32.7	1114206	Candidatus Azobacteroides pseudotrichonymphae genomovar. CFP2 plasmid pCFPG3	35.6	31893
Candidatus Azobacteroides pseudotrichonymphae genomovar. CFP2	32.7	1114206	Candidatus Azobacteroides pseudotrichonymphae genomovar. CFP2 plasmid pCFPG4	44.3	4149

Candidatus Hamiltonella defensa 5AT (Acyrthosiphon pisum)	40.3	2110331	Candidatus Hamiltonella defensa 5AT (Acyrthosiphon pisum) plasmid	45.3	59032
Candidatus Riesia pediculicola USDA	28.5	574390	Candidatus Riesia pediculicola USDA plasmid pPAN	35.2	7737
Carnobacterium sp. 17-4	35.3	2635294	Carnobacterium sp. 17-4 plasmid pCAR50	31.5	50105
Caulobacter sp. K31	67.5	5477872	Caulobacter sp. K31 plasmid pCAUL01	67	233649
Caulobacter sp. K31	67.5	5477872	Caulobacter sp. K31 plasmid pCAUL02	64.3	177878
Chlamydia muridarum	40.3	1072950	Chlamydia muridarum plasmid pMoPn	35.7	7501
Chlamydophila caviae GPIC	39.2	1173390	Chlamydophila caviae GPIC plasmid	33.7	7966
Chlamydophila felis Fe C-56	39.4	1166239	Chlamydophila felis Fe C-56 plasmid pCfe1	33.9	7552
Chlamydophila psittaci 6BC	39.1	1171660	Chlamydophila psittaci 6BC plasmid p6BC	32.9	7553
Citrobacter koseri ATCC BAA-895	53.8	4720462	Citrobacter koseri ATCC BAA-895	55.2	9294
Citrobacter koseri ATCC BAA-895	53.8	4720462	Citrobacter koseri ATCC BAA-895	51.3	5601
Citrobacter rodentium ICC168	54.7	5346659	Citrobacter rodentium ICC168 plasmid	47.2	54449
Citrobacter rodentium ICC168	54.7	5346659	Citrobacter rodentium ICC168 plasmid	41.7	39265
Citrobacter rodentium ICC168	54.7	5346659	Citrobacter rodentium ICC168 plasmid	51	3910
Clavibacter michiganensis subsp. michiganensis NCPPB 382	72.7	3297891	Clavibacter michiganensis subsp. michiganensis NCPPB 382 plasmid pCM1	67.6	27357

Clavibacter michiganensis subsp. michiganensis NCPPB 382	72.7	3297891	Clavibacter michiganensis subsp. michiganensis NCPPB 382 plasmid pCM2	66.5	69989
Clostridium acetobutylicum	30.9	3940880	Clostridium acetobutylicum plasmid	30.9	192000
Clostridium botulinum A str. ATCC 3502	28.2	3886916	Clostridium botulinum A str. ATCC 3502 plasmid pBOT3502	26.8	16344
Clostridium botulinum A3 str. Loch Maree	28.3	3992906	Clostridium botulinum A3 str. Loch Maree plasmid pCLK	25.6	266785
Clostridium botulinum B str. Eklund 17B	27.5	3800327	Clostridium botulinum B str. Eklund 17B plasmid pCLL	25	47642
Clostridium botulinum B1 str. Okra	28.3	3958233	Clostridium botulinum B1 str. Okra plasmid	25.4	148780
Clostridium botulinum BKT015925	28.5	2773157	Clostridium botulinum BKT015925 plasmid p1BKT015925	26.5	203287
Clostridium botulinum BKT015925	28.5	2773157	Clostridium botulinum BKT015925 plasmid p2BKT015925	26.1	98732
Clostridium botulinum BKT015925	28.5	2773157	Clostridium botulinum BKT015925 plasmid p3BKT015925	26.8	80365
Clostridium botulinum BKT015925	28.5	2773157	Clostridium botulinum BKT015925 plasmid p4BKT015925	28.1	39648
Clostridium botulinum BKT015925	28.5	2773157	Clostridium botulinum BKT015925 plasmid p5BKT015925	26.3	12403
Clostridium botulinum Ba4 str. 657	28.2	3977794	Clostridium botulinum Ba4 str. 657 plasmid	25.6	270022

Clostridium botulinum Ba4 str. 657	28.2	3977794	Clostridium botulinum Ba4 str. 657 plasmid	24.1	9953
Clostridium botulinum F str. Langeland	28.3	3995387	Clostridium botulinum F str. Langeland plasmid pCLI	26.1	17531
Clostridium difficile 630	29.1	4290252	Clostridium difficile 630 plasmid pCD630	27.9	7881
Clostridium kluyveri DSM 555	32	3964618	Clostridium kluyveri DSM 555 plasmid	33.4	59182
Clostridium kluyveri NBRC 12016	32	3896121	Clostridium kluyveri NBRC 12016 plasmid pCKL1	33.4	59182
Clostridium perfringens	28.6	3031430	Clostridium perfringens plasmid pCP13	25.5	54310
Clostridium perfringens SM101	28.2	2897393	Clostridium perfringens SM101 plasmid 1	26.7	12397
Clostridium perfringens SM101	28.2	2897393	Clostridium perfringens SM101 plasmid 2	25.8	12206
Corynebacterium aurimucosum ATCC 700975	60.6	2790189	Corynebacterium aurimucosum plasmid pET44827	53.3	29037
Corynebacterium glutamicum R	54.1	3314179	Corynebacterium glutamicum R plasmid pCGR1	53.9	49120
Coxiella burnetii CbuK_Q154	42.7	2063100	Coxiella burnetii CbuK_Q154 plasmid pQpRS K Q154	39.7	39280
Coxiella burnetii Dugway 7E9-12	42.4	2158758	Coxiella burnetii Dugway 7E9-12	39.8	54179
Coxiella burnetii RSA 331	42.8	2016427	Coxiella burnetii RSA 331 plasmid QpH1	39.3	37317
Cronobacter turicensis	57.4	4384526	Cronobacter turicensis plasmid pCTU1	56.1	138339
Cronobacter turicensis	57.4	4384526	Cronobacter turicensis plasmid pCTU2	49.2	22448

Cronobacter turicensis	57.4	4384526	Cronobacter turicensis plasmid pCTU3	50	53842
Cupriavidus taiwanensis	67.5	3416911	Cupriavidus taiwanensis plasmid pRALTA	59.7	557200
Cupriavidus taiwanensis chromosome 2	67.9	2502411	Cupriavidus taiwanensis plasmid pRALTA	59.7	557200
Cyanothece sp. ATCC 51142 chromosome circular	37.9	4934271	Cyanothece sp. ATCC 51142 plasmid A	36.8	39620
Cyanothece sp. ATCC 51142 chromosome circular	37.9	4934271	Cyanothece sp. ATCC 51142 plasmid B	41.5	31856
Cyanothece sp. ATCC 51142 chromosome circular	37.9	4934271	Cyanothece sp. ATCC 51142 plasmid C	38.1	14685
Cyanothece sp. ATCC 51142 chromosome circular	37.9	4934271	Cyanothece sp. ATCC 51142 plasmid D	37	10244
Cyanothece sp. ATCC 51142 chromosome	38.6	429701	Cyanothece sp. ATCC 51142 plasmid A	36.8	39620
Cyanothece sp. ATCC 51142 chromosome	38.6	429701	Cyanothece sp. ATCC 51142 plasmid B	41.5	31856
Cyanothece sp. ATCC 51142 chromosome	38.6	429701	Cyanothece sp. ATCC 51142 plasmid C	38.1	14685
Cyanothece sp. ATCC 51142 chromosome	38.6	429701	Cyanothece sp. ATCC 51142 plasmid D	37	10244
Cyanothece sp. PCC 7424	38.6	5942652	Cyanothece sp. PCC 7424 plasmid pP742401	37.1	328635
Cyanothece sp. PCC 7424	38.6	5942652	Cyanothece sp. PCC 7424 plasmid pP742402	37.4	197705
Cyanothece sp. PCC 7424	38.6	5942652	Cyanothece sp. PCC 7424 plasmid pP742403	40.7	29239
Cyanothece sp. PCC 7424	38.6	5942652	Cyanothece sp. PCC 7424 plasmid pP742404	39.4	22636

Cyanothece sp. PCC 7424	38.6	5942652	Cyanothece sp. PCC 7424 plasmid pP742405	38.2	18083
Cyanothece sp. PCC 7424	38.6	5942652	Cyanothece sp. PCC 7424 plasmid pP742406	40.2	15219
Cyanothece sp. PCC 7425	50.8	5374574	Cyanothece sp. PCC 7425 plasmid pP742501	48.9	196837
Cyanothece sp. PCC 7425	50.8	5374574	Cyanothece sp. PCC 7425 plasmid pP742502	49.1	179973
Cyanothece sp. PCC 7425	50.8	5374574	Cyanothece sp. PCC 7425 plasmid pP742503	47.1	34726
Cyanothece sp. PCC 7822	40.2	6091620	Cyanothece sp. PCC 7822 plasmid Cy782203	39	291993
Cyanothece sp. PCC 7822	40.2	6091620	Cyanothece sp. PCC 7822 plasmid Cy782204	39	47550
Cyanothece sp. PCC 7822	40.2	6091620	Cyanothece sp. PCC 7822 plasmid Cy782205	41.8	43617
Cyanothece sp. PCC 8801	39.8	4679413	Cyanothece sp. PCC 8801 plasmid pP880101	38.9	50894
Cyanothece sp. PCC 8801	39.8	4679413	Cyanothece sp. PCC 8801 plasmid pP880102	40.2	40786
Cyanothece sp. PCC 8801	39.8	4679413	Cyanothece sp. PCC 8801 plasmid pP880103	40.4	16601
Cyanothece sp. PCC 8802	39.8	4669813	Cyanothece sp. PCC 8802 plasmid pP880201	39.5	75678
Cyanothece sp. PCC 8802	39.8	4669813	Cyanothece sp. PCC 8802 plasmid pP880202	41.5	23870
Cyanothece sp. PCC 8802	39.8	4669813	Cyanothece sp. PCC 8802 plasmid pP880203	39.3	22723
Cyanothece sp. PCC 8802	39.8	4669813	Cyanothece sp. PCC 8802 plasmid pP880204	38.3	11263
Deferribacter desulfuricans SSM1	31.1	2234389	Deferribacter desulfuricans SSM1 megaplasmid pDF308	24.5	308544
Deinococcus deserti VCD115	63.4	2819842	Deinococcus deserti VCD115 plasmid 1	60.7	324711

Deinococcus deserti VCD115	63.4	2819842	Deinococcus deserti VCD115 plasmid 2	63.5	314317
Deinococcus deserti VCD115	63.4	2819842	Deinococcus deserti VCD115 plasmid 3	61.4	396459
Deinococcus proteolyticus MRP	66.2	2147060	Deinococcus proteolyticus MRP	66.5	314518
Deinococcus proteolyticus MRP	66.2	2147060	Deinococcus proteolyticus MRP	61.4	195800
Deinococcus proteolyticus MRP	66.2	2147060	Deinococcus proteolyticus MRP	66.1	132270
Deinococcus proteolyticus MRP	66.2	2147060	Deinococcus proteolyticus MRP	59.2	97188
Deinococcus radiodurans R1	67	2648638	Deinococcus radiodurans plasmid	63.2	177466
Deinococcus radiodurans R1	67	2648638	Deinococcus radiodurans plasmid	56.2	45704
Deinococcus radiodurans R1 complete chromosome 2	66.7	412348	Deinococcus radiodurans plasmid MP1	63.2	177466
Deinococcus radiodurans R1 complete chromosome 2	66.7	412348	Deinococcus radiodurans plasmid CP1	56.2	45704
Desulfobacterium autotrophicum HRM2	48.8	5589073	Desulfobacterium autotrophicum HRM2 plasmid pHRM2a	42	68709
Desulfohalobium retbaense DSM 5692	57.5	2864304	Desulfohalobium retbaense DSM 5692 plasmid pDRET01	44.1	45263
Desulfotalea psychrophila LSv54	46.8	3523383	Desulfotalea psychrophila LSv54	43.6	121587
Desulfotalea psychrophila LSv54	46.8	3523383	Desulfotalea psychrophila LSv54	28.6	14664
Desulfovibrio magneticus RS-1	62.8	5248049	Desulfovibrio magneticus RS-1	58	58704

Desulfovibrio magneticus RS-1	62.8	5248049	Desulfovibrio magneticus RS-1	37.2	8867
Desulfovibrio vulgaris subsp. vulgaris DP4	63	3462887	Desulfovibrio vulgaris subsp. vulgaris DP4 plasmid pDVUL01	65.7	198504
Desulfovibrio vulgaris subsp. vulgaris str. Hildenborough	63.1	3570858	Desulfovibrio vulgaris subsp. vulgaris str. Hildenborough megaplasmid	65.7	202301
Dinoroseobacter shibae DFL 12	66	3789584	Dinoroseobacter shibae DFL 12 plasmid	60.1	190506
Dinoroseobacter shibae DFL 12	66	3789584	Dinoroseobacter shibae DFL 12 plasmid	65.2	152970
Dinoroseobacter shibae DFL 12	66	3789584	Dinoroseobacter shibae DFL 12 plasmid	60.5	126304
Dinoroseobacter shibae DFL 12	66	3789584	Dinoroseobacter shibae DFL 12 plasmid	61	86208
Dinoroseobacter shibae DFL 12	66	3789584	Dinoroseobacter shibae DFL 12 plasmid	68.9	72296
Edwardsiella tarda EIB202	59.7	3760463	Edwardsiella tarda EIB202 plasmid	57.3	43703
Enterobacter cloacae subsp. cloacae ATCC 13047	54.8	5314581	Enterobacter cloacae subsp. cloacae ATCC 13047 plasmid pECL A	52.5	199562
Enterobacter cloacae subsp. cloacae ATCC 13047	54.8	5314581	Enterobacter cloacae subsp. cloacae ATCC 13047 plasmid pECL B	46.8	84653
Enterobacter sakazakii ATCC BAA-894	56.8	4368373	Enterobacter sakazakii ATCC BAA-894 plasmid pESA2	51.6	31208
Enterobacter sakazakii ATCC BAA-894	56.8	4368373	Enterobacter sakazakii ATCC BAA-894 plasmid pESA3	56.8	131196
Enterobacter sp. 638	53	4518712	Enterobacter sp. 638 plasmid pENTE01	50.6	157749

Erwinia amylovora ATCC 49946	53.6	3805874	Erwinia amylovora ATCC 49946 plasmid 1	50.2	28243
Erwinia amylovora ATCC 49946	53.6	3805874	Erwinia amylovora ATCC 49946 plasmid 2	55.7	71487
Erwinia billingiae Eb661	55.2	5100167	Erwinia billingiae Eb661 plasmid pEB102	51.7	102323
Erwinia billingiae Eb661	55.2	5100167	Erwinia billingiae Eb661 plasmid pEB170	52.3	169778
Erwinia pyrifoliae Ep1/96	53.4	4026322	Erwinia pyrifoliae Ep1/96 plasmid pEP36	49.9	35909
Erwinia pyrifoliae Ep1/96	53.4	4026322	Erwinia pyrifoliae Ep1/96 plasmid pEP03	49	3070
Erwinia pyrifoliae Ep1/96	53.4	4026322	Erwinia pyrifoliae Ep1/96 plasmid pEP05	53.2	4955
Erwinia tasmaniensis	53.7	3883467	Erwinia tasmaniensis plasmid pET46	49	46159
Erwinia tasmaniensis	53.7	3883467	Erwinia tasmaniensis plasmid pET09	47	9299
Erwinia tasmaniensis	53.7	3883467	Erwinia tasmaniensis plasmid pET35	40	35494
Erwinia tasmaniensis	53.7	3883467	Erwinia tasmaniensis plasmid pET49	43.7	48751
Erwinia tasmaniensis	53.7	3883467	Erwinia tasmaniensis plasmid pET45	50.8	44694
Escherichia coli 0127:H6 E2348/69	50.6	4965553	Escherichia coli 0127:H6 E2348/69 plasmid pE2348-2	52.8	6147
Escherichia coli 0127:H6 E2348/69	50.6	4965553	Escherichia coli 0127:H6 E2348/69	48	97978
Escherichia coli E24377A	50.6	4979619	Escherichia coli E24377A plasmid	47.3	79237
Escherichia coli E24377A	50.6	4979619	Escherichia coli E24377A plasmid	51.6	34367
Escherichia coli E24377A	50.6	4979619	Escherichia coli E24377A plasmid	50.2	70609

Escherichia coli E24377A	50.6	4979619	Escherichia coli E24377A plasmid	52.6	6199
Escherichia coli E24377A	50.6	4979619	Escherichia coli E24377A plasmid	49.8	74224
Escherichia coli E24377A	50.6	4979619	Escherichia coli E24377A plasmid	49.6	5033
Escherichia coli O103:H2 str. 12009	50.7	5449314	Escherichia coli O103:H2 str. 12009	49.1	75546
Escherichia coli O111:H- str. 11128	50.6	5371077	Escherichia coli O111:H- str. 11128 plasmid pO111 1	47	204604
Escherichia coli O111:H- str. 11128	50.6	5371077	Escherichia coli O111:H- str. 11128 plasmid pO111 2	48.2	97897
Escherichia coli O111:H- str. 11128	50.6	5371077	Escherichia coli O111:H- str. 11128 plasmid pO111 3	50	77690
Escherichia coli O111:H- str. 11128	50.6	5371077	Escherichia coli O111:H- str. 11128 plasmid pO111 4	49.6	8140
Escherichia coli O111:H- str. 11128	50.6	5371077	Escherichia coli O111:H- str. 11128 plasmid pO111 5	50.2	6673
Escherichia coli O157:H7	50.5	5498450	Escherichia coli O157H7 plasmid	43.4	3306
Escherichia coli O157:H7	50.5	5498450	Escherichia coli O157:H7 plasmid	47.6	92721
Escherichia coli O157:H7 str. EC4115	50.5	5572075	Escherichia coli O157:H7 str. EC4115 plasmid pO157	47.9	94644
Escherichia coli O157:H7 str. EC4115	50.5	5572075	Escherichia coli O157:H7 str. EC4115 plasmid pEC4115	39.7	37452

Escherichia coli O157:H7 str. TW14359	50.5	5528136	Escherichia coli O157:H7 str. TW14359 plasmid pO157	47.9	94601
Escherichia coli O26:H11 str. 11368	50.7	5697240	Escherichia coli O26:H11 str. 11368	47.5	85167
Escherichia coli O26:H11 str. 11368	50.7	5697240	Escherichia coli O26:H11 str. 11368	52.5	63365
Escherichia coli O26:H11 str. 11368	50.7	5697240	Escherichia coli O26:H11 str. 11368	46.2	5686
Escherichia coli O55:H7 str. CB9615	50.5	5386352	Escherichia coli O55:H7 str. CB9615 plasmid	48.9	66001
Escherichia coli SE11	50.8	4887515	Escherichia coli SE11 plasmid pSE11-1	50.5	100021
Escherichia coli SE11	50.8	4887515	Escherichia coli SE11 plasmid pSE11-2	50.2	91158
Escherichia coli SE11	50.8	4887515	Escherichia coli SE11 plasmid pSE11-3	48.6	60555
Escherichia coli SE11	50.8	4887515	Escherichia coli SE11 plasmid pSE11-4	48	6929
Escherichia coli SE11	50.8	4887515	Escherichia coli SE11 plasmid pSE11-5	46.2	5366
Escherichia coli SE11	50.8	4887515	Escherichia coli SE11 plasmid pSE11-6	49.4	4082
Escherichia coli SMS-3-5	50.5	5068389	Escherichia coli SMS-3-5 plasmid pSMS35_8	46.8	8909
Escherichia coli SMS-3-5	50.5	5068389	Escherichia coli SMS-3-5 plasmid pSMS35_4	49.6	4074
Escherichia coli SMS-3-5	50.5	5068389	Escherichia coli SMS-3-5 plasmid pSMS35_3	43	3565
Escherichia coli SMS-3-5	50.5	5068389	Escherichia coli SMS-3-5 plasmid pSMS35_130	50.8	130440
Escherichia coli UTI89	50.6	5065741	Escherichia coli UTI89 plasmid pUTI89	51	114230

Eubacterium eligens ATCC 27750	37.7	2144190	Eubacterium eligens ATCC 27750 plasmid unnamed	36.8	626744
Eubacterium eligens ATCC 27750	37.7	2144190	Eubacterium eligens ATCC 27750 plasmid unnamed	40.8	60455
Exiguobacterium sibiricum 255-15	47.7	3034136	Exiguobacterium sibiricum 255-15	37.1	4885
Exiguobacterium sibiricum 255-15	47.7	3034136	Exiguobacterium sibiricum 255-15	41.4	1765
Finegoldia magna ATCC 29328	32.3	1797577	Finegoldia magna ATCC 29328 plasmid	29.7	189163
Francisella philomiragia subsp. philomiragia ATCC 25017	32.6	2045775	Francisella philomiragia subsp. philomiragia ATCC 25017 plasmid	28.4	3936
Frankia symbiont of Datisca glomerata	70	5323186	Frankia symbiont of Datisca glomerata plasmid pFSYMDG01	67.8	12355
Frankia symbiont of Datisca glomerata	70	5323186	Frankia symbiont of Datisca glomerata plasmid pFSYMDG02	43.1	5448
Gallibacterium anatis UMN179	39.9	2687335	Gallibacterium anatis UMN179 plasmid pUMN179	35.8	6804
Geobacillus kaustophilus HTA426	52.1	3544776	Geobacillus kaustophilus HTA426 plasmid pHTA426	44.2	47890
Geobacillus sp. WCH70	42.8	3464618	Geobacillus sp. WCH70 plasmid pWCH7001	39.6	33899
Geobacillus sp. WCH70	42.8	3464618	Geobacillus sp. WCH70 plasmid pWCH7002	39.6	10287
Geobacillus sp. Y4.1MC1	44	3840330	Geobacillus sp. Y4.1MC1 plasmid	43.8	71617

Geobacillus sp. Y412MC52	52.4	3628883	Geobacillus sp. Y412MC52 plasmid pGYMC5201	45.3	45057
Geobacillus sp. Y412MC61	52.4	3622844	Geobacillus sp. Y412MC61 plasmid pGYMC6101	45.3	45057
Geobacillus thermodenitrificans NG80-2	49	3550319	Geobacillus thermodenitrificans NG80-2 plasmid	39.7	57693
Geobacter lovleyi SZ	54.8	3917761	Geobacter lovleyi SZ plasmid pGLOV01	53	77113
Geobacter metallireducens GS-15	59.5	3997420	Geobacter metallireducens GS-15	52.5	13762
Glaciecola sp. 4H-3-7+YE-5	44.2	5052309	Glaciecola agarilytica 4H-3-7+YE-5 plasmid pGLAAG01	42	341282
Gluconacetobacter diazotrophicus PAl 5	66.4	3944163	Gluconacetobacter diazotrophicus PAl 5 plasmid pGDIPal5I	56.8	38818
Gluconacetobacter diazotrophicus PAl 5	66.4	3944163	Gluconacetobacter diazotrophicus PAl 5 plasmid pGDIPal5II	64	16610
Gluconacetobacter diazotrophicus PAl 5	66.4	3887492	Gluconacetobacter diazotrophicus PAl 5 plasmid pGDIA01	58.5	27455
Gluconobacter oxydans 621H	61.1	2702173	Gluconobacter oxydans 621H plasmid pGOX1	58.3	163186
Gluconobacter oxydans 621H	61.1	2702173	Gluconobacter oxydans 621H plasmid pGOX2	56.1	26568
Gluconobacter oxydans 621H	61.1	2702173	Gluconobacter oxydans 621H plasmid pGOX3	56	14547
Gluconobacter oxydans 621H	61.1	2702173	Gluconobacter oxydans 621H plasmid pGOX4	54.4	13223
Gluconobacter oxydans 621H	61.1	2702173	Gluconobacter oxydans 621H plasmid pGOX5	60	2687

Gordonia bronchialis DSM 43247	67.1	5208602	Gordonia bronchialis DSM 43247 plasmid	65.1	81410
Haemophilus somnus 129PT	37.2	2007700	Haemophilus somnus 129PT plasmid pHS129	35	5178
Haliscomenobacter hydrossis DSM 1100	47.1	8371686	Haliscomenobacter hydrossis DSM 1100 plasmid pHALHY01	46.2	164019
Haliscomenobacter hydrossis DSM 1100	47.1	8371686	Haliscomenobacter hydrossis DSM 1100 plasmid pHALHY02	46.4	143757
Haliscomenobacter hydrossis DSM 1100	47.1	8371686	Haliscomenobacter hydrossis DSM 1100 plasmid pHALHY02	47	92189
Helicobacter acinonychis str. Sheeba	38.2	1553927	Helicobacter acinonychis str. Sheeba	34.6	3661
Helicobacter pylori B8	38.8	1673997	Helicobacter pylori plasmid HPB8p	35.9	6032
Helicobacter pylori G27	38.9	1652982	Helicobacter pylori G27 plasmid pHPG27	34.9	10031
Helicobacter pylori HPAG1	39.1	1596366	Helicobacter pylori HPAG1 plasmid	36.4	9370
Helicobacter pylori P12	38.8	1673813	Helicobacter pylori P12 plasmid HPP12	35.1	10225
Helicobacter pylori PeCan4	38.9	1629557	Helicobacter pylori PeCan4 plasmid	32.9	8712
Herpetosiphon aurantiacus ATCC 23779	50.7	6346587	Herpetosiphon aurantiacus ATCC 23779 plasmid pHAU01	53.7	339639
Herpetosiphon aurantiacus ATCC 23779	50.7	6346587	Herpetosiphon aurantiacus ATCC 23779 plasmid pHAU02	53.1	99204
Hirschia baltica ATCC 49814	45.2	3455622	Hirschia baltica ATCC 49814 plasmid pHbal01	43.5	84492
Ilyobacter polytropus DSM 2926	34.5	2046464	Ilyobacter polytropus DSM 2926 plasmid	34.3	961624

Ilyobacter polytropus DSM 2926	34.5	2046464	Ilyobacter polytropus DSM 2926 plasmid	32.4	124226
Isosphaera pallida ATCC 43644	62.4	5472964	Isosphaera pallida ATCC 43644 plasmid	67	56340
Jannaschia sp. CCS1	62.3	4317977	Jannaschia sp. CCS1 plasmid1	57.8	86072
Ketogulonicigenium vulgare Y25	61.7	2776084	Ketogulonicigenium vulgare Y25 plasmid	61.4	268675
Ketogulonicigenium vulgare Y25	61.7	2776084	Ketogulonicigenium vulgare Y25 plasmid pYP12	62.6	243645
Kineococcus radiotolerans SRS30216	74.4	4761183	Kineococcus radiotolerans SRS30216 plasmid pKRAD02	72.3	12917
Klebsiella pneumoniae 342	57.3	5641239	Klebsiella pneumoniae 342 plasmid pKP91	51.1	91096
Klebsiella pneumoniae 342	57.3	5641239	Klebsiella pneumoniae 342 plasmid pKP187	47.2	187922
Klebsiella pneumoniae subsp. pneumoniae MGH 78578	57.5	5315120	Klebsiella pneumoniae subsp. pneumoniae MGH 78578 plasmid	51.7	175879
Klebsiella pneumoniae subsp. pneumoniae MGH 78578	57.5	5315120	Klebsiella pneumoniae subsp. pneumoniae MGH 78578 plasmid	53.4	107576
Klebsiella pneumoniae subsp. pneumoniae MGH 78578	57.5	5315120	Klebsiella pneumoniae subsp. pneumoniae MGH 78578 plasmid	53.8	88582
Klebsiella pneumoniae subsp. pneumoniae MGH 78578	57.5	5315120	Klebsiella pneumoniae subsp. pneumoniae MGH 78578 plasmid	41.4	4259
Klebsiella pneumoniae subsp. pneumoniae MGH 78578	57.5	5315120	Klebsiella pneumoniae subsp. pneumoniae MGH 78578 plasmid	45.7	3478

Lactobacillus acidophilus 30SC	38.1	2078001	Lactobacillus acidophilus 30SC plasmid pRKC30SC1	35.1	7197
Lactobacillus acidophilus 30SC	38.1	2078001	Lactobacillus acidophilus 30SC plasmid pRKC30SC2	36.6	12568
Lactobacillus brevis ATCC 367	46.2	2291220	Lactobacillus brevis ATCC 367 plasmid 1	38.6	13413
Lactobacillus brevis ATCC 367	46.2	2291220	Lactobacillus brevis ATCC 367 plasmid 2	38.5	35595
Lactobacillus buchneri NRRL B-30929	44.4	2506301	Lactobacillus buchneri NRRL B-30929 plasmid pLBUC01	38.1	52697
Lactobacillus buchneri NRRL B-30929	44.4	2506301	Lactobacillus buchneri NRRL B-30929 plasmid pLBUC02	40.4	18513
Lactobacillus buchneri NRRL B-30929	44.4	2506301	Lactobacillus buchneri NRRL B-30929 plasmid pLBUC03	37.6	10798
Lactobacillus casei ATCC 334	46.6	2895264	Lactobacillus casei ATCC 334 plasmid 1	42.2	29061
Lactobacillus delbrueckii subsp. bulgaricus ND02	49.6	2125753	Lactobacillus delbrueckii subsp. bulgaricus ND02	44.7	6223
Lactobacillus johnsonii FI9785	34.5	1755993	Lactobacillus johnsonii FI9785 plasmid p9785L	30.4	25652
Lactobacillus kefiranofaciens ZW3	37.7	2113023	Lactobacillus kefiranofaciens ZW3 plasmid pWW1	34	194769
Lactobacillus kefiranofaciens ZW3	37.7	2113023	Lactobacillus kefiranofaciens ZW3 plasmid pWW2	36	46296
Lactobacillus plantarum subsp. plantarum ST-III	44.6	3254376	Lactobacillus plantarum subsp. plantarum ST-III plasmid pST-III	38.7	53560

Lactobacillus rhamnosus Lc 705	46.7	2968598	Lactobacillus rhamnosus Lc 705	43.5	64508
Lactobacillus salivarius subsp. salivarius UCC118	32.9	1827111	Lactobacillus salivarius subsp. salivarius UCC118 plasmid	39.1	20417
Lactobacillus salivarius subsp. salivarius UCC118	32.9	1827111	Lactobacillus salivarius subsp. salivarius UCC118 plasmid	39.6	44013
Lactobacillus salivarius subsp. salivarius UCC118	32.9	1827111	Lactobacillus salivarius subsp. salivarius UCC118 plasmid	32.1	242436
Lactococcus lactis subsp. cremoris SK11	35.9	2438589	Lactococcus lactis subsp. cremoris SK11	34.4	14041
Lactococcus lactis subsp. cremoris SK11	35.9	2438589	Lactococcus lactis subsp. cremoris SK11	30.4	9554
Lactococcus lactis subsp. cremoris SK11	35.9	2438589	Lactococcus lactis subsp. cremoris SK11	35.4	74750
Lactococcus lactis subsp. cremoris SK11	35.9	2438589	Lactococcus lactis subsp. cremoris SK11	34.8	47208
Lactococcus lactis subsp. cremoris SK11	35.9	2438589	Lactococcus lactis subsp. cremoris SK11	33.5	14206
Lactococcus lactis subsp. lactis KF147	34.9	2598144	Lactococcus lactis subsp. lactis KF147 plasmid pKF147A	32.4	37510
Lawsonia intracellularis PHE/MN1-00	33.3	1457619	Lawsonia intracellularis PHE/MN1-00 plasmid 1	29	27048
Lawsonia intracellularis PHE/MN1-00	33.3	1457619	Lawsonia intracellularis PHE/MN1-00 plasmid 2	29.2	39794
Lawsonia intracellularis PHE/MN1-00	33.3	1457619	Lawsonia intracellularis PHE/MN1-00 plasmid 3	32.9	194553
Legionella pneumophila str. Lens	38.4	3345687	Legionella pneumophila str. Lens plasmid pLPL	38.4	59832
Legionella pneumophila str. Paris	38.4	3503610	Legionella pneumophila str. Paris plasmid pLPP	37.4	131885

Leptospira biflexa serovar Patoc strain Patoc 1 (Ames)	38.9	3603977	Leptospira biflexa serovar Patoc strain Patoc 1 (Ames) plasmid	37.5	74117
Leptospira biflexa serovar Patoc strain Patoc 1 (Ames)	39.3	277995	Leptospira biflexa serovar Patoc strain Patoc 1 (Ames) plasmid	37.5	74117
Leptospira biflexa serovar Patoc strain Patoc 1 (Paris)	38.9	3599677	Leptospira biflexa serovar Patoc strain Patoc 1 (Paris) plasmid	37.5	74116
Leptospira biflexa serovar Patoc strain Patoc 1 (Paris)	39.3	277655	Leptospira biflexa serovar Patoc strain Patoc 1 (Paris) plasmid	37.5	74116
Leuconostoc citreum KM20	39	1796284	Leuconostoc citreum KM20 plasmid pLCK1	37.4	38713
Leuconostoc citreum KM20	39	1796284	Leuconostoc citreum KM20 plasmid pLCK2	38	31463
Leuconostoc citreum KM20	39	1796284	Leuconostoc citreum KM20 plasmid pLCK3	33	17971
Leuconostoc citreum KM20	39	1796284	Leuconostoc citreum KM20 plasmid pLCK4	36.9	12183
Leuconostoc kimchii IMSNU11154	38	2002721	Leuconostoc kimchii IMSNU11154 plasmid LkipL4701	34.3	21055
Leuconostoc kimchii IMSNU11154	38	2002721	Leuconostoc kimchii IMSNU11154 plasmid LkipL4704	35.5	23275
Leuconostoc kimchii IMSNU11154	38	2002721	Leuconostoc kimchii IMSNU11154 plasmid LkipL4719	39.1	21924
Leuconostoc kimchii IMSNU11154	38	2002721	Leuconostoc kimchii IMSNU11154 plasmid LkipL4726	35.5	29616
Leuconostoc kimchii IMSNU11154	38	2002721	Leuconostoc kimchii IMSNU11154 plasmid LkipL48	37.1	3196

Leuconostoc mesenteroides subsp. mesenteroides ATCC 8293	37.7	2038396	Leuconostoc mesenteroides subsp. mesenteroides ATCC 8293 plasmid pLEUM1	35.4	37367
Listeria innocua Clip11262	37.4	3011208	Listeria innocua plasmid pLI100	35.5	81905
Lysinibacillus sphaericus C3-41	37.3	4639821	Lysinibacillus sphaericus C3-41	33.1	177642
Macrococcus caseolyticus JCSC5402	36.9	2102324	Macrococcus caseolyticus JCSC5402 plasmid pMCCL1	28.2	15667
Macrococcus caseolyticus JCSC5402	36.9	2102324	Macrococcus caseolyticus JCSC5402 plasmid pMCCL2	30.8	80545
Macrococcus caseolyticus JCSC5402	36.9	2102324	Macrococcus caseolyticus JCSC5402 plasmid pMCCL3	32.7	2131
Macrococcus caseolyticus JCSC5402	36.9	2102324	Macrococcus caseolyticus JCSC5402 plasmid pMCCL4	33.1	3417
Macrococcus caseolyticus JCSC5402	36.9	2102324	Macrococcus caseolyticus JCSC5402 plasmid pMCCL5	31.6	4398
Macrococcus caseolyticus JCSC5402	36.9	2102324	Macrococcus caseolyticus JCSC5402 plasmid pMCCL6	35.2	4226
Macrococcus caseolyticus JCSC5402	36.9	2102324	Macrococcus caseolyticus JCSC5402 plasmid pMCCL7	36.3	4061
Macrococcus caseolyticus JCSC5402	36.9	2102324	Macrococcus caseolyticus JCSC5402 plasmid pMCCL8	31	2968
Marinobacter aquaeolei VT8	57.3	4326849	Marinobacter aquaeolei VT8 plasmid	54.1	239623

Marinobacter aquaeolei VT8	57.3	4326849	Marinobacter aquaeolei VT8 plasmid	53.2	213290
Marivirga tractuosa DSM 4126	35.5	4511574	Marivirga tractuosa DSM 4126 plasmid	39.7	4916
Meiothermus silvanus DSM 9946	62.4	3249394	Meiothermus silvanus DSM 9946 plasmid pMESIL01	64.6	347854
Meiothermus silvanus DSM 9946	62.4	3249394	Meiothermus silvanus DSM 9946 plasmid pMESIL02	66.7	124421
Melissococcus plutonius ATCC 35311	31.4	1891014	Melissococcus plutonius ATCC 35311 plasmid pMP1	29.2	177718
Mesorhizobium ciceri biovar biserrulae WSM1271	62.7	6264489	Mesorhizobium ciceri biovar biserrulae WSM1271 plasmid pMESCI01	60.7	425539
Mesorhizobium loti MAFF303099	62.7	7036071	Mesorhizobium loti MAFF303099 plasmid pMLa	59.3	351911
Mesorhizobium loti MAFF303099	62.7	7036071	Mesorhizobium loti MAFF303099 plasmid pMLb	59.9	208315
Mesorhizobium sp. BNC1	61.1	4412446	Mesorhizobium sp. BNC1 plasmid 1	61.7	343931
Mesorhizobium sp. BNC1	61.1	4412446	Mesorhizobium sp. BNC1 plasmid 2	60.2	131247
Mesorhizobium sp. BNC1	61.1	4412446	Mesorhizobium sp. BNC1 plasmid 3	61.5	47561
Methylibium petroleiphilum PM1	69.2	4044195	Methylibium petroleiphilum PM1	66	599444
Methylobacterium chloromethanicum CM4	68.2	5777908	Methylobacterium chloromethanicum CM4 plasmid pMCHL01	66.3	380207

Methylobacterium chloromethanicum CM4	68.2	5777908	Methylobacterium chloromethanicum CM4 plasmid pMCHL02	63.9	22617
Methylobacterium extorquens AM1	68.7	5511322	Methylobacterium extorquens AM1 megaplasmid	67.6	1261460
Methylobacterium extorquens AM1	68.7	5511322	Methylobacterium extorquens AM1 plasmid p1META1	67.9	44195
Methylobacterium extorquens AM1	68.7	5511322	Methylobacterium extorquens AM1 plasmid p2META1	65.2	37858
Methylobacterium extorquens AM1	68.7	5511322	Methylobacterium extorquens AM1 plasmid p3META1	66.9	24943
Methylobacterium extorquens DM4	68.1	5943768	Methylobacterium extorquens DM4 plasmid p1METDI	65.3	141504
Methylobacterium extorquens DM4	68.1	5943768	Methylobacterium extorquens DM4 plasmid p2METDI	63.7	38579
Methylobacterium nodulans ORS 2060	68.9	7772460	Methylobacterium nodulans ORS 2060 plasmid pMNOD01	65.9	487734
Methylobacterium nodulans ORS 2060	68.9	7772460	Methylobacterium nodulans ORS 2060 plasmid pMNOD02	65.7	458070
Methylobacterium nodulans ORS 2060	68.9	7772460	Methylobacterium nodulans ORS 2060 plasmid pMNOD03	64.2	40463
Methylobacterium nodulans ORS 2060	68.9	7772460	Methylobacterium nodulans ORS 2060 plasmid pMNOD04	61.6	37542

Methylobacterium nodulans ORS 2060	68.9	7772460	Methylobacterium nodulans ORS 2060 plasmid pMNOD05	61.4	20286
Methylobacterium nodulans ORS 2060	68.9	7772460	Methylobacterium nodulans ORS 2060 plasmid pMNOD06	60.5	12638
Methylobacterium nodulans ORS 2060	68.9	7772460	Methylobacterium nodulans ORS 2060 plasmid pMNOD07	67.2	9829
Methylobacterium populi BJ001	69.4	5800441	Methylobacterium populi BJ001 plasmid	64.9	25164
Methylobacterium populi BJ001	69.4	5800441	Methylobacterium populi BJ001 plasmid	66.8	23392
Methylobacterium radiotolerans JCM 2831	71.5	6077833	Methylobacterium radiotolerans JCM 2831 plasmid pMRAD01	69.6	586164
Methylobacterium radiotolerans JCM 2831	71.5	6077833	Methylobacterium radiotolerans JCM 2831 plasmid pMRAD02	62.5	47003
Methylobacterium radiotolerans JCM 2831	71.5	6077833	Methylobacterium radiotolerans JCM 2831 plasmid pMRAD03	63.2	42985
Methylobacterium radiotolerans JCM 2831	71.5	6077833	Methylobacterium radiotolerans JCM 2831 plasmid pMRAD04	63.7	37743
Methylobacterium radiotolerans JCM 2831	71.5	6077833	Methylobacterium radiotolerans JCM 2831 plasmid pMRAD05	62	36410
Methylobacterium radiotolerans JCM 2831	71.5	6077833	Methylobacterium radiotolerans JCM 2831 plasmid pMRAD06	61	27836
Methylobacterium radiotolerans JCM 2831	71.5	6077833	Methylobacterium radiotolerans JCM 2831 plasmid pMRAD07	61.1	22114

Methylobacterium radiotolerans JCM 2831	71.5	6077833	Methylobacterium radiotolerans JCM 2831 plasmid pMRAD08	65.1	21022
Methylobacterium sp. 4-46	71.6	7659055	Methylobacterium sp. 4-46 plasmid pM44601	65.1	57951
Methylobacterium sp. 4-46	71.6	7659055	Methylobacterium sp. 4-46 plasmid pM44602	59.2	20019
Methylovorus sp. SIP3-4	54.9	2995511	Methylovorus sp. SIP3-4 plasmid pMsip01	45.7	76680
Methylovorus sp. SIP3-4	54.9	2995511	Methylovorus sp. SIP3-4 plasmid pMsip02	47.4	9816
Mycobacterium gilvum PYR-GCK	67.9	5619607	Mycobacterium gilvum PYR-GCK plasmid pMFLV01	65.1	321253
Mycobacterium gilvum PYR-GCK	67.9	5619607	Mycobacterium gilvum PYR-GCK plasmid pMFLV02	64.2	25309
Mycobacterium gilvum PYR-GCK	67.9	5619607	Mycobacterium gilvum PYR-GCK plasmid pMFLV03	65.2	16660
Mycobacterium marinum M	65.7	6636827	Mycobacterium marinum M plasmid	67.9	23317
Mycobacterium sp. KMS	68.4	5737227	Mycobacterium sp. KMS plasmid	65.4	302089
Mycobacterium sp. KMS	68.4	5737227	Mycobacterium sp. KMS plasmid	66.6	216763
Mycobacterium sp. MCS	68.5	5705448	Mycobacterium sp. MCS plasmid1	66.6	215075
Mycobacterium sp. Spyr1	67.9	5547747	Mycobacterium sp. Spyr1 plasmid	65.6	211864
Mycobacterium sp. Spyr1	67.9	5547747	Mycobacterium sp. Spyr1 plasmid	64.5	23681

Natranaerobius thermophilus JW-NM-WN-LF	36.3	3165557	Natranaerobius thermophilus JW-NM-WN-LF plasmid pNTHE01	34.2	17207
Natranaerobius thermophilus JW-NM-WN-LF	36.3	3165557	Natranaerobius thermophilus JW-NM-WN-LF plasmid pNTHE02	35.7	8689
Neisseria gonorrhoeae NCCP11945	52.4	2232025	Neisseria gonorrhoeae NCCP11945 plasmid pNGK	51.5	4153
Nitrobacter hamburgensis X14	61.7	4406967	Nitrobacter hamburgensis X14	60.4	294829
Nitrobacter hamburgensis X14	61.7	4406967	Nitrobacter hamburgensis X14	61.2	188318
Nitrobacter hamburgensis X14	61.7	4406967	Nitrobacter hamburgensis X14	61.7	121408
Nitrosococcus halophilus Nc4	51.6	4079427	Nitrosococcus halophilus Nc4 plasmid	53	65833
Nitrosococcus oceani ATCC 19707	50.3	3481691	Nitrosococcus oceani ATCC 19707 plasmid A	52.4	40420
Nitrosococcus watsoni C-113	50.1	3328570	Nitrosococcus watsoni C-113 plasmid	51.4	39105
Nitrosococcus watsoni C-113	50.1	3328570	Nitrosococcus watsoni C-113 plasmid	53.7	5611
Nitrosomonas eutropha C71	48.5	2661057	Nitrosomonas eutropha C71 plasmid1	49.8	65132
Nitrosomonas eutropha C71	48.5	2661057	Nitrosomonas eutropha C71 plasmid2	49.7	55635
Nitrosomonas sp. AL212	44.8	3180526	Nitrosomonas sp. AL212 plasmid	42.8	92707
Nitrosomonas sp. AL212	44.8	3180526	Nitrosomonas sp. AL212 plasmid	45.4	63790

Nitrosospira multiformis ATCC 25196 chromosome 1	53.9	3184243	Nitrosospira multiformis ATCC 25196 plasmid 1	49.5	18871
Nitrosospira multiformis ATCC 25196 chromosome 1	53.9	3184243	Nitrosospira multiformis ATCC 25196 plasmid 2	50	17036
Nitrosospira multiformis ATCC 25196 chromosome 1	53.9	3184243	Nitrosospira multiformis ATCC 25196 plasmid 3	49.6	14159
Nocardia farcinica IFM10152	70.8	6021225	Nocardia farcinica IFM10152 plasmid	67.2	184026
Nocardia farcinica IFM10152	70.8	6021225	Nocardia farcinica IFM10152 plasmid	68.4	87093
Nocardioides sp. JS614	71.7	4985871	Nocardioides sp. JS614 plasmid pNOCA01	68	307814
Nocardiopsis dassonvillei subsp. dassonvillei DSM	72.8	5767958	Nocardiopsis dassonvillei subsp. dassonvillei DSM	72	775354
Nostoc punctiforme PCC 73102	41.4	8234322	Nostoc punctiforme PCC 73102 plasmid	40.5	354564
Nostoc punctiforme PCC 73102	41.4	8234322	Nostoc punctiforme PCC 73102 plasmid	40.7	254918
Nostoc punctiforme PCC 73102	41.4	8234322	Nostoc punctiforme PCC 73102 plasmid	40.9	123028
Nostoc punctiforme PCC 73102	41.4	8234322	Nostoc punctiforme PCC 73102 plasmid	41.5	65940
Nostoc punctiforme PCC 73102	41.4	8234322	Nostoc punctiforme PCC 73102 plasmid	42.3	26419
Nostoc sp. PCC 7120	41.3	6413771	Nostoc sp. PCC 7120 plasmid pCC7120alpha	40.5	408101
Nostoc sp. PCC 7120	41.3	6413771	Nostoc sp. PCC 7120 plasmid pCC7120beta	40.2	186614
Nostoc sp. PCC 7120	41.3	6413771	Nostoc sp. PCC 7120 plasmid pCC7120delta	41.6	55414

Nostoc sp. PCC 7120	41.3	6413771	Nostoc sp. PCC 7120 plasmid	40.9	40340
Nostoc sp. PCC 7120	41.3	6413771	Nostoc sp. PCC 7120 plasmid	41	101965
Nostoc sp. PCC 7120	41.3	6413771	Nostoc sp. PCC 7120 plasmid pCC7120zeta	44.2	5584
Novosphingobium sp. PP1Y	63.7	3911486	Novosphingobium sp. PP1Y plasmid Mpl	62.3	1161602
Novosphingobium sp. PP1Y	63.7	3911486	Novosphingobium sp. PP1Y plasmid Lpl	60.7	192103
Novosphingobium sp. PP1Y	63.7	3911486	Novosphingobium sp. PP1Y plasmid Spl	60.1	48714
Oceanithermus profundus DSM 14977	70	2303940	Oceanithermus profundus DSM 14977 plasmid pOCEPR01	66.2	135351
Ochrobactrum anthropi ATCC 49188	56.1	2887297	Ochrobactrum anthropi ATCC 49188 plasmid pOANT01	56.2	170351
Ochrobactrum anthropi ATCC 49188	56.1	2887297	Ochrobactrum anthropi ATCC 49188 plasmid pOANT02	58.5	101491
Ochrobactrum anthropi ATCC 49188	56.1	2887297	Ochrobactrum anthropi ATCC 49188 plasmid pOANT03	54.3	93589
Ochrobactrum anthropi ATCC 49188	56.1	2887297	Ochrobactrum anthropi ATCC 49188 plasmid pOANT04	55.3	57138
Ochrobactrum anthropi ATCC 49188 chromosome 2	56.2	1895911	Ochrobactrum anthropi ATCC 49188 plasmid pOANT01	56.2	170351
Ochrobactrum anthropi ATCC 49188 chromosome 2	56.2	1895911	Ochrobactrum anthropi ATCC 49188 plasmid pOANT02	58.5	101491

Ochrobactrum anthropi ATCC 49188 chromosome 2	56.2	1895911	Ochrobactrum anthropi ATCC 49188 plasmid pOANT03	54.3	93589
Ochrobactrum anthropi ATCC 49188 chromosome 2	56.2	1895911	Ochrobactrum anthropi ATCC 49188 plasmid pOANT04	55.3	57138
Paenibacillus polymyxa SC2	45.2	5731816	Paenibacillus polymyxa SC2 plasmid pSC2	37.6	510115
Pantoea sp. At-9b	54.8	4368708	Pantoea sp. At-9b plasmid pPAT9B01	54.6	793953
Pantoea sp. At-9b	54.8	4368708	Pantoea sp. At-9b plasmid pPAT9B02	51	394054
Pantoea sp. At-9b	54.8	4368708	Pantoea sp. At-9b plasmid pPAT9B03	52	321080
Pantoea sp. At-9b	54.8	4368708	Pantoea sp. At-9b plasmid pPAT9B04	53.4	318111
Pantoea sp. At-9b	54.8	4368708	Pantoea sp. At-9b plasmid pPAT9B05	54.8	116877
Pantoea vagans C9-1	55.5	4024986	Pantoea vagans C9-1 plasmid pPag1	53	167983
Pantoea vagans C9-1	55.5	4024986	Pantoea vagans C9-1 plasmid pPag2	51.1	165693
Pantoea vagans C9-1	55.5	4024986	Pantoea vagans C9-1 plasmid pPag3	53.9	529676
Paracoccus denitrificans PD1222	66.7	2852282	Paracoccus denitrificans PD1222 plasmid 1	67.1	653815
Paracoccus denitrificans PD1222 chromosome 2	66.8	1730097	Paracoccus denitrificans PD1222 plasmid 1	67.1	653815
Pelobacter propionicus DSM 2379	59	4008000	Pelobacter propionicus DSM 2379 plasmid pPRO1	48.1	202397
Pelobacter propionicus DSM 2379	59	4008000	Pelobacter propionicus DSM 2379 plasmid pPRO2	56.3	30722

Persephonella marina EX-H1	37.2	1930284	Persephonella marina EX-H1 plasmid	34.4	53682
Phenylobacterium zucineum HLK1	71.3	3996255	Phenylobacterium zucineum HLK1 plasmid unnamed	68.5	382976
Photobacterium profundum SS9	42	4085304	Photobacterium profundum SS9 plasmid	44	80033
Photobacterium profundum SS9	41.2	2237943	Photobacterium profundum SS9 plasmid	44	80033
Photorhabdus asymbiotica	42.2	5064808	Photorhabdus asymbiotica plasmid	40.5	29330
Planctomyces limnophilus DSM 3776	53.7	5423075	Planctomyces limnophilus DSM 3776 plasmid pPLIM01	57	37010
Polaromonas naphthalenivorans CJ2	62.5	4410291	Polaromonas naphthalenivorans CJ2 plasmid pPNAP01	57.5	353291
Polaromonas naphthalenivorans CJ2	62.5	4410291	Polaromonas naphthalenivorans CJ2 plasmid pPNAP02	58	190172
Polaromonas naphthalenivorans CJ2	62.5	4410291	Polaromonas naphthalenivorans CJ2 plasmid pPNAP03	56.9	171866
Polaromonas naphthalenivorans CJ2	62.5	4410291	Polaromonas naphthalenivorans CJ2 plasmid pPNAP04	59	143747
Polaromonas naphthalenivorans CJ2	62.5	4410291	Polaromonas naphthalenivorans CJ2 plasmid pPNAP05	59.6	58808
Polaromonas naphthalenivorans CJ2	62.5	4410291	Polaromonas naphthalenivorans CJ2 plasmid pPNAP06	55.7	21611
Polaromonas naphthalenivorans CJ2	62.5	4410291	Polaromonas naphthalenivorans CJ2 plasmid pPNAP07	57.1	9898

Polaromonas naphthalenivorans CJ2	62.5	4410291	Polaromonas naphthalenivorans CJ2 plasmid pPNAP08	52.1	6459
Polaromonas sp. JS666	62.5	5200264	Polaromonas sp. JS666 plasmid 1	56.9	360405
Polaromonas sp. JS666	62.5	5200264	Polaromonas sp. JS666 plasmid 2	59.7	338007
Polymorphum gilvum SL003B-26A1	67.2	4649365	Polymorphum gilvum SL003B-26A1 plasmid pSL003B	61.6	69598
Prosthecochloris aestuarii DSM 271	50.1	2512923	Prosthecochloris aestuarii DSM 271 plasmid pPAES01	50.5	66772
Proteus mirabilis HI4320	38.9	4063606	Proteus mirabilis plasmid pHI4320	36.2	36289
Pseudomonas fluorescens SBW25	60.5	6722539	Pseudomonas fluorescens SBW25	53.2	425094
Pseudomonas syringae pv. phaseolicola 1448A	58	5928787	Pseudomonas syringae pv. phaseolicola 1448A large plasmid	54.1	131950
Pseudomonas syringae pv. phaseolicola 1448A	58	5928787	Pseudomonas syringae pv. phaseolicola 1448A small plasmid	56	51711
Pseudonocardia dioxanivorans CB1190	73.3	7096571	Pseudonocardia dioxanivorans CB1190 plasmid pPSED01	70.7	192355
Pseudonocardia dioxanivorans CB1190	73.3	7096571	Pseudonocardia dioxanivorans CB1190 plasmid pPSED03	61.8	15063
Psychrobacter cryohalolentis K5	42.3	3059876	Psychrobacter cryohalolentis K5 plasmid 1	38.3	41221
Psychrobacter sp. PRwf-1	44.9	2978976	Psychrobacter sp. PRwf-1 plasmid pRWF101	38.3	13956

Psychrobacter sp. PRwf-1	44.9	2978976	Psychrobacter sp. PRwf-1 plasmid pRWF102	40.4	2117
Pusillimonas sp. T7-7	56.9	3883605	Pusillimonas sp. T7-7 plasmid unnamed	56	41205
Rahnella sp. Y9602	52.3	4864217	Rahnella sp. Y9602 plasmid pRAHAQ01	52.1	616549
Rahnella sp. Y9602	52.3	4864217	Rahnella sp. Y9602 plasmid pRAHAQ02	48.3	133486
Ralstonia eutropha JMP134	64.7	3806533	Ralstonia eutropha JMP134 megaplasmid	60.6	634917
Ralstonia eutropha JMP134	64.7	3806533	Ralstonia eutropha JMP134 plasmid 1	64.7	87688
Ralstonia eutropha JMP134 chromosome 2	65	2726152	Ralstonia eutropha JMP134 megaplasmid	60.6	634917
Ralstonia eutropha JMP134 chromosome 2	65	2726152	Ralstonia eutropha JMP134 plasmid 1	64.7	87688
Ralstonia metallidurans CH34 chromosome 1	63.8	3928089	Ralstonia metallidurans CH34 plasmid 1	60.1	233720
Ralstonia metallidurans CH34 chromosome 1	63.8	3928089	Ralstonia metallidurans CH34 plasmid 2	60.5	171459
Ralstonia metallidurans CH34 chromosome 2	63.6	2580084	Ralstonia metallidurans CH34 plasmid 1	60.1	233720
Ralstonia metallidurans CH34 chromosome 2	63.6	2580084	Ralstonia metallidurans CH34 plasmid 2	60.5	171459
Ralstonia pickettii 12D	63.6	3647724	Ralstonia pickettii 12D plasmid pRp12D01	58.4	389779
Ralstonia pickettii 12D	63.6	3647724	Ralstonia pickettii 12D plasmid pRp12D02	61.6	273136
Ralstonia pickettii 12D	63.6	3647724	Ralstonia pickettii 12D plasmid pRp12D03	61.2	51398
Ralstonia pickettii 12D chromosome 2	64.5	1323321	Ralstonia pickettii 12D plasmid pRp12D01	58.4	389779
Ralstonia pickettii 12D chromosome 2	64.5	1323321	Ralstonia pickettii 12D plasmid pRp12D02	61.6	273136

Ralstonia pickettii 12D chromosome 2	64.5	1323321	Ralstonia pickettii 12D plasmid pRp12D03	61.2	51398
Ralstonia pickettii 12J	63.4	3942557	Ralstonia pickettii 12J plasmid pRPIC01	60.4	80934
Ralstonia pickettii 12J chromosome 2	64.5	1302238	Ralstonia pickettii 12J plasmid pRPIC01	60.4	80934
Rhizobium etli CFN 42	61.3	4381608	Rhizobium etli CFN 42 plasmid p42a	58	194229
Rhizobium etli CFN 42	61.3	4381608	Rhizobium etli CFN 42 plasmid p42b	61.8	184338
Rhizobium etli CFN 42	61.3	4381608	Rhizobium etli CFN 42 plasmid p42c	61.5	250948
Rhizobium etli CFN 42	61.3	4381608	Rhizobium etli CFN 42 plasmid p42e	61.7	505334
Rhizobium etli CFN 42	61.3	4381608	Rhizobium etli CFN 42 plasmid p42f	61.2	642517
Rhizobium etli CIAT 652	61.7	4513324	Rhizobium etli CIAT 652 plasmid pB	57.8	429111
Rhizobium etli CIAT 652	61.7	4513324	Rhizobium etli CIAT 652 plasmid pC	60.9	1091523
Rhizobium etli CIAT 652	61.7	4513324	Rhizobium etli CIAT 652 plasmid pA	62.2	414090
Rhizobium leguminosarum bv. trifolii WSM1325	61.1	4767043	Rhizobium leguminosarum bv. trifolii WSM1325	60.4	828924
Rhizobium leguminosarum bv. trifolii WSM1325	61.1	4767043	Rhizobium leguminosarum bv. trifolii WSM1325	60.8	660973
Rhizobium leguminosarum bv. trifolii WSM1325	61.1	4767043	Rhizobium leguminosarum bv. trifolii WSM1325	58.8	516088
Rhizobium leguminosarum bv. trifolii WSM1325	61.1	4767043	Rhizobium leguminosarum bv. trifolii WSM1325	60.6	350312

Rhizobium leguminosarum bv. trifolii WSM1325	61.1	4767043	Rhizobium leguminosarum bv. trifolii WSM1325	60.4	294782
Rhizobium leguminosarum bv. trifolii WSM2304	61.5	4537948	Rhizobium leguminosarum bv. trifolii WSM2304	60.4	1266105
Rhizobium leguminosarum bv. trifolii WSM2304	61.5	4537948	Rhizobium leguminosarum bv. trifolii WSM2304	62	501946
Rhizobium leguminosarum bv. trifolii WSM2304	61.5	4537948	Rhizobium leguminosarum bv. trifolii WSM2304	57.9	308747
Rhizobium leguminosarum bv. trifolii WSM2304	61.5	4537948	Rhizobium leguminosarum bv. trifolii WSM2304	61.4	257956
Rhizobium leguminosarum bv. viciae 3841	61.1	5057142	Rhizobium leguminosarum bv. viciae 3841 plasmid	58.7	147463
Rhizobium leguminosarum bv. viciae 3841	61.1	5057142	Rhizobium leguminosarum bv. viciae 3841 plasmid	61	352782
Rhizobium leguminosarum bv. viciae 3841	61.1	5057142	Rhizobium leguminosarum bv. viciae 3841 plasmid	59.6	488135
Rhizobium leguminosarum bv. viciae 3841	61.1	5057142	Rhizobium leguminosarum bv. viciae 3841 plasmid	61	684202
Rhizobium sp. NGR234	63	3925702	Rhizobium sp. NGR234 plasmid pNGR234a	58.5	536165
Rhizobium sp. NGR234	63	3925702	Rhizobium sp. NGR234 plasmid pNGR234b	62.3	2430033
Rhodobacter capsulatus SB1003	66.6	3738958	Rhodobacter capsulatus SB1003 plasmid	66.5	132962
Rhodobacter sphaeroides 2.4.1	69	3188609	Rhodobacter sphaeroides 2.4.1	70.1	114178

Rhodobacter sphaeroides 2.4.1	69	3188609	Rhodobacter sphaeroides 2.4.1	63.8	105284
Rhodobacter sphaeroides 2.4.1	69	3188609	Rhodobacter sphaeroides 2.4.1	63.8	100828
Rhodobacter sphaeroides 2.4.1	69	943016	Rhodobacter sphaeroides 2.4.1	70.1	114178
Rhodobacter sphaeroides 2.4.1	69	943016	Rhodobacter sphaeroides 2.4.1	63.8	105284
Rhodobacter sphaeroides 2.4.1	69	943016	Rhodobacter sphaeroides 2.4.1	63.8	100828
Rhodobacter sphaeroides ATCC 17025	68.5	3217726	Rhodobacter sphaeroides ATCC 17025 plasmid	67.7	877879
Rhodobacter sphaeroides ATCC 17025	68.5	3217726	Rhodobacter sphaeroides ATCC 17025 plasmid	67.6	289489
Rhodobacter sphaeroides ATCC 17025	68.5	3217726	Rhodobacter sphaeroides ATCC 17025 plasmid	69.4	121962
Rhodobacter sphaeroides ATCC 17025	68.5	3217726	Rhodobacter sphaeroides ATCC 17025 plasmid	64	36198
Rhodobacter sphaeroides ATCC 17025	68.5	3217726	Rhodobacter sphaeroides ATCC 17025 plasmid	58.9	13873
Rhodobacter sphaeroides ATCC 17029	69.1	3147721	Rhodobacter sphaeroides ATCC 17029 plasmid	69.6	122606
Rhodobacter sphaeroides ATCC 17029 chromosome 2	68.6	1219053	Rhodobacter sphaeroides ATCC 17029 plasmid	69.6	122606
Rhodobacter sphaeroides KD131	69.2	3152792	Rhodobacter sphaeroides KD131 plasmid pRSKD131A	70.1	157345

Rhodobacter sphaeroides KD131	69.2	3152792	Rhodobacter sphaeroides KD131 plasmid pRSKD131B	69.8	103355
Rhodobacter sphaeroides KD131 chromosome 2	68.7	1297647	Rhodobacter sphaeroides KD131 plasmid pRSKD131A	70.1	157345
Rhodobacter sphaeroides KD131 chromosome 2	68.7	1297647	Rhodobacter sphaeroides KD131 plasmid pRSKD131B	69.8	103355
Rhodococcus erythropolis PR4	62.3	6516310	Rhodococcus erythropolis PR4	61.9	271577
Rhodococcus erythropolis PR4	62.3	6516310	Rhodococcus erythropolis PR4	63	104014
Rhodococcus erythropolis PR4	62.3	6516310	Rhodococcus erythropolis PR4	62.2	3637
Rhodococcus jostii RHA1	67.5	7804765	Rhodococcus jostii RHA1 plasmid pRHL1	65.1	1123075
Rhodococcus jostii RHA1	67.5	7804765	Rhodococcus jostii RHA1 plasmid pRHL2	64	442536
Rhodococcus jostii RHA1	67.5	7804765	Rhodococcus jostii RHA1 plasmid pRHL3	64.9	332361
Rhodoferax ferrireducens T118	59.9	4712337	Rhodoferax ferrireducens T118	54.4	257447
Rhodospirillum rubrum ATCC11170	65.4	4352825	Rhodospirillum rubrum ATCC 11170 plasmid unnamed	59.8	53732
Rhodothermus marinus DSM 4252	64.5	3261604	Rhodothermus marinus DSM 4252 plasmid pRMAR01	58.2	125133
Rickettsia africae ESF-5	32.4	1278540	Rickettsia africae ESF-5 plasmid pRAF	33.5	12377
Rickettsia felis URRWXCal2	32.5	1485148	Rickettsia felis URRWXCal2 plasmid	33.6	62829

Rickettsia felis URRWXCal2	32.5	1485148	Rickettsia felis URRWXCal2 plasmid pRFdelta	33.2	39263
Rickettsia massiliae MTU5	32.5	1360898	Rickettsia massiliae MTU5 plasmid pRMA	31.7	15286
Rickettsia peacockii str. Rustic	32.6	1288492	Rickettsia peacockii str. Rustic plasmid pRPR	34.7	26406
Ruegeria pomeroyi DSS-3	64.2	4109442	Ruegeria pomeroyi DSS-3 megaplasmid	62.8	491611
Ruminococcus albus 7	44.2	3685408	Ruminococcus albus 7 plasmid pRUMAL01	38.1	420706
Ruminococcus albus 7	44.2	3685408	Ruminococcus albus 7 plasmid pRUMAL02	44.4	352646
Ruminococcus albus 7	44.2	3685408	Ruminococcus albus 7 plasmid pRUMAL03	36.7	15907
Ruminococcus albus 7	44.2	3685408	Ruminococcus albus 7 plasmid pRUMAL04	42.8	7420
Salinibacter ruber DSM 13855	66.2	3551823	Salinibacter ruber DSM 13855 plasmid pSR35	57.9	35505
Salmonella enterica subsp. enterica serovar Agona str. SL483	52.1	4798660	Salmonella enterica subsp. enterica serovar Agona str. SL483 plasmid unnamed	40.7	37978
Salmonella enterica subsp. enterica serovar Choleraesuis str. SC-B67	52.2	4755700	Salmonella enterica subsp. enterica serovar Choleraesuis plasmid pSCV50	52.1	49558
Salmonella enterica subsp. enterica serovar Choleraesuis str. SC-B67	52.2	4755700	Salmonella enterica subsp. enterica serovar Choleraesuis plasmid pSC138	51.3	138742
Salmonella enterica subsp. enterica serovar Dublin str. CT_02021853	52.2	4842908	Salmonella enterica subsp. enterica serovar Dublin str. CT_02021853 plasmid	48.6	74551

Salmonella enterica subsp. enterica serovar Heidelberg str. SL476	52.1	4888768	Salmonella enterica subsp. enterica serovar Heidelberg str. SL476 plasmid pSL476_91	50.3	91374
Salmonella enterica subsp. enterica serovar Heidelberg str. SL476	52.1	4888768	Salmonella enterica subsp. enterica serovar Heidelberg str. SL476 plasmid pSL476_3	55.1	3373
Salmonella enterica subsp. enterica serovar Newport str. SL254	52.2	4827641	Salmonella enterica enterica sv Newport str. SL254 plasmid	52.8	176473
Salmonella enterica subsp. enterica serovar Newport str. SL254	52.2	4827641	Salmonella enterica subsp. enterica serovar Newport str. SL254 plasmid pSL254_3	43	3605
Salmonella enterica subsp. enterica serovar Paratyphi C strain RKS4594	52.2	4833080	Salmonella enterica subsp. enterica serovar Paratyphi C strain RKS4594 plasmid	52.8	55414
Salmonella enterica subsp. enterica serovar Schwarzengrund str. CVM19633	52.2	4709075	Salmonella enterica subsp. enterica serovar Schwarzengrund str. CVM19633 plasmid pCVM19633_110	54	110227
Salmonella enterica subsp. enterica serovar Schwarzengrund str. CVM19633	52.2	4709075	Salmonella enterica subsp. enterica serovar Schwarzengrund str. CVM19633 plasmid pCVM19633_4	47.5	4585
Salmonella enterica subsp. enterica serovar Typhi	52.1	4809037	Salmonella enterica subsp. enterica serovar Typhi plasmid pHCM1	47.6	218160
Salmonella enterica subsp. enterica serovar Typhi	52.1	4809037	Salmonella enterica subsp. enterica serovar Typhi plasmid pHCM2	50.6	106516

Salmonella enterica subsp. enterica serovar Typhimurium LT2	52.2	4857432	Salmonella enterica subsp. enterica serovar Typhimurium LT2 plasmid pSLT	53.1	93939
Sebaldella termitidis ATCC 33386	33.5	4418842	Sebaldella termitidis ATCC 33386 plasmid pSTERM01	28.2	54160
Sebaldella termitidis ATCC 33386	33.5	4418842	Sebaldella termitidis ATCC 33386 plasmid pSTERMP2	29.4	13648
Serratia proteamaculans 568	55.1	5448853	Serratia proteamaculans 568 plasmid pSPRO01	49.2	46804
Shewanella baltica OS155	46.3	5127376	Shewanella baltica OS155 plasmid pSbal01	46.7	116763
Shewanella baltica OS155	46.3	5127376	Shewanella baltica OS155 plasmid pSbal02	42.4	74000
Shewanella baltica OS155	46.3	5127376	Shewanella baltica OS155 plasmid pSbal03	46.1	16762
Shewanella baltica OS155	46.3	5127376	Shewanella baltica OS155 plasmid pSbal04	40.3	7995
Shewanella baltica OS185	46.3	5229686	Shewanella baltica OS185 plasmid	43.7	83224
Shewanella baltica OS195	46.3	5347283	Shewanella baltica OS195 plasmid	42.7	75605
Shewanella baltica OS195	46.3	5347283	Shewanella baltica OS195 plasmid	44.1	75508
Shewanella baltica OS195	46.3	5347283	Shewanella baltica OS195 plasmid	42.9	49148
Shewanella baltica OS223	46.3	5145902	Shewanella baltica OS223 plasmid	44.4	88311
Shewanella baltica OS223	46.3	5145902	Shewanella baltica OS223 plasmid	46.7	59223
Shewanella baltica OS223	46.3	5145902	Shewanella baltica OS223 plasmid	44.6	65448

Shewanella oneidensis MR-1	46	4969803	Shewanella oneidensis MR-1 megaplasmid	43.7	161613
Shewanella sp. ANA-3	48.1	4972204	Shewanella sp. ANA-3 plasmid 1	45.7	278942
Shewanella sp. MR-7	47.9	4792610	Shewanella sp. MR-7 plasmid1	43.1	6499
Shigella boydii CDC 3083-94	51.3	4615997	Shigella boydii CDC 3083-94 plasmid	47.2	2089
Shigella boydii CDC 3083-94	51.3	4615997	Shigella boydii CDC 3083-94 plasmid	41.4	33103
Shigella boydii CDC 3083-94	51.3	4615997	Shigella boydii CDC 3083-94 plasmid	46.4	5114
Shigella boydii CDC 3083-94	51.3	4615997	Shigella boydii CDC 3083-94 plasmid	46.1	210919
Shigella boydii CDC 3083-94	51.3	4615997	Shigella boydii CDC 3083-94 plasmid	49.1	7437
Shigella boydii Sb227	51.2	4519823	Shigella boydii Sb227 plasmid pSB4 227	47.4	126697
Shigella dysenteriae Sd197	51.2	4369232	Shigella dysenteriae Sd197 plasmid	44.8	182726
Shigella dysenteriae Sd197	51.2	4369232	Shigella dysenteriae Sd197 plasmid	39.7	8953
Shigella flexneri 2a str 301	50.9	4607203	Shigella flexneri 2a str. 301 plasmid pCP301	45.8	221618
Shigella sonnei Ss046	51	4825265	Shigella sonnei Ss046 plasmid pSS	45.3	214396
Silicibacter sp. TM1040	60.4	3200938	Silicibacter sp. TM1040 mega plasmid	59.4	821788
Silicibacter sp. TM1040	60.4	3200938	Silicibacter sp. TM1040 plasmid unnamed	55.7	130973
Sinorhizobium medicae WSM419	61.5	3781904	Sinorhizobium medicae WSM419 plasmid pSMED01	61.5	1570951

Sinorhizobium medicae WSM419	61.5	3781904	Sinorhizobium medicae WSM419 plasmid pSMED02	59.9	1245408
Sinorhizobium medicae WSM419	61.5	3781904	inorhizobium medicae WSM419 plasmid pSMED03	60.1	219313
Sinorhizobium meliloti 1021	62.7	3654135	Sinorhizobium meliloti plasmid pSymA	60.4	1354226
Sinorhizobium meliloti 1021	62.7	3654135	Sinorhizobium meliloti plasmid pSymB	62.4	1683333
Sinorhizobium meliloti AK83	62.7	3820344	Sinorhizobium meliloti AK83 plasmid	58.9	256269
Sinorhizobium meliloti AK83	62.7	3820344	Sinorhizobium meliloti AK83 plasmid	57.9	70499
Sinorhizobium meliloti AK83 chromosome 2	62.4	1680879	Sinorhizobium meliloti AK83 plasmid	58.9	256269
Sinorhizobium meliloti AK83 chromosome 2	62.4	1680879	Sinorhizobium meliloti AK83 plasmid	57.9	70499
Sinorhizobium meliloti AK83 chromosome 3	60	1312480	Sinorhizobium meliloti AK83 plasmid	58.9	256269
Sinorhizobium meliloti AK83 chromosome 3	60	1312480	Sinorhizobium meliloti AK83 plasmid	57.9	70499
Sodalis glossinidius str. morsitans	54.7	4171146	Sodalis glossinidius str. morsitans plasmid pSG1	48.9	83306
Sodalis glossinidius str. morsitans	54.7	4171146	Sodalis glossinidius str. morsitans plasmid pSG2	44.7	27240
Sodalis glossinidius str. morsitans	54.7	4171146	Sodalis glossinidius str. 'morsitans' plasmid	47.8	10810
Sphingobium chlorophenolicum L-1	63.9	3080818	Sphingobium chlorophenolicum L-1 plasmid pSPHCH01	64.8	123733
Sphingobium chlorophenolicum L-1 chromosome 2	63.6	1368670	Sphingobium chlorophenolicum L-1 plasmid pSPHCH01	64.8	123733

Sphingobium japonicum UT26S	64.8	3514822	Sphingobium japonicum UT26S plasmid pUT1	63.7	31776
Sphingobium japonicum UT26S	64.8	3514822	Sphingobium japonicum UT26S plasmid pCHQ1	63	190974
Sphingobium japonicum UT26S	64.8	3514822	Sphingobium japonicum UT26S plasmid pUT2	61	5398
Sphingobium japonicum UT26S chromosome 2	65.9	681892	Sphingobium japonicum UT26S plasmid pUT1	63.7	31776
Sphingobium japonicum UT26S chromosome 2	65.9	681892	Sphingobium japonicum UT26S plasmid pCHQ1	63	190974
Sphingobium japonicum UT26S chromosome 2	65.9	681892	Sphingobium japonicum UT26S plasmid pUT2	61	5398
Sphingomonas wittichii RW1	68.4	5382261	Sphingomonas wittichii RW1 plasmid pSWIT01	64.1	310228
Sphingomonas wittichii RW1	68.4	5382261	Sphingomonas wittichii RW1 plasmid pSWIT02	61.2	222757
Sphingopyxis alaskensis RB2256	65.5	3345170	Sphingopyxis alaskensis RB2256 F plasmid	60.4	28543
Spirosoma linguale DSM 74	50.2	8078757	Spirosoma linguale DSM 74 plasmid	49.2	189452
Spirosoma linguale DSM 74	50.2	8078757	Spirosoma linguale DSM 74 plasmid	51.3	146936
Spirosoma linguale DSM 74	50.2	8078757	Spirosoma linguale DSM 74 plasmid	44.3	36434
Spirosoma linguale DSM 74	50.2	8078757	Spirosoma linguale DSM 74 plasmid	47.8	9965
Spirosoma linguale DSM 74	50.2	8078757	Spirosoma linguale DSM 74 plasmid	44.2	8651
Spirosoma linguale DSM 74	50.2	8078757	Spirosoma linguale DSM 74 plasmid	47.3	7683
Spirosoma linguale DSM 74	50.2	8078757	Spirosoma linguale DSM 74 plasmid	47.6	7308
Spirosoma linguale DSM 74	50.2	8078757	Spirosoma linguale DSM 74 plasmid	48.4	6072

Staphylococcus aureus strain Mu50	32.9	2878529	Staphylococcus aureus subsp. aureus Mu50 plasmid VRSAp	28.9	25107
Staphylococcus aureus subsp. aureus COL	32.8	2809422	Staphylococcus aureus subsp. aureus COL plasmid pT181	30	4440
Staphylococcus aureus subsp. aureus ED98	32.8	2824404	Staphylococcus aureus subsp. aureus ED98 plasmid pAVY	35.6	1442
Staphylococcus aureus subsp. aureus ED98	32.8	2824404	Staphylococcus aureus subsp. aureus ED98 plasmid pT181	30.1	4440
Staphylococcus aureus subsp. aureus ED98	32.8	2824404	Staphylococcus aureus subsp. aureus ED98 plasmid pAVX	29	17256
Staphylococcus aureus subsp. aureus JH1	33	2906507	Staphylococcus aureus subsp. aureus JH1 plasmid pSJH101	29.6	30429
Staphylococcus aureus subsp. aureus JH9	32.9	2906700	Staphylococcus aureus subsp. aureus JH9 plasmid pSJH901	29.6	30429
Staphylococcus aureus subsp. aureus N315	32.8	2814816	Staphylococcus aureus subsp. aureus N315 plasmid pN315	28.7	24653
Staphylococcus aureus subsp. aureus USA300_FPR3757	32.8	2872769	Staphylococcus aureus subsp. aureus USA300_FPR3757 plasmid pUSA01	28.7	3125
Staphylococcus aureus subsp. aureus USA300_FPR3757	32.8	2872769	Staphylococcus aureus subsp. aureus USA300_FPR3757 plasmid pUSA02	30	4439

Staphylococcus aureus subsp. aureus USA300_FPR3757	32.8	2872769	Staphylococcus aureus subsp. aureus USA300 plasmid USA300_FPR3757	28.7	37136
Staphylococcus aureus subsp. aureus USA300_TCH1516	32.8	2872915	Staphylococcus aureus subsp. aureus USA300_TCH1516 plasmid pUSA300HOUMR	30.5	27041
Staphylococcus epidermidis RP62A	32.2	2616530	Staphylococcus epidermidis RP62A plasmid pSERP	31.9	27310
Staphylococcus saprophyticus subsp. saprophyticus	33.2	2516575	Staphylococcus saprophyticus subsp. saprophyticus plasmid pSSP1	30.8	38454
Staphylococcus saprophyticus subsp. saprophyticus	33.2	2516575	Staphylococcus saprophyticus subsp. saprophyticus plasmid pSSP2	31.3	22870
Streptobacillus moniliformis DSM 12112	26.3	1662578	Streptobacillus moniliformis DSM 12112 plasmid	20.9	10702
Streptococcus gallolyticus subsp. gallolyticus ATCC	37.6	2356444	Streptococcus gallolyticus subsp. gallolyticus ATCC	37.3	20765
Streptococcus suis BM407	41.1	2146229	Streptococcus suis BM407 plasmid	36.4	24579
Streptococcus thermophilus LMD-9	39.1	1856368	Streptococcus thermophilus LMD-9 plasmid 1	37	4449
Streptococcus thermophilus LMD-9	39.1	1856368	Streptococcus thermophilus LMD-9 plasmid 2	35.1	3361

Streptomyces avermitilis MA-4680	70.7	9025608	Streptomyces avermitilis MA-4680	69.2	94287
Streptomyces coelicolor A3(2)	72.1	8667507	Streptomyces coelicolor A3(2) plasmid SCP1	69.1	356023
Streptomyces coelicolor A3(2)	72.1	8667507	Streptomyces coelicolor A3(2) plasmid SCP2	72.1	31317
Streptosporangium roseum DSM 43021	70.9	10341314	Streptosporangium roseum DSM 43021 plasmid pSROS01	69.6	28204
Sulfuricurvum kujiense DSM 16994	45	2574824	Sulfuricurvum kujiense DSM 16994 plasmid pSULKU01	40.6	118585
Sulfuricurvum kujiense DSM 16994	45	2574824	Sulfuricurvum kujiense DSM 16994 plasmid pSULKU02	40.3	71513
Sulfuricurvum kujiense DSM 16994	45	2574824	Sulfuricurvum kujiense DSM 16994 plasmid pSULKU03	38.2	51014
Sulfuricurvum kujiense DSM 16994	45	2574824	Sulfuricurvum kujiense DSM 16994 plasmid pSULKU04	43.1	3421
Synechococcus elongatus PCC 7942	55.5	2695903	Synechococcus elongatus PCC 7942	52.9	46366
Synechococcus sp. PCC 7002	49.6	3008047	Synechococcus sp. PCC 7002 plasmid pAQ1	49	4809
Synechococcus sp. PCC 7002	49.6	3008047	Synechococcus sp. PCC 7002 plasmid pAQ3	45.9	16103
Synechococcus sp. PCC 7002	49.6	3008047	Synechococcus sp. PCC 7002 plasmid pAQ4	44.1	31972
Synechococcus sp. PCC 7002	49.6	3008047	Synechococcus sp. PCC 7002 plasmid pAQ5	42.6	38515
Synechococcus sp. PCC 7002	49.6	3008047	Synechococcus sp. PCC 7002 plasmid pAQ6	45.1	124030
Synechococcus sp. PCC 7002	49.6	3008047	Synechococcus sp. PCC 7002 plasmid pAQ7	47.3	186459

Thauera sp. MZ1T	68.4	4496212	Thauera sp. MZ1T plasmid pTha01	62.3	78374
Thermomicrobium roseum DSM 5159	63.6	2003006	Thermomicrobium roseum DSM 5159 plasmid unnamed	65.7	917738
Thermovibrio ammonificans HB-1	52.1	1682965	Thermovibrio ammonificans HB-1 plasmid pTHEAM01	52.5	76561
Thermus scotoductus SA-01	64.9	2346803	Thermus scotoductus SA-01 plasmid pTSC8	65.9	8383
Thermus thermophilus HB27	69.4	1894877	Thermus thermophilus HB27 plasmid pTT27	69.2	232605
Thermus thermophilus HB8	69.5	1849742	Thermus thermophilus HB8 plasmid pTT27	69.4	256992
Thermus thermophilus HB8	69.5	1849742	Thermus thermophilus HB8 plasmid pTT8	69	9322
Thiomonas intermedia K12	63.9	3396378	Thiomonas intermedia K12 plasmid pTINT01	61.5	45943
Thiomonas intermedia K12	63.9	3396378	Thiomonas intermedia K12 plasmid pTINT02	60.1	19774
Treponema succinifaciens DSM 2489	39.2	2731853	Treponema succinifaciens DSM 2489 plasmid	38.7	165572
Tsukamurella paurometabola DSM 20162	68.4	4379918	Tsukamurella paurometabola DSM 20162 plasmid pTpau01	67.8	99806
Verminephrobacter eiseniae EF01-2	65.3	5566749	Verminephrobacter eiseniae EF01-2 plasmid pVEIS01	58.6	31194
Verrucosispora maris AB-18-032	70.9	6673976	Verrucosispora maris AB-18-032 plasmid	70.3	58295
Vibrio fischeri MJ11	38.9	2905029	Vibrio fischeri MJ11 plasmid pMJ100	34.8	179459
Vibrio fischeri MJ11 chromosome II	37.2	1418848	Vibrio fischeri MJ11 plasmid pMJ100	34.8	179459

Vibrio harveyi ATCC BAA-1116	45.5	3765351	Vibrio harveyi ATCC BAA-1116 plasmid pVIBHAR	43.8	89008
Vibrio harveyi ATCC BAA-1116 chromosome II	45.3	2204018	Vibrio harveyi ATCC BAA-1116 plasmid pVIBHAR	43.8	89008
Vibrio vulnificus YJ016	46.4	3354505	Vibrio vulnificus YJ016 plasmid pYJ016	44.9	48508
Vibrio vulnificus YJ016 chromosome II	47.2	1857073	Vibrio vulnificus YJ016 plasmid pYJ016	44.9	48508
Waddlia chondrophila WSU 86-1044	43.8	2116312	Waddlia chondrophila WSU 86-1044 plasmid pWc	37.6	15593
Xanthobacter autotrophicus Py2	67.5	5308934	Xanthobacter autotrophicus Py2 plasmid pXAUT01	65.3	316164
Xanthomonas campestris pv. vesicatoria str. 85-10	64.7	5178466	Xanthomonas campestris pv. vesicatoria str. 85-10	60.5	182572
Xanthomonas campestris pv. vesicatoria str. 85-10	64.7	5178466	Xanthomonas campestris pv. vesicatoria str. 85-10	60.7	38116
Xanthomonas campestris pv. vesicatoria str. 85-10	64.7	5178466	Xanthomonas campestris pv. vesicatoria str. 85-10	59.8	19146
Xanthomonas campestris pv. vesicatoria str. 85-10	64.7	5178466	Xanthomonas campestris pv. vesicatoria str. 85-10	56.6	1852
Xenorhabdus nematophila ATCC 19061	44.2	4432590	Xenorhabdus nematophila ATCC 19061 plasmid XNC1 p	46	155327
Xylanimonas cellulosilytica DSM 15894	72.5	3742776	Xylanimonas cellulosilytica DSM 15894 plasmid	71	88604

Xylella fastidiosa	51.8	2519802	Xylella fastidiosa Temecula1 plasmid pXFPD1.3	53.8	1346
Xylella fastidiosa 9a5c	52.7	2679306	Xylella fastidiosa plasmid pXF51	49.6	51158
Xylella fastidiosa 9a5c	52.7	2679306	Xylella fastidiosa plasmid pXF1.3	55.6	1286
Xylella fastidiosa M23	51.8	2535690	Xylella fastidiosa M23 plasmid pXFAS01	49.2	38297
Yersinia enterocolitica subsp. enterocolitica 8081	47.3	4615899	Yersinia enterocolitica subsp. enterocolitica 8081 plasmid	43.9	67721
Yersinia enterocolitica subsp. palearctica 105.5R(r)	47	4552107	Yersinia enterocolitica subsp. palearctica 105.5R(r) plasmid 105.5R(r)p	44.2	69704
Yersinia pestis Angola	47.6	4504254	Yersinia pestis Angola plasmid new pCD	44.6	68190
Yersinia pestis Angola	47.6	4504254	Yersinia pestis Angola plasmid pMT-pPCP	50	114570
Yersinia pestis Antiqua	47.7	4702289	Yersinia pestis Antiqua plasmid pMT	50.2	96471
Yersinia pestis Antiqua	47.7	4702289	Yersinia pestis Antiqua plasmid pPCP	45.4	10777
Yersinia pestis Antiqua	47.7	4702289	Yersinia pestis Antiqua plasmid pCD	44.8	70299
Yersinia pestis Nepal516	47.6	4534590	Yersinia pestis Nepal516 plasmid pMT	50.2	100918
Yersinia pestis Nepal516	47.6	4534590	Yersinia pestis Nepal516 plasmid pPCP	45.4	10778
Yersinia pestis Pestoides F	47.6	4517345	Yersinia pestis Pestoides F plasmid CD	44.9	71507
Yersinia pestis Pestoides F	47.6	4517345	Yersinia pestis Pestoides F plasmid MT	52	137010

Yersinia pestis Z176003	47.7	4553586	Yersinia pestis Z176003 plasmid pCD1	44.6	68342
Yersinia pestis Z176003	47.7	4553586	Yersinia pestis Z176003 plasmid pPCP1	45.3	9609
Yersinia pestis biovar Microtus str. 91001	47.7	4595065	Yersinia pestis biovar Microtus str. 91001 plasmid pCRY	49.1	21742
Yersinia pestis biovar Microtus str. 91001	47.7	4595065	Yersinia pestis biovar Microtus str. 91001 plasmid pCD1	44.8	70159
Yersinia pestis biovar Microtus str. 91001	47.7	4595065	Yersinia pestis biovar Microtus str. 91001 plasmid pMT1	50.3	106642
Yersinia pestis biovar Microtus str. 91001	47.7	4595065	Yersinia pestis biovar Microtus str. 91001 plasmid pPCP1	45.3	9609
Yersinia pestis strain CO92	47.6	4653728	Yersinia pestis plasmid pCD1	44.8	70305
Yersinia pestis strain CO92	47.6	4653728	Yersinia pestis plasmid pPCP1	45.3	9612
Yersinia pestis strain CO92	47.6	4653728	Yersinia pestis plasmid pMT1	50.2	96210
Yersinia pseudotuberculosis IP 31758	47.5	4723306	Yersinia pseudotuberculosis IP 31758 plasmid 59kb	40.2	58679
Yersinia pseudotuberculosis IP 31758	47.5	4723306	Yersinia pseudotuberculosis IP 31758 plasmid 153kb	40.4	153140
Yersinia pseudotuberculosis IP 32953	47.6	4744671	Yersinia pseudotuberculosis IP 32953 plasmid pYV	44.6	68525
Yersinia pseudotuberculosis IP 32953	47.6	4744671	Yersinia pseudotuberculosis IP 32953 plasmid	44.6	27702

Yersinia pseudotuberculosis PB1/+	47.5	4695619	Yersinia pseudotuberculosis PB1/+ plasmid	44.7	69812
Zymomonas mobilis subsp. mobilis NCIB 11163	46.8	2124771	Zymomonas mobilis subsp. mobilis NCIB 11163 plasmid	42.3	53380
Zymomonas mobilis subsp. mobilis NCIB 11163	46.8	2124771	Zymomonas mobilis subsp. mobilis NCIB 11163 plasmid	43.8	40818
Zymomonas mobilis subsp. mobilis NCIB 11163	46.8	2124771	Zymomonas mobilis subsp. mobilis NCIB 11163 plasmid	36.4	4551
