# Supplementary material for: Comparative Analyses of Base Compositions, DNA Sizes, and Dinucleotide Frequency Profiles in Archaeal and Bacterial Chromosomes and Plasmids
Source: Int J Evol Biol. 2012 Mar 26;2012:342482. doi: 10.1155/2012/342482 (PMC3321278; doi:10.1155/2012/342482)
Supplement: Supplementary file 2 [file 342482.f2.pdf]

Supplementary Table S2. Pairs of chromosome and plasmid in archaea.

Organism	Chromosome GC %	Chromosome size (bp)	Plasmid	Plasmid GC %	Plasmid size (bp)
Archaeoglobus profundus DSM 5631	42	1560622	Archaeoglobus profundus DSM 5631	39.8	2801
Halalkalicoccus jeotgali B3	65	2809118	Halalkalicoccus jeotgali B3 plasmid 1	55.3	406285
Halalkalicoccus jeotgali B3	65	2809118	Halalkalicoccus jeotgali B3 plasmid 2	54.2	363534
Halalkalicoccus jeotgali B3	65	2809118	Halalkalicoccus jeotgali B3 plasmid 3	58.9	44576
Halalkalicoccus jeotgali B3	65	2809118	Halalkalicoccus jeotgali B3 plasmid 4	47.6	23727
Halalkalicoccus jeotgali B3	65	2809118	Halalkalicoccus jeotgali B3 plasmid 5	47.6	23727
Halalkalicoccus jeotgali B3	65	2809118	Halalkalicoccus jeotgali B3 plasmid 6	60.6	6951
Haloarcula marismortui ATCC 43049	62.4	3131724	Haloarcula marismortui ATCC 43049 plasmid	54.3	33303
Haloarcula marismortui ATCC 43049	62.4	3131724	Haloarcula marismortui ATCC 43049 plasmid	55.6	33452
Haloarcula marismortui ATCC 43049	62.4	3131724	Haloarcula marismortui ATCC 43049 plasmid	60	39521
Haloarcula marismortui ATCC 43049	62.4	3131724	Haloarcula marismortui ATCC 43049 plasmid	57.3	50060
Haloarcula marismortui ATCC 43049	62.4	3131724	Haloarcula marismortui ATCC 43049 plasmid	54.5	132678
Haloarcula marismortui ATCC 43049	62.4	3131724	Haloarcula marismortui ATCC 43049 plasmid	58.3	155300
Haloarcula marismortui ATCC 43049	62.4	3131724	Haloarcula marismortui ATCC 43049 plasmid	59.1	410554
Haloarcula marismortui ATCC 43049	57.2	288050	Haloarcula marismortui ATCC 43049 plasmid	54.3	33303
Haloarcula marismortui ATCC 43049	57.2	288050	Haloarcula marismortui ATCC 43049 plasmid	55.6	33452

Haloarcula marismortui ATCC 43049	57.2	288050	Haloarcula marismortui ATCC 43049 plasmid	60	39521
Haloarcula marismortui ATCC 43049	57.2	288050	Haloarcula marismortui ATCC 43049 plasmid	57.3	50060
Haloarcula marismortui ATCC 43049	57.2	288050	Haloarcula marismortui ATCC 43049 plasmid	54.5	132678
Haloarcula marismortui ATCC 43049	57.2	288050	Haloarcula marismortui ATCC 43049 plasmid	58.3	155300
Haloarcula marismortui ATCC 43049	57.2	288050	Haloarcula marismortui ATCC 43049 plasmid	59.1	410554
Halobacterium salinarum R1	68	2000962	Halobacterium salinarum R1 plasmid	57.4	147625
Halobacterium salinarum R1	68	2000962	Halobacterium salinarum R1 plasmid	58.6	194963
Halobacterium salinarum R1	68	2000962	Halobacterium salinarum R1 plasmid	59.8	284332
Halobacterium salinarum R1	68	2000962	Halobacterium salinarum R1 plasmid	57.9	40894
Halobacterium sp. NRC-1	67.9	2014239	Halobacterium sp. NRC-1 plasmid pNRC200	59.2	365425
Halobacterium sp. NRC-1	67.9	2014239	Halobacterium sp. NRC-1 plasmid pNRC100	57.9	191346
Haloferax volcanii DS2	66.6	2847757	Haloferax volcanii DS2 plasmid pHV1	55.5	85092
Haloferax volcanii DS2	66.6	2847757	Haloferax volcanii DS2 plasmid pHV2	56.1	6359
Haloferax volcanii DS2	66.6	2847757	Haloferax volcanii DS2 plasmid pHV3	65.6	437906
Haloferax volcanii DS2	66.6	2847757	Haloferax volcanii DS2 plasmid pHV4	61.7	635786
Halogeometricum borinquense DSM 11551	61.1	2820544	Halogeometricum borinquense DSM 11551	56.1	362194
Halogeometricum borinquense DSM 11551	61.1	2820544	Halogeometricum borinquense DSM 11551	57.3	339010

Halogeometricum borinquense DSM 11551	61.1	2820544	Halogeometricum borinquense DSM 11551	56.5	210350
Halogeometricum borinquense DSM 11551	61.1	2820544	Halogeometricum borinquense DSM 11551	58.8	194834
Halogeometricum borinquense DSM 11551	61.1	2820544	Halogeometricum borinquense DSM 11551	64.6	17535
Halomicrobium mukohataei DSM 12286	65.6	3110487	Halomicrobium mukohataei DSM 12286	64.2	221862
Halopiger xanaduensis SH-6	66	3668009	Halopiger xanaduensis SH-6 plasmid	61.5	436718
Halopiger xanaduensis SH-6	66	3668009	Halopiger xanaduensis SH-6 plasmid	59	181778
Halopiger xanaduensis SH-6	66	3668009	Halopiger xanaduensis SH-6 plasmid	62.2	68763
Haloquadratum walsbyi DSM 16790	47.9	3132494	Haloquadratum walsbyi DSM 16790 plasmid	47.7	46867
Halorubrum lacusprofundi ATCC	66.7	2735295	Halorubrum lacusprofundi ATCC	54.9	431338
Halorubrum lacusprofundi ATCC	57.1	525943	Halorubrum lacusprofundi ATCC	54.9	431338
Haloterrigena turkmenica DSM 5511	65.8	3889038	Haloterrigena turkmenica DSM 5511	61.2	698495
Haloterrigena turkmenica DSM 5511	65.8	3889038	Haloterrigena turkmenica DSM 5511	59.8	413648
Haloterrigena turkmenica DSM 5511	65.8	3889038	Haloterrigena turkmenica DSM 5511	54.7	180781
Haloterrigena turkmenica DSM 5511	65.8	3889038	Haloterrigena turkmenica DSM 5511	64.6	171943
Haloterrigena turkmenica DSM 5511	65.8	3889038	Haloterrigena turkmenica DSM 5511	60	71062
Haloterrigena turkmenica DSM 5511	65.8	3889038	Haloterrigena turkmenica DSM 5511	58.1	15815
Methanocaldococcus fervens AG86	32.2	1485061	Methanocaldococcus fervens AG86 plasmid	32.8	22190

Methanocaldococcus sp. FS406-22	32	1760939	Methanocaldococcus sp. FS406-22 plasmid	31.6	12197
Methanocaldococcus vulcanius M7	31.5	1746329	Methanocaldococcus vulcanius M7 plasmid	46.3	10704
Methanocaldococcus vulcanius M7	31.5	1746329	Methanocaldococcus vulcanius M7 plasmid	30.1	4704
Methanococcus jannaschii	31.4	1664970	Methanococcus jannaschii large extra-	28.2	58407
Methanococcus jannaschii	31.4	1664970	Methanococcus jannaschii small extra-	28.9	16550
Methanococcus maripaludis C5	33	1780761	Methanococcus maripaludis C5 plasmid	27.2	8285
Methanohalobium evestigatum Z-7303	36.6	2242317	Methanohalobium evestigatum Z-7303	33.6	163915
Methanosaeta concilii GP-6	51	3008626	Methanosaeta concilii GP-6 plasmid pGP6	43.9	18019
Methanosarcina barkeri str. fusaro	39.3	4837408	Methanosarcina barkeri str. fusaro plasmid 1	33.6	36358
Methanothermobacter marburgensis str. Marburg	48.6	1634695	Methanothermobacter marburgensis str. Marburg plasmid	45.4	4440
Methanothermococcus okinawensis IH1	29.3	1662525	Methanothermococcus okinawensis IH1 plasmid pMETOK01	26.8	14930
Natrialba magadii ATCC 43099	61.4	3751858	Natrialba magadii ATCC 43099 plasmid	60.1	378348
Natrialba magadii ATCC 43099	61.4	3751858	Natrialba magadii ATCC 43099 plasmid	61.9	58487
Natronomonas pharaonis DSM 2160	63.4	2595221	Natronomonas pharaonis DSM 2160 plasmid	57.2	130989
Natronomonas pharaonis DSM 2160	63.4	2595221	Natronomonas pharaonis DSM 2160 plasmid	60.6	23486
Sulfolobus islandicus L.D.8.5	35.3	2722032	Sulfolobus islandicus L.D.8.5 plasmid	36.1	26615

Sulfolobus islandicus Y.N.15.51	35.3	2812165	Sulfolobus islandicus Y.N.15.51 plasmid	36.1	42245
