# Supplementary material for: Comparative Analyses of Base Compositions, DNA Sizes, and Dinucleotide Frequency Profiles in Archaeal and Bacterial Chromosomes and Plasmids
Source: Int J Evol Biol. 2012 Mar 26;2012:342482. doi: 10.1155/2012/342482 (PMC3321278; doi:10.1155/2012/342482)
Supplement: Supplementary file 3 [file 342482.f3.pdf]

Supplementary Table S3. Bacterial chromosomes compared in this analysis.

Organism	GC content (%)	Chromosome size (bp)	Plasmid
Acaryochloris marina MBIC11017	47.3	6503724	present
Acetobacter pasteurianus IFO 3283-	53	2907495	absent
Acetohalobium arabaticum DSM 5501	36.6	2469596	absent
Acholeplasma laidlawii	31.9	1496992	absent
Achromobacter xylosoxidans A8	66	7013095	present
Acidaminococcus fermentans DSM 20731	55.8	2329769	absent
Acidimicrobium ferrooxidans DSM	68.3	2158157	absent
Acidiphilium cryptum	68	3389227	present
Acidiphilium multivorum AIU301	67.6	3749411	present
Acidithiobacillus ferrooxidans ATCC	58.8	2982397	absent
Acidithiobacillus ferrooxidans ATCC	58.9	2885038	absent
Acidobacterium capsulatum ATCC	60.5	4127356	absent
Acidobacterium sp. MP5ACTX9	60.5	4309153	present
Acidothermus cellulolyticus 11B	66.9	2443540	absent
Acidovorax avenae subsp. avenae ATCC	68.8	5482170	absent
Acidovorax avenae subsp. citrulli AAC00-1	68.5	5352772	absent
Acidovorax sp. JS42	66.2	4448856	present
Acinetobacter baumannii AB0057	39.2	4050513	present
Acinetobacter baumannii AB307-0294	39	3760981	absent
Acinetobacter baumannii ACICU	39	3904116	present
Acinetobacter baumannii ATCC	38.9	3976747	present
Acinetobacter	39.4	3936291	present
Acinetobacter	39.2	3421954	present
Acinetobacter sp. ADP1	40.4	3598621	absent
Acinetobacter sp. DR1	38.7	4152543	absent
Actinobacillus pleuropneumoniae L20	41.3	2274482	absent
Actinobacillus pleuropneumoniae serovar 3 str. JL03	41.2	2242062	absent
Actinobacillus pleuropneumoniae serovar 7 str. AP76	41.2	2331981	present

Actinobacillus succinogenes 130Z	44.9	2319663	absent
Actinosynnema mirum DSM 43827	73.7	8248144	absent
Aerococcus urinae ACS-120-V-Col10a	41.9	2080974	absent
Aeromonas hydrophila subsp. hydrophila	61.5	4744448	absent
Aeromonas salmonicida subsp. salmonicida	58.5	4702402	absent
Aeromonas veronii	58.7	4551783	absent
Aggregatibacter actinomycetemcomitans D11S-1	44.6	2105764	absent
Aggregatibacter aphrophilus NJ8700	42.2	2313035	absent
Agrobacterium radiobacter K84	60.3	4005130	present
Agrobacterium radiobacter K84	59.8	2650913	present
Agrobacterium sp. H13-	58.8	2823930	present
Agrobacterium tumefaciens strain C58	59.4	2841580	absent
Agrobacterium tumefaciens strain C58 (Cereon) linear	59.3	2075577	absent
Agrobacterium vitis S4	57.7	3726375	present
Agrobacterium vitis S4 chromosome 2	57.5	1283187	present
Akkermansia muciniphila ATCC	55.8	2664102	absent
Alcanivorax	54.7	3120143	absent
Alicycliphilus	68.3	4637013	present
Alicycliphilus denitrificans K601	68	4995263	present
Alicyclobacillus acidocaldarius subsp.	62.3	3018755	present
Aliivibrio salmonicida LFI1238 chromosome	40.1	83540	present
Aliivibrio salmonicida LFI1238 chromosome 1	39.2	3325165	present
Aliivibrio salmonicida LFI1238 chromosome 2	38.2	1206461	present
Alkalilimnicola ehrlichei MLHE-1	67.5	3275944	absent
Alkaliphilus metalliredigens QYMF	36.8	4929566	absent
Alkaliphilus oremlandii	36.3	3123558	absent
Allochromatium vinosum DSM 180	64.4	3526903	present
Alpha proteobacterium IMCC1322	48.9	2753527	absent
Alteromonas macleodii Deep ecotype	44.9	4412282	absent

Alteromonas sp. SN2	43.5	4972148	absent
Aminobacterium colombiense DSM	45.3	1980592	absent
Ammonifex degensii	59.4	2129237	present
Amycolatopsis mediterranei U32	71.3	10236715	absent
Amycolicicoccus subflavus DQS3-9A1	62.2	4738809	present
Anabaena variabilis ATCC 29413	46.5	37151	present
Anabaena variabilis ATCC 29413	41.4	6365727	present
Anaerocellum thermophilum DSM	35.2	2919718	present
Anaerococcus prevotii DSM 20548	36.1	1883067	present
Anaerolinea thermophila UNI-1	53.8	3532378	absent
Anaeromyxobacter dehalogenans 2CP-1	74.7	5029329	absent
Anaeromyxobacter dehalogenans 2CP-C	74.9	5013479	absent
Anaeromyxobacter sp. Fw109-5	73.5	5277990	absent
Anaeromyxobacter sp.	74.8	5061632	absent
Anaplasma centrale str.	50	1206806	absent
Anaplasma marginale	49.8	1202435	absent
Anaplasma marginale str St. Maries	49.8	1197687	absent
Anaplasma phagocytophilum HZ	41.6	1471282	absent
Anoxybacillus flavithermus WK1	41.8	2846746	absent
Aquifex aeolicus	43.5	1551335	present
Arcanobacterium haemolyticum DSM	53.1	1986154	absent
Arcobacter butzleri	27	2341251	absent
Arcobacter nitrofigilis DSM 7299	28.4	3192235	absent
Aromatoleum aromaticum EbN1	65.1	4296230	absent
Arthrobacter arilaitensis	59.3	3859257	present
Arthrobacter aurescens	62.3	4597686	present
Arthrobacter chlorophenolicus A6	66.3	4395537	present
Arthrobacter phenanthrenivorans	65.6	4250414	present
Arthrobacter sp. FB24	65.5	4698945	present
Aster yellows witches-broom phytoplasma	26.9	706569	present
Asticcacaulis excentricus CB 48	59.2	2588221	present
Asticcacaulis excentricus CB 48	60.4	1315949	present

Atopobium parvulum DSM 20469	45.7	1543805	absent
Azoarcus sp. BH72	67.9	4376040	absent
Azorhizobium caulinodans ORS 571	67.3	5369772	absent
Azospirillum sp. B510	67.8	3311395	present
Azotobacter vinelandii	65.7	5365318	absent
Bacillus amyloliquefaciens	46.1	3980199	absent
Bacillus amyloliquefaciens	46.5	3918589	absent
Bacillus anthracis CI	35.4	5196054	present
Bacillus anthracis str.	35.4	5227419	present
Bacillus anthracis str.	35.4	5227293	absent
Bacillus anthracis str. Ames 0581	35.4	5227419	present
Bacillus anthracis str. CDC 684	35.4	5230115	present
Bacillus anthracis str.	35.4	5228663	absent
Bacillus atrophaeus	43.2	4168266	absent
Bacillus cellulosilyticus DSM 2522	36.5	4681672	absent
Bacillus cereus	35.4	5269628	present
Bacillus cereus AH187	35.6	5269030	present
Bacillus cereus AH820	35.4	5302683	present
Bacillus cereus ATCC	35.6	5224283	absent
Bacillus cereus ATCC	35.3	5411809	present
Bacillus cereus B4264	35.3	5419036	absent
Bacillus cereus G9842	35.3	5387334	present
Bacillus cereus Q1	35.6	5214195	present
Bacillus cereus subsp. cytotoxis NVH 391-98	35.9	4087024	present
Bacillus cereus ZK	35.4	5300915	absent
Bacillus clausii KSM-	44.8	4303871	absent
Bacillus coagulans 2-6	47.3	3073079	absent
Bacillus halodurans	43.7	4202352	absent
Bacillus licheniformis ATCC 14580	46.2	4222597	absent
Bacillus licheniformis	46.2	4222645	absent
Bacillus megaterium	38.1	5097447	absent
Bacillus megaterium QM B1551	38.3	5097129	present
Bacillus pseudofirmus	40.3	3858997	present
Bacillus pumilus	41.3	3704465	absent
Bacillus selenitireducens MLS10	48.7	3592487	absent
Bacillus subtilis	43.5	4215606	absent
Bacillus subtilis BSn5	43.8	4093599	absent
Bacillus subtilis subsp. spizizenii str. W23	43.9	4027676	absent
Bacillus thuringiensis	35.4	5237682	absent
Bacillus thuringiensis	35.3	5330088	present
Bacillus thuringiensis str. Al Hakam	35.4	5257091	present

Bacillus tusciae DSM	59.1	3384766	absent
Bacillus weihenstephanensis	35.6	5262775	present
Bacteroides fragilis NCTC 9343	43.2	5205140	present
Bacteroides fragilis	43.3	5277274	present
Bacteroides helcogenes P 36-108	44.7	3998906	absent
Bacteroides salanitronis DSM 18170	46.6	4242803	present
Bacteroides thetaiotaomicron VPI-	42.8	6260361	absent
Bacteroides vulgatus ATCC 8482	42.2	5163189	absent
Bartonella bacilliformis	38.2	1445021	absent
Bartonella clarridgeiae	35.7	1522743	absent
Bartonella grahamii	38.1	2341328	present
Bartonella henselae str. Houston-1	38.2	1931047	absent
Bartonella quintana str. Toulouse	38.8	1581384	absent
Bartonella tribocorum CIP 105476	38.9	2619061	present
Baumannia cicadellinicola str. Hc	33.2	686194	absent
Bdellovibrio bacteriovorus HD100	50.6	3782950	absent
Beijerinckia indica subsp. indica ATCC	57.1	4170153	present
Beutenbergia cavernae DSM 12333	73.1	4669183	absent
Bifidobacterium adolescentis ATCC	59.2	2089645	absent
Bifidobacterium animalis subsp. lactis	60.5	1933695	absent
Bifidobacterium animalis subsp. lactis	60.5	1938709	absent
Bifidobacterium animalis subsp. lactis	60.5	1938483	absent
Bifidobacterium bifidum PRL2010	62.7	2214656	absent
Bifidobacterium	62.8	2186882	absent
Bifidobacterium	58.5	2636367	absent
Bifidobacterium longum DJO10A	60.1	2375792	present
Bifidobacterium longum NCC2705	60.1	2256640	absent
Bifidobacterium longum subsp. infantis	60.1	2400312	present
Bifidobacterium longum subsp. infantis	59.9	2832748	absent
Bifidobacterium longum subsp. longum	59.9	2265943	absent

Bifidobacterium longum subsp. longum	60.3	2385164	absent
Bifidobacterium longum subsp. longum	59.8	2477838	absent
Blattabacterium sp. (Blattella germanica)	27.1	636850	absent
Blattabacterium sp. (Periplaneta americana) str. BPLAN	28.2	636994	present
Blochmannia floridanus	27.4	705557	absent
Blochmannia pennsylvanicus str.	29.6	791654	absent
Bordetella avium 197N	61.6	3732255	absent
Bordetella	68.1	5339179	absent
Bordetella parapertussis	68.1	4773551	absent
Bordetella pertussis	67.7	4086189	absent
Bordetella petrii	65.5	5287950	absent
Borrelia afzelii PKo	28.3	905394	present
Borrelia burgdorferi	28.6	910724	present
Borrelia burgdorferi	28.5	906707	present
Borrelia duttonii Ly	27.6	931674	present
Borrelia garinii PBi linear genome	28.3	904246	absent
Borrelia hermsii DAH	29.8	922307	absent
Borrelia recurrentis A1	27.5	930981	present
Borrelia turicatae	29.1	917330	absent
Brachybacterium faecium DSM 4810	72	3614992	absent
Brachyspira hyodysenteriae WA1	27.1	3000694	present
Brachyspira murdochii DSM 12563	27.8	3241804	absent
Brachyspira pilosicoli	27.9	2586443	absent
Bradyrhizobium	64.1	9105828	absent
Bradyrhizobium sp.	64.9	8264687	present
Bradyrhizobium sp.	65.5	7456587	absent
Brevibacillus brevis NBRC 100599	47.3	6296436	absent
Brevundimonas subvibrioides ATCC	68.4	3445263	absent
Brucella abortus bv. 1 str. 9-941	57.2	2124241	absent
Brucella abortus bv. 1 str. 9-941 chromosome	57.3	1162204	absent
Brucella abortus S19	57.2	2122487	absent
Brucella abortus S19 chromosome 2	57.3	1161449	absent
Brucella canis ATCC 23365 chromosome I	57.2	2105969	absent
Brucella canis ATCC 23365 chromosome II	57.3	1206800	absent
Brucella melitensis	57.2	2117144	absent
Brucella melitensis ATCC 23457	57.2	2125701	absent

Brucella melitensis ATCC 23457	57.3	1185518	absent
Brucella melitensis biovar Abortus 2308	57.2	2121359	absent
Brucella melitensis biovar Abortus 2308	57.3	1156948	absent
Brucella melitensis chromosome II	57.3	1177787	absent
Brucella microti CCM	57.2	2117050	absent
Brucella microti CCM 4915 chromosome 2	57.3	1220319	absent
Brucella ovis ATCC 25840 chromosome I	57.2	2111370	absent
Brucella ovis ATCC 25840 chromosome II	57.2	1164220	absent
Brucella suis 1330	57.2	2107794	absent
Brucella suis 1330 chromosome II	57.3	1207381	absent
Brucella suis ATCC	57.1	1923763	absent
Brucella suis ATCC 23445 chromosome II	57.3	1400844	absent
Buchnera aphidicola (Baizongia pistaciae)	25.3	615980	present
Buchnera aphidicola (Cinara tujafilina)	23	444925	absent
Buchnera aphidicola str. 5A (Acyrthosiphon	26.3	642122	absent
Buchnera aphidicola str. Cc (Cinara cedri)	20.1	416380	absent
Buchnera aphidicola str. Sg (Schizaphis	25.3	641454	absent
Buchnera aphidicola str. Tuc7 (Acyrthosiphon	26.3	641895	absent
Buchnera sp. APS	26.3	640681	present
Burkholderia ambifaria MC40-6 chromosome 1	66.9	3443583	present
Burkholderia ambifaria MC40-6 chromosome 2	66.5	2769414	present
Burkholderia ambifaria MC40-6 chromosome 3	65.9	1127947	present
Burkholderia cenocepacia AU 1054	66.9	3294563	absent
Burkholderia cenocepacia AU 1054	66.9	2788459	absent
Burkholderia cenocepacia AU 1054	67	1196094	absent
Burkholderia cenocepacia HI2424	66.8	3483902	present
Burkholderia cenocepacia HI2424	66.9	2998664	present
Burkholderia cenocepacia HI2424	67.3	1055417	present
Burkholderia cenocepacia J2315	66.7	3870082	present

Burkholderia cenocepacia J2315	67.3	3217062	present
Burkholderia cenocepacia J2315	66.9	875977	present
Burkholderia cenocepacia MC0-3	66.7	3532883	absent
Burkholderia cenocepacia MC0-3	66.6	3213911	absent
Burkholderia cenocepacia MC0-3	66.2	1224595	absent
Burkholderia cepacia AMMD chromosome 1	66.9	3556545	present
Burkholderia cepacia AMMD chromosome 2	66.8	2646969	present
Burkholderia cepacia AMMD chromosome 3	66.5	1281472	present
Burkholderia gladioli	67.5	4413616	present
Burkholderia gladioli BSR3 chromosome 2	68.6	3700833	present
Burkholderia glumae	68.1	3906529	present
Burkholderia glumae BGR1 chromosome 2	68.8	2827355	present
Burkholderia mallei ATCC 23344	68.2	3510148	absent
Burkholderia mallei ATCC 23344	69	2325379	absent
Burkholderia mallei NCTC 10229	68.9	2284095	absent
Burkholderia mallei NCTC 10229	68.2	3458208	absent
Burkholderia mallei NCTC 10247	69	2352693	absent
Burkholderia mallei NCTC 10247	68.2	3495687	absent
Burkholderia mallei	68.9	1734922	absent
Burkholderia mallei SAVP1 chromosome II	68.1	3497479	absent
Burkholderia multivorans ATCC	66.9	3448466	present
Burkholderia multivorans ATCC 17616 JGI chromosome	67.1	2472928	present
Burkholderia multivorans ATCC 17616 JGI chromosome	65.8	919806	present
Burkholderia multivorans ATCC	66.9	3448421	present
Burkholderia multivorans ATCC 17616 Tohoku	67.1	2473162	present
Burkholderia multivorans ATCC 17616 Tohoku	65.8	919805	present

Burkholderia phymatum STM815	63	3479187	present
Burkholderia phymatum STM815 chromosome 2	62.3	2697374	present
Burkholderia phytofirmans PsJN	62.6	4467537	present
Burkholderia phytofirmans PsJN	62.1	3625999	present
Burkholderia pseudomallei 1106a	68	3988455	absent
Burkholderia pseudomallei 1106a	68.6	3100794	absent
Burkholderia pseudomallei 1710b	67.6	4126292	absent
Burkholderia	68	3912947	absent
Burkholderia pseudomallei 668	68.6	3127456	absent
Burkholderia pseudomallei K96243	67.7	4074542	absent
Burkholderia pseudomallei K96243	68.5	3173005	absent
Burkholderia pseudomallei	67.7	4098576	absent
Burkholderia rhizoxinica HKI 454	61.2	2755309	absent
Burkholderia rhizoxinica HKI 454	59.7	822304	absent
Burkholderia sp. 383 chromosome 1	66.2	3694126	absent
Burkholderia sp. 383 chromosome 2	66.7	3587082	absent
Burkholderia sp. 383 chromosome 3	65.3	1395069	absent
Burkholderia sp.	63.7	4063449	absent
Burkholderia sp. CCGE1001	63.5	2770302	absent
Burkholderia sp.	64.1	3518940	present
Burkholderia sp. CCGE1002	63.2	2593966	present
Burkholderia sp. CCGE1002	62.6	1282816	present
Burkholderia sp.	63.4	4077097	absent
Burkholderia sp. CCGE1003	63	2966498	absent
Burkholderia thailandensis E264	67.3	3809201	absent
Burkholderia thailandensis E264	68.1	2914771	absent
Burkholderia	66.5	3652814	present
Burkholderia vietnamiensis G4	66.8	2411759	present
Burkholderia vietnamiensis G4	66.4	1241007	present

Burkholderia xenovorans LB400	62.8	4895836	absent
Burkholderia xenovorans LB400	62.8	3363523	absent
Burkholderia_pseudomallei_1710b	68.5	3181762	absent
Butyrivibrio proteoclasticus B316	40.2	3554804	present
Butyrivibrio proteoclasticus B316	40	302358	present
Caldicellulosiruptor hydrothermalis 108	36.1	2770676	absent
Caldicellulosiruptor kristjanssonii 177R1B	36.1	2786473	present
Caldicellulosiruptor kronotskyensis 2002	35.1	2843785	absent
Caldicellulosiruptor obsidiansis OB47	35.2	2532343	absent
Caldicellulosiruptor owensensis OL	35.4	2428903	absent
Caldicellulosiruptor saccharolyticus DSM	35.3	2970275	absent
Calditerrivibrio nitroreducens DSM	35.8	2157835	present
Campylobacter concisus	39.4	2052007	present
Campylobacter curvus	44.5	1971264	absent
Campylobacter fetus subsp. fetus 82-40	33.3	1773615	absent
Campylobacter hominis ATCC BAA-381	31.7	1711273	present
Campylobacter jejuni	30.3	1777831	absent
Campylobacter jejuni subsp. doylei 269.97	30.6	1845106	absent
Campylobacter jejuni subsp. jejuni 81116	30.5	1628115	absent
Campylobacter jejuni subsp. jejuni 81-176	30.6	1616554	present
Campylobacter jejuni subsp. jejuni	30.6	1664840	present
Campylobacter jejuni subsp. jejuni NCTC	30.5	1641481	absent
Campylobacter lari	29.7	1525460	present
Candidatus Accumulibacter	64.1	5058518	present
Candidatus Amoebophilus asiaticus	35	1884364	absent
Candidatus Azobacteroides pseudotrichonymphae	32.7	1114206	present
Candidatus Blochmannia vafer str.	27.5	722593	absent
Candidatus Carsonella ruddii PV	16.6	159662	absent

Candidatus Desulfococcus	56.2	3944167	absent
Candidatus Desulforudis	60.8	2349476	absent
Candidatus Hamiltonella defensa 5AT (Acyrthosiphon	40.3	2110331	present
Candidatus Hodgkinia cicadicola Dsem	58.4	143795	absent
Candidatus Koribacter versatilis Ellin345	58.4	5650368	absent
Candidatus Liberibacter asiaticus str. psy62	36.5	1226704	absent
Candidatus Liberibacter solanacearum CLso-	35.2	1258278	absent
Candidatus Nitrospira	59	4317083	absent
Candidatus Pelagibacter sp. IMCC9063	31.7	1284727	absent
Candidatus Phytoplasma	27.4	879959	absent
Candidatus	21.4	601943	absent
Candidatus Riesia pediculicola USDA	28.5	574390	present
Candidatus Ruthia magnifica str. Cm (Calyptogena	34	1160782	absent
Candidatus Sulcia muelleri CARI	21.1	276511	absent
Candidatus Sulcia muelleri DMIN	22.5	243933	absent
Candidatus Sulcia muelleri GWSS	22.4	245530	absent
Candidatus Sulcia muelleri SMDSEM	22.6	276984	absent
Candidatus Vesicomyosocius	31.6	1022154	absent
Candidatus Zinderia insecticola CARI	13.5	208564	absent
Capnocytophaga ochracea DSM 7271	39.6	2612925	absent
Carboxydothermus hydrogenoformans Z-	42	2401520	absent
Carnobacterium sp. 17-	35.3	2635294	present
Catenulispora acidiphila DSM 44928	69.8	10467782	absent
Caulobacter crescentus	67.2	4016947	absent
Caulobacter crescentus NA1000	67.2	4042929	absent
Caulobacter segnis ATCC 21756	67.7	4655622	absent
Caulobacter sp. K31	67.5	5477872	present
Cellulomonas fimi	74.7	4266344	absent
Cellulomonas flavigena DSM 20109	74.3	4123179	absent

Cellulophaga algicola DSM 14237	33.8	4888353	absent
Cellulophaga lytica	32.1	3765936	absent
Cellvibrio japonicus	52	4576573	absent
Chitinophaga pinensis DSM 2588	45.2	9127347	absent
Chlamydia muridarum	40.3	1072950	present
Chlamydia trachomatis	41.3	1038842	absent
Chlamydia trachomatis A/HAR-13	41.3	1044459	absent
Chlamydia trachomatis B/TZ1A828/OT	41.3	1044282	absent
Chlamydia trachomatis D/UW-3/CX	41.3	1042519	absent
Chlamydia trachomatis	41.3	1044352	absent
Chlamydia trachomatis L2b/UCH-1/proctitis	41.3	1038869	absent
Chlamydophila abortus	39.9	1144377	absent
Chlamydophila caviae	39.2	1173390	present
Chlamydophila felis	39.4	1166239	present
Chlamydophila	41.1	1106197	absent
Chlamydophila pneumoniae AR39	40.6	1229853	absent
Chlamydophila pneumoniae CWL029	40.6	1230230	absent
Chlamydophila pneumoniae J138	40.6	1226565	absent
Chlamydophila pneumonie TW-183	40.6	1225935	absent
Chlamydophila psittaci	39.1	1171660	present
Chlorobaculum parvum NCIB 8327	55.8	2289249	absent
Chlorobium chlorochromatii CaD3	44.3	2572079	absent
Chlorobium limicola	51.3	2763181	absent
Chlorobium phaeobacteroides BS1	48.9	2736403	absent
Chlorobium phaeobacteroides DSM	48.4	3133902	absent
Chlorobium tepidum	56.5	2154946	absent
Chloroflexus aggregans DSM 9485	56.4	4684931	absent
Chloroflexus aurantiacus J-10-fl	56.7	5258541	absent
Chloroflexus sp. Y-400-	56.7	5268950	absent
Chloroherpeton thalassium ATCC	45	3293456	absent
Chromobacterium violaceum ATCC	64.8	4751080	absent
Chromohalobacter salexigens DSM 3043	63.9	3696649	absent
Citrobacter koseri ATCC BAA-895	53.8	4720462	present
Citrobacter rodentium	54.7	5346659	present

Clavibacter michiganensis subsp. michiganensis NCPPB	72.7	3297891	present
Clavibacter michiganensis subsp.	72.6	3258645	absent
Clostridiales genomosp. BVAB3 str. UPII9-5	44.2	1810248	absent
Clostridium acetobutylicum	30.9	3940880	present
Clostridium beijerinckii NCIMB 8052	29.9	6000632	absent
Clostridium botulinum A str. ATCC 19397	28.2	3863450	absent
Clostridium botulinum A str. ATCC 3502	28.2	3886916	present
Clostridium botulinum A str. Hall	28.2	3760560	absent
Clostridium botulinum A2 str. Kyoto	28.2	4155278	absent
Clostridium botulinum A3 str. Loch Maree	28.3	3992906	present
Clostridium botulinum B str. Eklund 17B	27.5	3800327	present
Clostridium botulinum B1 str. Okra	28.3	3958233	present
Clostridium botulinum Ba4 str. 657	28.2	3977794	present
Clostridium botulinum BKT015925	28.5	2773157	present
Clostridium botulinum E3 str. Alaska E43	27.4	3659644	absent
Clostridium botulinum F str. Langeland	28.3	3995387	present
Clostridium	37.4	4068724	absent
Clostridium	31.2	5262222	absent
Clostridium difficile	29.1	4290252	present
Clostridium difficile	28.6	4110554	absent
Clostridium difficile	28.8	4191339	absent
Clostridium kluyveri	32	3964618	present
Clostridium kluyveri NBRC 12016	32	3896121	present
Clostridium lentocellum DSM 5427	34.3	4714237	absent
Clostridium ljungdahlii ATCC 49587	31.1	4630065	absent
Clostridium novyi NT	28.9	2547720	absent
Clostridium perfringens	28.6	3031430	present
Clostridium perfringens ATCC 13124	28.4	3256683	absent
Clostridium perfringens	28.2	2897393	present
Clostridium phytofermentans ISDg	35.3	4847594	absent
Clostridium saccharolyticum WM1	45	4662871	absent

Clostridium sticklandii DSM 519	33.3	2715461	absent
Clostridium tetani E88	28.7	2799251	absent
Clostridium thermocellum ATCC	39	3843301	absent
Colwellia	38	5373180	absent
Comamonas testosteroni CNB-2	61.4	5373643	absent
Conexibacter woesei DSM 14684	72.7	6359369	absent
Coprothermobacter proteolyticus DSM	44.8	1424912	absent
Coraliomargarita akajimensis DSM	53.6	3750771	absent
Coriobacterium glomerans PW2	60.4	2115681	absent
Corynebacterium aurimucosum ATCC	60.6	2790189	present
Corynebacterium	53.5	2488635	absent
Corynebacterium efficiens YS-314	63.1	3147090	absent
Corynebacterium glutamicum ATCC	53.8	3282708	absent
Corynebacterium glutamicum ATCC	53.8	3309401	absent
Corynebacterium glutamicum R	54.1	3314179	present
Corynebacterium jeikeium K411	61.4	2462499	absent
Corynebacterium kroppenstedtii DSM	57.5	2446804	absent
Corynebacterium pseudotuberculosis	52.2	2337913	absent
Corynebacterium urealyticum DSM 7109	64.2	2369219	absent
Coxiella burnetii	42.7	1995281	absent
Coxiella burnetii	42.6	2008870	absent
Coxiella burnetii	42.7	2063100	present
Coxiella burnetii Dugway 7E9-12	42.4	2158758	present
Coxiella burnetii RSA	42.8	2016427	present
Croceibacter atlanticus HTCC2559	33.9	2952962	absent
Cronobacter turicensis	57.4	4384526	present
Cryptobacterium curtum DSM 15641	50.9	1617804	absent
Cupriavidus	67.5	3416911	present
Cupriavidus taiwanensis	67.9	2502411	present
Cyanobacterium	31.1	1443806	absent
Cyanothece sp. ATCC 51142 chromosome	37.9	4934271	present
Cyanothece sp. ATCC 51142 chromosome	38.6	429701	present

Cyanothece sp. PCC	38.6	5942652	present
Cyanothece sp. PCC	50.8	5374574	present
Cyanothece sp. PCC	40.2	6091620	present
Cyanothece sp. PCC	39.8	4679413	present
Cyanothece sp. PCC	39.8	4669813	present
Cytophaga hutchinsonii ATCC 33406	38.8	4433218	absent
Dechloromonas aromatica RCB	59.2	4501104	absent
Deferribacter desulfuricans SSM1	31.1	2234389	present
Dehalococcoides ethenogenes 195	48.9	1469720	absent
Dehalococcoides sp.	47.2	1341892	absent
Dehalococcoides sp.	47	1395502	absent
Dehalococcoides sp.	47.3	1360154	absent
Dehalococcoides sp. VS	47.3	1413462	absent
Dehalogenimonas lykanthroporepellens	55	1686510	absent
Deinococcus deserti	63.4	2819842	present
Deinococcus geothermalis DSM	66.6	2467205	absent
Deinococcus maricopensis DSM	69.8	3498530	absent
Deinococcus proteolyticus MRP	66.2	2147060	present
Deinococcus	67	2648638	present
Deinococcus radiodurans R1	66.7	412348	present
Delftia acidovorans	66.5	6767514	absent
Delftia sp. Cs1-4	66.7	6685842	absent
Denitrovibrio acetiphilus DSM 12809	42.5	3222077	absent
Desulfarculus baarsii DSM 2075	65.7	3655731	absent
Desulfatibacillum alkenivorans AK-01	54.5	6517073	absent
Desulfitobacterium hafniense DCB-2	47.5	5279134	absent
Desulfitobacterium hafniense Y51	47.4	5727534	absent
Desulfobacca acetoxidans DSM	52.9	3282536	absent
Desulfobacterium autotrophicum HRM2	48.8	5589073	present
Desulfobulbus propionicus DSM 2032	58.9	3851869	absent
Desulfohalobium retbaense DSM 5692	57.5	2864304	present
Desulfomicrobium baculatum DSM 4028	58.6	3942657	absent
Desulfotalea psychrophila LSv54	46.8	3523383	present

Desulfotomaculum acetoxidans DSM 771	41.6	4545624	absent
Desulfotomaculum carboxydivorans CO-1-	46.6	2892255	absent
Desulfotomaculum kuznetsovii DSM 6115	54.9	3601386	absent
Desulfotomaculum reducens MI-1	42.3	3608104	absent
Desulfotomaculum ruminis DSM 2154	47.2	3969014	absent
Desulfovibrio aespoeensis Aspo-2	62.6	3629109	absent
Desulfovibrio desulfuricans G20	57.8	3730232	absent
Desulfovibrio desulfuricans subsp. desulfuricans str. ATCC	58.1	2873437	absent
Desulfovibrio	62.8	5248049	present
Desulfovibrio salexigens DSM 2638	47.1	4289847	absent
Desulfovibrio vulgaris str. Miyazaki F	67.1	4040304	absent
Desulfovibrio vulgaris subsp. vulgaris DP4	63	3462887	present
Desulfovibrio vulgaris subsp. vulgaris str.	63.1	3570858	present
Desulfurispirillum	56.1	2928377	absent
Desulfurivibrio alkaliphilus AHT2	60.3	3097763	absent
Desulfurobacterium thermolithotrophum DSM 11699	34.9	1541968	absent
Diaphorobacter sp.	66.8	3796573	absent
Dichelobacter nodosus VCS1703A	44.4	1389350	absent
Dickeya dadantii 3937	56.3	4922802	absent
Dickeya dadantii	53.6	4818394	absent
Dickeya dadantii	55	4679450	absent
Dickeya zeae Ech1591	54.5	4813854	absent
Dictyoglomus thermophilum H-6-12	33.7	1959987	absent
Dictyoglomus turgidum DSM 6724	34	1855560	absent
Dinoroseobacter shibae DFL 12	66	3789584	present
Dyadobacter fermentans DSM 18053	51.5	6967790	absent
Edwardsiella ictaluri	57.4	3812315	absent
Edwardsiella tarda	59.7	3760463	present
Eggerthella lenta DSM	64.2	3632260	absent
Ehrlichia canis str. Jake	29	1315030	absent
Ehrlichia chaffeensis str. Arkansas	30.1	1176248	absent

Ehrlichia ruminantium str. Gardel	27.5	1499920	absent
Ehrlichia ruminantium str. Welgevonden	27.5	1512977	absent
Ehrlichia ruminantium str. Welgevonden	27.5	1516355	absent
Elusimicrobium minutum Pei191	40	1643562	absent
Enterobacter aerogenes KCTC 2190	54.8	5280350	absent
Enterobacter cloacae	57	4814049	absent
Enterobacter cloacae subsp. cloacae ATCC	54.8	5314581	present
Enterobacter sakazakii ATCC BAA-894	56.8	4368373	present
Enterobacter sp. 638	53	4518712	present
Enterococcus faecalis	37.5	3218031	absent
Erwinia amylovora	53.6	3805573	absent
Erwinia amylovora ATCC 49946	53.6	3805874	present
Erwinia billingiae	55.2	5100167	present
Erwinia carotovora subsp. atroseptica	51	5064019	absent
Erwinia pyrifoliae	53.4	4026322	present
Erwinia tasmaniensis	53.7	3883467	present
Erysipelothrix rhusiopathiae str.	36.6	1787941	absent
Erythrobacter litoralis HTCC2594	63.1	3052398	absent
Escherichia coli 0127:H6 E2348/69	50.6	4965553	present
Escherichia coli 536	50.5	4938920	absent
Escherichia coli 55989	50.7	5154862	absent
Escherichia coli APEC	50.5	5082025	absent
Escherichia coli ATCC	50.9	4746218	absent
Escherichia coli B str.	50.8	4629812	absent
Escherichia coli	50.8	4570938	absent
Escherichia coli	50.8	4578159	absent
Escherichia coli	50.5	5231428	absent
Escherichia coli	50.6	4979619	present
Escherichia coli ED1a	50.7	5209548	absent
Escherichia coli HS	50.8	4643538	absent
Escherichia coli IAI1	50.8	4700560	absent
Escherichia coli IAI39	50.6	5132068	absent
Escherichia coli K-12 substr. W3110	50.8	4646332	absent
Escherichia coli O103:H2 str. 12009	50.7	5449314	present
Escherichia coli O111:H- str. 11128	50.6	5371077	present
Escherichia coli	50.5	5498450	present
Escherichia coli O157:H7 EDL933	50.4	5528445	absent

Escherichia coli O157:H7 str. EC4115	50.5	5572075	present
Escherichia coli O157:H7 str. TW14359	50.5	5528136	present
Escherichia coli O26:H11 str. 11368	50.7	5697240	present
Escherichia coli O55:H7 str. CB9615	50.5	5386352	present
Escherichia coli S88	50.7	5032268	absent
Escherichia coli SE11	50.8	4887515	present
Escherichia coli SMS-	50.5	5068389	present
Escherichia coli str. K-12 substr. DH10B	50.8	4686137	absent
Escherichia coli str. K-12 substr. MG1655	50.8	4639675	absent
Escherichia coli	50.7	5202090	absent
Escherichia coli UTI89	50.6	5065741	present
Escherichia fergusonii ATCC 35469	49.9	4588711	absent
Ethanoligenens harbinense YUAN-3	55.6	3008576	absent
Eubacterium eligens ATCC 27750	37.7	2144190	present
Eubacterium limosum	47.5	4276902	absent
Eubacterium rectale ATCC 33656	41.5	3449685	absent
Exiguobacterium sibiricum 255-15	47.7	3034136	present
Exiguobacterium sp.	48.5	2999895	absent
Ferrimonas balearica DSM 9799	60.2	4279159	absent
Fervidobacterium nodosum Rt17-B1	35	1948941	absent
Fibrobacter succinogenes subsp.	48.1	3842635	absent
Finegoldia magna	32.3	1797577	present
Flavobacteriaceae bacterium 3519-10	42.7	2768102	absent
Flavobacteriales bacterium HTCC2170	37	3868304	absent
Flavobacterium johnsoniae UW101	34.1	6096872	absent
Flavobacterium psychrophilum JIP02/86	32.5	2861988	absent
Fluviicola taffensis	36.5	4633577	absent
Francisella philomiragia subsp. philomiragia	32.6	2045775	present
Francisella tularensis subsp. holarctica	32.2	1895994	absent
Francisella tularensis subsp. holarctica	32.2	1890909	absent
Francisella tularensis subsp. holarctica	32.2	1895727	absent

Francisella tularensis subsp. mediasiatica	32.3	1893886	absent
Francisella tularensis subsp. novicida U112	32.5	1910031	absent
Francisella tularensis subsp. tularensis FSC	32.3	1892616	absent
Francisella tularensis subsp. tularensis Schu4	32.3	1892775	absent
Francisella tularensis subsp. tularensis	32.3	1898476	absent
Frankia alni ACN14a	72.8	7497934	absent
Frankia sp. CcI3	70.1	5433628	absent
Frankia sp. EAN1pec	71.2	8982042	absent
Frankia sp. EuI1c	72.3	8815781	absent
Frankia symbiont of Datisca glomerata	70	5323186	present
Fusobacterium nucleatum subsp.	27.2	2174500	absent
Gallibacterium anatis	39.9	2687335	present
Gallionella capsiferriformans ES-2	52.8	3162471	absent
Gamma	53.3	4587455	absent
Gardnerella vaginalis	42	1617545	absent
Gardnerella vaginalis ATCC 14019	41.4	1667350	absent
Gemmatimonas aurantiaca T-27	64.3	4636964	absent
Geobacillus kaustophilus HTA426	52.1	3544776	present
Geobacillus sp. C56-T3	52.5	3650813	absent
Geobacillus sp. WCH70	42.8	3464618	present
Geobacillus sp.	44	3840330	present
Geobacillus sp.	51.2	7121665	absent
Geobacillus sp.	52.4	3628883	present
Geobacillus sp.	52.4	3622844	present
Geobacillus thermodenitrificans	49	3550319	present
Geobacter bemidjiensis	60.3	4615150	absent
Geobacter lovleyi SZ	54.8	3917761	present
Geobacter metallireducens GS-15	59.5	3997420	present
Geobacter sp. FRC-32	53.5	4304501	absent
Geobacter sp. M18	61.2	5277406	absent
Geobacter sp. M21	60.5	4745806	absent
Geobacter	60.9	3814139	absent
Geobacter uraniumreducens Rf4	54.2	5136364	absent
Geodermatophilus obscurus DSM 43160	74	5322497	absent
Glaciecola sp. 4H-3-	44.2	5052309	present
Gloeobacter violaceus	62	4659019	absent
Gluconacetobacter diazotrophicus PAl 5	66.4	3944163	present

Gluconacetobacter diazotrophicus PAl 5	66.4	3887492	present
Gluconobacter oxydans	61.1	2702173	present
Gordonia bronchialis DSM 43247	67.1	5208602	present
Gramella forsetii	36.6	3798465	absent
Granulobacter bethesdensis CGDNIH1	59.1	2708355	absent
Haemophilus ducreyi	38.2	1698955	absent
Haemophilus influenzae 86-028NP	38.2	1914490	absent
Haemophilus influenzae	38.2	1985832	absent
Haemophilus influenzae	38.2	2007018	absent
Haemophilus influenzae	38	1813033	absent
Haemophilus influenzae	38	1887192	absent
Haemophilus influenzae	38.2	1830138	absent
Haemophilus parasuis	40	2269156	absent
Haemophilus somnus	37.2	2007700	present
Haemophilus somnus	37.4	2263857	absent
Hahella chejuensis	53.9	7215267	absent
Halanaerobium sp.	33.2	2613117	absent
Haliangium ochraceum DSM 14365	69.5	9446314	absent
Haliscomenobacter hydrossis DSM 1100	47.1	8371686	present
Halomonas elongata DSM 2581	63.6	4061296	absent
Halorhodospira	68	2678452	absent
Halothermothrix orenii	37.9	2578146	absent
Halothiobacillus neapolitanus c2	54.7	2582886	absent
Helicobacter acinonychis str. Sheeba	38.2	1553927	present
Helicobacter felis ATCC 49179	44.5	1654761	absent
Helicobacter hepaticus ATCC 51449	35.9	1799146	absent
Helicobacter mustelae	42.5	1578097	absent
Helicobacter pylori	38.9	1667867	absent
Helicobacter pylori B38	39.2	1576758	absent
Helicobacter pylori B8	38.8	1673997	present
Helicobacter pylori G27	38.9	1652982	present
Helicobacter pylori	39.1	1596366	present
Helicobacter pylori P12	38.8	1673813	present
Helicobacter pylori	38.9	1629557	present
Helicobacter pylori	38.9	1608548	absent
Helicobacter pylori	38.9	1658051	absent
Helicobacter pylori,	39.2	1643831	absent
Heliobacterium modesticaldum Ice1	57	3075407	absent
Herbaspirillum seropedicae SmR1	63.4	5513887	absent
Herminiimonas	54.3	3424307	absent

Herpetosiphon aurantiacus ATCC	50.7	6346587	present
Hippea maritima DSM	37.5	1694430	absent
Hirschia baltica ATCC	45.2	3455622	present
Hydrogenobacter thermophilus TK-6	44	1743135	absent
Hydrogenobaculum sp. Y04AAS1	34.8	1559514	absent
Hyphomicrobium denitrificans ATCC	60.8	3638969	absent
Hyphomonas neptunium ATCC	61.9	3705021	absent
Idiomarina loihiensis	47	2839318	absent
Ilyobacter polytropus DSM 2926	34.5	2046464	present
Intrasporangium calvum DSM 43043	70.7	4024382	absent
Isoptericola variabilis	73.9	3307740	absent
Isosphaera pallida ATCC 43644	62.4	5472964	present
Jannaschia sp. CCS1	62.3	4317977	present
Janthinobacterium sp.	54.2	4110251	absent
Jonesia denitrificans DSM 20603	58.4	2749646	absent
Kangiella koreensis DSM 16069	43.7	2852073	absent
Ketogulonicigenium vulgare Y25	61.7	2776084	present
Kineococcus radiotolerans SRS30216	74.4	4761183	present
Klebsiella pneumoniae	57.3	5641239	present
Klebsiella pneumoniae NTUH-K2044	57.7	5248520	absent
Klebsiella pneumoniae subsp. pneumoniae	57.5	5315120	present
Klebsiella variicola At-	57.6	5458505	absent
Kocuria rhizophila	71.2	2697540	absent
Kosmotoga olearia TBF	41.5	2302126	absent
Kribbella flavida DSM	70.6	7579488	absent
Krokinobacter sp. 4H-3-	37.3	3389993	absent
Kytococcus sedentarius DSM 20547	71.6	2785024	absent
Lacinutrix sp. 5H-3-7-4	30.8	3296168	absent
Lactobacillus	38.1	2078001	present
Lactobacillus acidophilus NCFM	34.7	1993560	absent
Lactobacillus amylovorus GRL 1112	38.2	2067702	absent
Lactobacillus brevis	46.2	2291220	present
Lactobacillus buchneri NRRL B-30929	44.4	2506301	present
Lactobacillus casei	46.6	2895264	present
Lactobacillus casei	46.3	3079196	absent
Lactobacillus casei str.	46.5	2861848	absent

Lactobacillus crispatus	36.9	2043161	absent
Lactobacillus delbrueckii subsp.	49.7	1864998	absent
Lactobacillus delbrueckii subsp.	49.7	1856951	absent
Lactobacillus delbrueckii subsp.	49.6	2125753	present
Lactobacillus fermentum IFO 3956	51.5	2098685	absent
Lactobacillus gasseri ATCC 33323	35.3	1894360	absent
Lactobacillus helveticus DPC 4571	37.1	2080931	absent
Lactobacillus johnsonii	34.5	1755993	present
Lactobacillus johnsonii NCC 533	34.6	1992676	absent
Lactobacillus kefiranofaciens ZW3	37.7	2113023	present
Lactobacillus plantarum	44.5	3308274	absent
Lactobacillus plantarum	44.7	3197759	absent
Lactobacillus plantarum subsp. plantarum ST-III	44.6	3254376	present
Lactobacillus reuteri DSM 20016	38.9	1999618	absent
Lactobacillus reuteri F275 Kitasato	38.9	2039414	absent
Lactobacillus	46.7	3010111	absent
Lactobacillus rhamnosus Lc 705	46.7	2968598	present
Lactobacillus sakei subsp. sakei 23K	41.3	1884661	absent
Lactobacillus salivarius subsp. salivarius	32.9	1827111	present
Lactococcus lactis subsp. cremoris	35.7	2529478	absent
Lactococcus lactis subsp. cremoris SK11	35.9	2438589	present
Lactococcus lactis	35.3	2365589	absent
Lactococcus lactis subsp. lactis KF147	34.9	2598144	present
Laribacter hongkongensis HLHK9	62.4	3169329	absent
Lawsonia intracellularis PHE/MN1-00	33.3	1457619	present
Leadbetterella byssophila DSM 17132	40.4	4059653	absent
Legionella longbeachae NSW150	37.1	4077332	absent
Legionella pneumophila 2300/99 Alcoy	38.4	3516334	absent
Legionella pneumophila str. Corby	38.5	3576470	absent
Legionella pneumophila str. Lens	38.4	3345687	present

Legionella pneumophila str. Paris	38.4	3503610	present
Legionella pneumophila subsp. pneumophila str.	38.3	3397754	absent
Leifsonia xyli subsp. xyli str. CTCB07	67.7	2584158	absent
Leptospira biflexa serovar Patoc strain	38.9	3603977	present
Leptospira biflexa serovar Patoc strain Patoc 1 (Ames)	39.3	277995	present
Leptospira biflexa serovar Patoc strain	38.9	3599677	present
Leptospira biflexa serovar Patoc strain Patoc 1 (Paris)	39.3	277655	present
Leptospira borgpetersenii serovar	40.2	3576473	absent
Leptospira borgpetersenii serovar Hardjo-bovis JB197	40.4	299762	absent
Leptospira borgpetersenii serovar	40.2	3614446	absent
Leptospira borgpetersenii serovar Hardjo-bovis L550	40.2	317336	absent
Leptospira interrogans serovar Copenhageni str. Fiocruz L1-130	35	4277185	absent
Leptospira interrogans serovar Copenhageni str. Fiocruz L1-130	35	350181	absent
Leptospira interrogans serovar lai str. 56601	35	4332241	absent
Leptospira interrogans serovar lai str. 56601	35.1	358943	absent
Leptothrix cholodnii	68.9	4909403	absent
Leptotrichia buccalis DSM 1135	29.6	2465610	absent
Leuconostoc citreum	39	1796284	present
Leuconostoc gasicomitatum LMG	36.7	1954080	absent
Leuconostoc kimchii IMSNU11154	38	2002721	present
Leuconostoc mesenteroides subsp. mesenteroides ATCC	37.7	2038396	present
Listeria innocua	37.4	3011208	present
Listeria monocytogenes 08-5578	38	3032288	absent
Listeria monocytogenes 08-5923	38	2999054	absent
Listeria monocytogenes Clip81459	38.1	2912690	absent

Listeria monocytogenes HCC23	38.2	2976212	absent
Listeria monocytogenes str. 4b F2365	38	2905187	absent
Listeria monocytogenes strain EGD	38	2944528	absent
Listeria seeligeri serovar 1/2b str.	37.4	2797636	absent
Listeria welshimeri serovar 6b str.	36.4	2814130	absent
Lysinibacillus	37.3	4639821	present
Macrococcus caseolyticus JCSC5402	36.9	2102324	present
Magnetococcus sp. MC-	54.2	4719581	absent
Magnetospirillum magneticum AMB-1	65.1	4967148	absent
Mahella australiensis 50-1 BON	43.5	3135972	absent
Mannheimia succiniciproducens	42.5	2314078	absent
Maricaulis maris	62.7	3368780	absent
Marinithermus hydrothermalis DSM	68.1	2269167	absent
Marinobacter aquaeolei	57.3	4326849	present
Marinomonas mediterranea MMB-1	44.1	4684316	absent
Marinomonas posidonica IVIA-Po-	44.3	3899940	absent
Marinomonas sp.	42.6	5100344	absent
Marivirga tractuosa	35.5	4511574	present
Meiothermus ruber	63.4	3097457	absent
Meiothermus silvanus DSM 9946	62.4	3249394	present
Melissococcus plutonius ATCC 35311	31.4	1891014	present
Mesoplasma florum L1	27	793224	absent
Mesorhizobium ciceri biovar biserrulae	62.7	6264489	present
Mesorhizobium loti MAFF303099	62.7	7036071	present
Mesorhizobium sp.	61.1	4412446	present
Methylacidiphilum infernorum V4	45.5	2287145	absent
Methylibium petroleiphilum PM1	69.2	4044195	present
Methylobacillus	55.7	2971517	absent
Methylobacterium chloromethanicum CM4	68.2	5777908	present
Methylobacterium extorquens AM1	68.7	5511322	present
Methylobacterium extorquens DM4	68.1	5943768	present
Methylobacterium extorquens PA1	68.2	5471154	absent

Methylobacterium nodulans ORS 2060	68.9	7772460	present
Methylobacterium	69.4	5800441	present
Methylobacterium radiotolerans JCM 2831	71.5	6077833	present
Methylobacterium sp.	71.6	7659055	present
Methylocella silvestris	63.1	4305430	absent
Methylococcus capsulatus str. Bath	63.6	3304561	absent
Methylomonas methanica MC09	51.3	5051681	absent
Methylotenera mobilis	45.5	2547570	absent
Methylotenera sp. 301	42.6	3059871	absent
Methylovorus sp.	55.4	2862391	absent
Methylovorus sp. SIP3-	54.9	2995511	present
Microbacterium testaceum StLB037	70.3	3982034	absent
Micrococcus luteus NCTC 2665	73	2501097	absent
Microcystis aeruginosa NIES-843	42.3	5842795	absent
Microlunatus phosphovorus NM-1	67.3	5683123	absent
Micromonospora aurantiaca ATCC 27029	72.8	7025559	absent
Micromonospora sp. L5	72.8	6962533	absent
Mobiluncus curtisii ATCC 43063	55.4	2146480	absent
Moorella thermoacetica ATCC 39073	55.8	2628784	absent
Moraxella catarrhalis	41.7	1863286	absent
Mycobacterium abscessus ATCC	64.1	5067172	absent
Mycobacterium avium	69	5475491	absent
Mycobacterium avium subsp. paratuberculosis	69.3	4829781	absent
Mycobacterium bovis BCG str. Pasteur	65.6	4374522	absent
Mycobacterium bovis BCG str. Tokyo 172	65.6	4371711	absent
Mycobacterium bovis subsp. bovis AF2122/97	65.6	4345492	absent
Mycobacterium gilvum PYR-GCK	67.9	5619607	present
Mycobacterium leprae	57.8	3268071	absent
Mycobacterium leprae strain TN	57.8	3268203	absent
Mycobacterium	65.7	6636827	present
Mycobacterium smegmatis str. MC2	67.4	6988209	absent
Mycobacterium sp.	68.4	4643668	absent
Mycobacterium sp. JLS	68.4	6048425	absent
Mycobacterium sp.	68.4	5737227	present
Mycobacterium sp.	68.5	5705448	present

Mycobacterium sp.	67.9	5547747	present
Mycobacterium tuberculosis CDC1551	65.6	4403837	absent
Mycobacterium tuberculosis F11	65.6	4424435	absent
Mycobacterium tuberculosis H37Ra	65.6	4419977	absent
Mycobacterium tuberculosis H37Rv	65.6	4411532	absent
Mycobacterium tuberculosis KZN 1435	65.6	4398250	absent
Mycobacterium ulcerans Agy99	65.5	5631606	absent
Mycobacterium vanbaalenii PYR-1	67.8	6491865	absent
Mycoplasma agalactiae	29.6	1006702	absent
Mycoplasma agalactiae	29.7	877438	absent
Mycoplasma arthritidis 158L3-1	30.7	820453	absent
Mycoplasma bovis	29.3	1003404	absent
Mycoplasma capricolum subsp.	23.8	1010023	absent
Mycoplasma	28.5	846214	absent
Mycoplasma crocodyli	27	934379	absent
Mycoplasma	26.9	977524	absent
Mycoplasma	26.9	1118751	absent
Mycoplasma	31.5	996422	absent
Mycoplasma genitalium	31.7	580076	absent
Mycoplasma haemofelis str. Langford 1	38.9	1147259	absent
Mycoplasma hominis	27.1	665445	absent
Mycoplasma hyopneumoniae 232	28.6	892758	absent
Mycoplasma hyopneumoniae 7448	28.5	920079	absent
Mycoplasma	28.5	897405	absent
Mycoplasma hyorhinis	25.9	839615	absent
Mycoplasma leachii	23.8	1008951	absent
Mycoplasma mobile	25	777079	absent
Mycoplasma mycoides subsp. mycoides SC	24	1211703	absent
Mycoplasma penetrans	25.7	1358633	absent
Mycoplasma	40	816394	absent
Mycoplasma pulmonis	26.6	963879	absent
Mycoplasma suis	31.1	709270	absent
Mycoplasma suis str.	31.1	742431	absent
Mycoplasma synoviae	28.5	799476	absent
Myxococcus xanthus	68.9	9139763	absent
Nakamurella multipartita DSM	70.9	6060298	absent
Natranaerobius thermophilus JW-NM-	36.3	3165557	present
Nautilia profundicola	33.5	1676444	absent

Neisseria gonorrhoeae FA 1090	52.7	2153922	absent
Neisseria gonorrhoeae NCCP11945	52.4	2232025	present
Neisseria lactamica ST-	52.3	2220606	absent
Neisseria meningitidis	51.7	2153416	absent
Neisseria meningitidis	51.9	2145295	absent
Neisseria meningitidis	51.6	2194961	absent
Neisseria meningitidis serogroup A strain	51.8	2184406	absent
Neisseria meningitidis serogroup B strain	51.5	2272360	absent
Neorickettsia risticii str.	41.3	879977	absent
Neorickettsia sennetsu str. Miyayama	41.1	859006	absent
Nitratifractor salsuginis DSM 16511	53.9	2101285	absent
Nitratiruptor sp. SB155-	39.7	1877931	absent
Nitrobacter	61.7	4406967	present
Nitrobacter winogradskyi Nb-255	62	3402093	absent
Nitrosococcus	51.6	4079427	present
Nitrosococcus oceani ATCC 19707	50.3	3481691	present
Nitrosococcus watsoni	50.1	3328570	present
Nitrosomonas europaea ATCC 19718	50.7	2812094	absent
Nitrosomonas eutropha	48.5	2661057	present
Nitrosomonas sp.	44.8	3180526	present
Nitrosospira multiformis ATCC	53.9	3184243	present
Nocardia farcinica	70.8	6021225	present
Nocardioides sp. JS614	71.7	4985871	present
Nocardiopsis dassonvillei subsp.	72.8	5767958	present
'Nostoc azollae' 0708	38.4	5354700	present
Nostoc punctiforme PCC 73102	41.4	8234322	present
Nostoc sp. PCC 7120	41.3	6413771	present
Novosphingobium aromaticivorans DSM	65.2	3561584	absent
Novosphingobium sp.	63.7	3911486	present
Oceanithermus profundus DSM 14977	70	2303940	present
Oceanobacillus	35.7	3630528	absent
Ochrobactrum anthropi ATCC 49188	56.1	2887297	present
Ochrobactrum anthropi ATCC 49188	56.2	1895911	present
Odoribacter splanchnicus DSM	43.4	4392288	absent
Oenococcus oeni PSU-1	37.9	1780517	absent
Oligotropha carboxidovorans OM5	62.4	3745629	absent

Olsenella uli DSM 7084	64.7	2051896	absent
Onion yellows	27.8	853092	absent
Opitutus terrae PB90-1	65.3	5957605	absent
Orientia tsutsugamushi Boryong	30.5	2127051	absent
Orientia tsutsugamushi str. Ikeda	30.5	2008987	absent
Paenibacillus polymyxa	45.8	5394884	absent
Paenibacillus polymyxa	45.2	5731816	present
Paenibacillus sp. JDR-2	50.3	7184930	absent
Paludibacter propionicigenes WB4	38.9	3685504	absent
Pantoea ananatis LMG	53.7	4690298	absent
Pantoea sp. At-9b	54.8	4368708	present
Pantoea vagans C9-1	55.5	4024986	present
Parabacteroides distasonis ATCC 8503	45.1	4811379	absent
Parachlamydia sp.	34.7	2414465	absent
Paracoccus denitrificans PD1222	66.7	2852282	present
Paracoccus denitrificans PD1222 chromosome 2	66.8	1730097	present
Parvibaculum lavamentivorans DS-1	62.3	3914745	absent
Parvularcula bermudensis	60.7	2902643	absent
Pasteurella multocida	40.4	2257487	absent
Pectobacterium carotovorum subsp.	51.9	4862913	absent
Pectobacterium wasabiae WPP163	50.5	5063892	absent
Pediococcus pentosaceus ATCC	37.4	1832387	absent
Pedobacter heparinus DSM 2366	42	5167383	absent
Pedobacter saltans DSM 12145	36.6	4635236	absent
Pelagibacter ubique HTCC1062	29.7	1308759	absent
Pelobacter carbinolicus DSM 2380	55.1	3665893	absent
Pelobacter propionicus DSM 2379	59	4008000	present
Pelodictyon luteolum	57.3	2364842	absent
Pelodictyon phaeoclathratiforme	48.1	3018238	absent
Pelotomaculum thermopropionicum SI	53	3025375	absent
Persephonella marina	37.2	1930284	present
Petrotoga mobilis SJ95	34.1	2169548	absent
Phenylobacterium zucineum HLK1	71.3	3996255	present
Photobacterium profundum SS9	42	4085304	present

Photobacterium profundum SS9	41.2	2237943	present
Photorhabdus	42.2	5064808	present
Photorhabdus	42.8	5688987	absent
Pirellula sp.	55.4	7145576	absent
Pirellula staleyi DSM	57.5	6196199	absent
Planctomyces brasiliensis DSM 5305	56.4	6006602	absent
Planctomyces limnophilus DSM 3776	53.7	5423075	present
Polaromonas naphthalenivorans CJ2	62.5	4410291	present
Polaromonas sp. JS666	62.5	5200264	present
Polymorphum gilvum SL003B-26A1	67.2	4649365	present
Polynucleobacter necessarius STIR1	45.6	1560469	absent
Polynucleobacter necessarius subsp. asymbioticus QLW-	44.8	2159490	absent
Porphyromonas asaccharolytica DSM	52.5	2186370	absent
Porphyromonas gingivalis ATCC 33277	48.4	2354886	absent
Porphyromonas gingivalis TDC60	48.3	2339898	absent
Porphyromonas	48.3	2343476	absent
Prevotella denticola	50.4	2937589	absent
Prevotella melaninogenica ATCC	40.9	1796408	absent
Prevotella melaninogenica ATCC	41.1	1371874	absent
Prevotella ruminicola	47.7	3619559	absent
Prochlorococcus marinus CCMP1375	36.4	1751080	absent
Prochlorococcus marinus CCMP1378	30.8	1657990	absent
Prochlorococcus marinus MIT9313	50.7	2410873	absent
Prochlorococcus marinus str. AS9601	31.3	1669886	absent
Prochlorococcus marinus str. MIT 9211	38	1688963	absent
Prochlorococcus marinus str. MIT 9215	31.1	1738790	absent
Prochlorococcus marinus str. MIT 9301	31.3	1641879	absent
Prochlorococcus marinus str. MIT 9303	50	2682675	absent
Prochlorococcus marinus str. MIT 9312	31.2	1709204	absent
Prochlorococcus marinus str. MIT 9515	30.8	1704176	absent

Prochlorococcus marinus str. NATL1A	35	1864731	absent
Prochlorococcus marinus str. NATL2A	35.1	1842899	absent
Propionibacterium acnes KPA171202	60	2560265	absent
Propionibacterium acnes SK137	60.1	2495334	absent
Propionibacterium freudenreichii subsp. shermanii CIRM-BIA1	67.3	2616384	absent
Prosthecochloris aestuarii DSM 271	50.1	2512923	present
Prosthecochloris vibrioformis DSM 265	53	1966858	absent
Proteus mirabilis	38.9	4063606	present
Pseudoalteromonas atlantica T6c	44.6	5187005	absent
Pseudoalteromonas haloplanktis TAC125	40.2	3214944	absent
Pseudoalteromonas haloplanktis TAC125 chromosome II	39.4	635328	absent
Pseudoalteromonas sp. SM9913	40.4	3332787	absent
Pseudoalteromonas sp. SM9913 chromosome II	39.8	704884	absent
Pseudomonas	66.6	6264404	absent
Pseudomonas aeruginosa LESB58	66.3	6601757	absent
Pseudomonas	66.4	6588339	absent
Pseudomonas aeruginosa UCBPP-	66.3	6537648	absent
Pseudomonas brassicacearum subsp. brassicacearum	60.8	6843248	absent
Pseudomonas	64.2	5888780	absent
Pseudomonas	63.3	7074893	absent
Pseudomonas fluorescens PfO-1	60.5	6438405	absent
Pseudomonas fluorescens SBW25	60.5	6722539	present
Pseudomonas fulva 12-	63.5	4920769	absent
Pseudomonas mendocina NK-01	62.5	5434353	absent
Pseudomonas	64.7	5072807	absent
Pseudomonas putida F1	61.9	5959964	absent
Pseudomonas putida	61.9	6078430	absent
Pseudomonas putida	61.5	6181863	absent
Pseudomonas putida	61.4	5774330	absent
Pseudomonas stutzeri	63.9	4567418	absent
Pseudomonas syringae	58.4	6397126	absent
Pseudomonas syringae pv. phaseolicola 1448A	58	5928787	present

Pseudomonas syringae pv. syringae B728a	59.2	6093698	absent
Pseudonocardia dioxanivorans CB1190	73.3	7096571	present
Pseudoxanthomonas suwonensis 11-1	70.2	3419049	absent
Psychrobacter arcticum	42.8	2650701	absent
Psychrobacter cryohalolentis K5	42.3	3059876	present
Psychrobacter sp.	44.9	2978976	present
Psychromonas	40.1	4559598	absent
Pusillimonas sp. T7-7	56.9	3883605	present
Rahnella sp. Y9602	52.3	4864217	present
Ralstonia eutropha H16 chromosome 1	66.5	4052032	absent
Ralstonia eutropha H16 chromosome 2	66.8	2912490	absent
Ralstonia eutropha	64.7	3806533	present
Ralstonia eutropha JMP134 chromosome 2	65	2726152	present
Ralstonia metallidurans CH34 chromosome 1	63.8	3928089	present
Ralstonia metallidurans CH34 chromosome 2	63.6	2580084	present
Ralstonia pickettii 12D	63.6	3647724	present
Ralstonia pickettii 12D chromosome 2	64.5	1323321	present
Ralstonia pickettii 12J	63.4	3942557	present
Ralstonia pickettii 12J chromosome 2	64.5	1302238	present
Ralstonia solanacearum CFBP2957	66.5	3417386	absent
Ralstonia solanacearum	66.1	2085000	absent
Ralstonia solanacearum PSI07 chromosome 2	66.4	3520618	absent
Renibacterium salmoninarum ATCC	56.3	3155250	absent
Rhizobium etli CFN 42	61.3	4381608	present
Rhizobium etli CIAT	61.7	4513324	present
Rhizobium leguminosarum bv.	61.1	4767043	present
Rhizobium leguminosarum bv.	61.5	4537948	present
Rhizobium leguminosarum bv.	61.1	5057142	present
Rhizobium sp. NGR234	63	3925702	present
Rhodobacter capsulatus SB1003	66.6	3738958	present
Rhodobacter	69	3188609	present
Rhodobacter sphaeroides 2.4.1	69	943016	present
Rhodobacter sphaeroides ATCC	68.5	3217726	present

Rhodobacter sphaeroides ATCC	69.1	3147721	present
Rhodobacter sphaeroides ATCC	68.6	1219053	present
Rhodobacter sphaeroides KD131	69.2	3152792	present
Rhodobacter sphaeroides KD131	68.7	1297647	present
Rhodococcus	62.3	6516310	present
Rhodococcus jostii	67.5	7804765	present
Rhodococcus opacus	67.9	7913450	absent
Rhodoferax	59.9	4712337	present
Rhodomicrobium equis	68.8	5043170	absent
Rhodomicrobium vannielii ATCC 17100	62.2	4014469	absent
Rhodopseudomonas palustris BisA53	64.4	5505494	absent
Rhodopseudomonas palustris BisB18	65	5513844	absent
Rhodopseudomonas palustris BisB5	64.8	4892717	absent
Rhodopseudomonas palustris CGA009	65	5459213	absent
Rhodopseudomonas palustris DX-1	65.4	5404117	absent
Rhodopseudomonas palustris HaA2	66	5331656	absent
Rhodopseudomonas palustris TIE-1	64.9	5744041	absent
Rhodospirillum	70.5	4355548	absent
Rhodospirillum rubrum ATCC11170	65.4	4352825	present
Rhodothermus marinus DSM 4252	64.5	3261604	present
Rickettsia africae ESF-5	32.4	1278540	present
Rickettsia akari str.	32.3	1231060	absent
Rickettsia bellii OSU	31.6	1528980	absent
Rickettsia bellii	31.6	1522076	absent
Rickettsia canadensis str. McKiel	31.1	1159772	absent
Rickettsia conorii	32.4	1268755	absent
Rickettsia felis	32.5	1485148	present
Rickettsia massiliae	32.5	1360898	present
Rickettsia peacockii str.	32.6	1288492	present
Rickettsia prowazekii strain Madrid E	29	1111523	absent
Rickettsia rickettsii str.	32.4	1268175	absent
Rickettsia rickettsii str. Sheila Smith	32.5	1257710	absent
Rickettsia typhi	28.9	1111496	absent
Riemerella anatipestifer DSM 15868	35	2155121	absent
Robiginitalea biformata HTCC2501	55.3	3530383	absent

Roseiflexus castenholzii DSM 13941	60.7	5723298	absent
Roseiflexus sp. RS-1	60.4	5801598	absent
Roseobacter denitrificans OCh 114	59	4133097	absent
Rothia dentocariosa ATCC 17931	53.7	2506025	absent
Rothia mucilaginosa	59.6	2264603	absent
Rubrobacter xylanophilus DSM	70.5	3225748	absent
Ruegeria pomeroyi	64.2	4109442	present
Ruminococcus albus 7	44.2	3685408	present
Saccharomonospora viridis DSM 43017	67.3	4308349	absent
Saccharophagus degradans 2-40	45.8	5057531	absent
Saccharopolyspora erythraea NRRL 2338	71.1	8212805	absent
Salinibacter ruber	66.1	3619447	absent
Salinibacter ruber DSM	66.2	3551823	present
Salinispora arenicola	69.5	5786361	absent
Salinispora tropica	69.5	5183331	absent
Salmonella enterica subsp. arizonae serovar	51.4	4600800	absent
Salmonella enterica subsp. enterica serovar Agona str. SL483	52.1	4798660	present
Salmonella enterica subsp. enterica serovar Choleraesuis str. SC-	52.2	4755700	present
Salmonella enterica subsp. enterica serovar Dublin str.	52.2	4842908	present
Salmonella enterica subsp. enterica serovar Enteritidis str. P125109	52.2	4685848	absent
Salmonella enterica subsp. enterica serovar Gallinarum str. 287/91	52.2	4658697	absent
Salmonella enterica subsp. enterica serovar Heidelberg str. SL476	52.1	4888768	present
Salmonella enterica subsp. enterica serovar Newport str. SL254	52.2	4827641	present
Salmonella enterica subsp. enterica serovar Paratyphi A str.	52.2	4581797	absent
Salmonella enterica subsp. enterica serovar Paratyphi A str. ATCC	52.2	4585229	absent
Salmonella enterica subsp. enterica serovar Paratyphi B str. SPB7	52.1	4858887	absent

Salmonella enterica subsp. enterica serovar Paratyphi C strain	52.2	4833080	present
Salmonella enterica subsp. enterica serovar Schwarzengrund str. CVM19633	52.2	4709075	present
Salmonella enterica subsp. enterica serovar	52.1	4809037	present
Salmonella enterica subsp. enterica serovar	52.1	4791961	absent
Salmonella enterica subsp. enterica serovar Typhimurium LT2	52.2	4857432	present
Sanguibacter keddieii DSM 10542	71.9	4253413	absent
Sebaldella termitidis ATCC 33386	33.5	4418842	present
Segniliparus rotundus DSM 44985	66.8	3157527	absent
Selenomonas sputigena ATCC 35185	57.1	2568361	absent
Serratia proteamaculans	55.1	5448853	present
Serratia sp. AS12	56	5443009	absent
Serratia sp. AS9	56	5442880	absent
Shewanella	53.6	4306142	absent
Shewanella baltica	46.3	5127376	present
Shewanella baltica	46.3	5229686	present
Shewanella baltica	46.3	5347283	present
Shewanella baltica	46.3	5145902	present
Shewanella denitrificans OS217	45.1	4545906	absent
Shewanella frigidimarina NCIMB	41.6	4845257	absent
Shewanella halifaxensis HAW-EB4	44.6	5226917	absent
Shewanella loihica PV-	53.7	4602594	absent
Shewanella oneidensis	46	4969803	present
Shewanella pealeana ATCC 700345	44.7	5174581	absent
Shewanella	43.3	5396476	absent
Shewanella putrefaciens CN-32	44.5	4659220	absent
Shewanella sediminis HAW-EB3	46.1	5517674	absent
Shewanella sp. ANA-3	48.1	4972204	present
Shewanella sp. MR-4	47.9	4706287	absent
Shewanella sp. MR-7	47.9	4792610	present
Shewanella sp. W3-18-	44.6	4708380	absent
Shewanella violacea	44.7	4962103	absent
Shewanella woodyi ATCC 51908	43.7	5935403	absent
Shigella boydii CDC	51.3	4615997	present
Shigella boydii Sb227	51.2	4519823	present

Shigella dysenteriae	51.2	4369232	present
Shigella flexneri 2a str	50.9	4607203	present
Shigella flexneri 2a str.	50.9	4599354	absent
Shigella flexneri 5 str.	50.9	4574284	absent
Shigella sonnei Ss046	51	4825265	present
Sideroxydans lithotrophicus ES-1	57.5	3003656	absent
Silicibacter sp. TM1040	60.4	3200938	present
Sinorhizobium medicae WSM419	61.5	3781904	present
Sinorhizobium meliloti	62.7	3654135	present
Sinorhizobium meliloti	62.7	3820344	present
Sinorhizobium meliloti AK83 chromosome 2	62.4	1680879	present
Sinorhizobium meliloti AK83 chromosome 3	60	1312480	present
Slackia heliotrinireducens DSM	60.2	3165038	absent
Sodalis glossinidius str. morsitans	54.7	4171146	present
Solibacter usitatus	61.9	9965640	absent
Sorangium cellulosum 'So ce 56'	71.4	13033779	absent
Sphaerobacter thermophilus DSM	68.1	2741033	absent
Sphaerobacter thermophilus DSM	68.1	1252731	absent
Sphingobacterium sp.	41.1	6226409	absent
Sphingobium chlorophenolicum L-1	63.9	3080818	present
Sphingobium chlorophenolicum L-1	63.6	1368670	present
Sphingobium	64.8	3514822	present
Sphingobium japonicum UT26S	65.9	681892	present
Sphingomonas wittichii	68.4	5382261	present
Sphingopyxis alaskensis RB2256	65.5	3345170	present
Spirochaeta coccoides DSM 17374	50.6	2227296	absent
Spirochaeta smaragdinae DSM	49	4653970	absent
Spirochaeta sp. Buddy	48.9	3316466	absent
Spirochaeta thermophila DSM 6192	61.9	2472645	absent
Spirosoma linguale	50.2	8078757	present
Stackebrandtia nassauensis DSM	68.1	6841557	absent
Staphylococcus aureus	32.8	2742531	absent
Staphylococcus aureus strain Mu50	32.9	2878529	present
Staphylococcus aureus subsp. aureus COL	32.8	2809422	present

Staphylococcus aureus subsp. aureus ED98	32.8	2824404	present
Staphylococcus aureus subsp. aureus JH1	33	2906507	present
Staphylococcus aureus subsp. aureus JH9	32.9	2906700	present
Staphylococcus aureus subsp. aureus	32.8	2902619	absent
Staphylococcus aureus subsp. aureus	32.9	2799802	absent
Staphylococcus aureus subsp. aureus Mu3	32.9	2880168	absent
Staphylococcus aureus subsp. aureus MW2	32.8	2820462	absent
Staphylococcus aureus subsp. aureus N315	32.8	2814816	present
Staphylococcus aureus subsp. aureus NCTC	32.9	2821361	absent
Staphylococcus aureus subsp. aureus str.	32.9	2878897	absent
Staphylococcus aureus subsp. aureus	32.8	2872769	present
Staphylococcus aureus subsp. aureus	32.8	2872915	present
Staphylococcus carnosus subsp.	34.6	2566424	absent
Staphylococcus epidermidis	32.1	2499279	absent
Staphylococcus epidermidis RP62A	32.2	2616530	present
Staphylococcus haemolyticus	32.8	2685015	absent
Staphylococcus lugdunensis HKU09-01	33.9	2658366	absent
Staphylococcus pseudintermedius	37.5	2617381	absent
Staphylococcus saprophyticus subsp.	33.2	2516575	present
Starkeya novella DSM	67.9	4765023	absent
Stenotrophomonas maltophilia K279a	66.3	4851126	absent
Stenotrophomonas maltophilia R551-3	66.3	4573969	absent
Stigmatella aurantiaca DW4/3-1	67.5	10260756	absent
Streptobacillus moniliformis DSM	26.3	1662578	present
Streptococcus agalactiae 2603V/R	35.6	2160267	absent
Streptococcus	35.6	2127839	absent
Streptococcus agalactiae NEM316	35.6	2211485	absent

Streptococcus dysgalactiae subsp. equisimilis GGS 124	39.6	2106340	absent
Streptococcus equi subsp. equi 4047	41.3	2253793	absent
Streptococcus equi subsp. zooepidemicus	41.5	2149868	absent
Streptococcus equi subsp. zooepidemicus str. MGCS10565	41.8	2024171	absent
Streptococcus gallolyticus subsp. gallolyticus ATCC	37.6	2356444	present
Streptococcus gallolyticus UCN34	37.6	2350911	absent
Streptococcus gordonii str. Challis substr. CH1	40.5	2196662	absent
Streptococcus mitis B6	40	2146611	absent
Streptococcus mutans	36.8	2013587	absent
Streptococcus mutans	36.8	2030921	absent
Streptococcus oralis	41.1	1958690	absent
Streptococcus parauberis KCTC	35.5	2143887	absent
Streptococcus pasteurianus ATCC	37.4	2100077	absent
Streptococcus pneumoniae 670-6B	39.6	2240045	absent
Streptococcus pneumoniae 70585	39.7	2184682	absent
Streptococcus pneumoniae AP200	39.5	2130580	absent
Streptococcus pneumoniae ATCC	39.5	2221315	absent
Streptococcus pneumoniae CGSP14	39.5	2209198	absent
Streptococcus pneumoniae D39	39.7	2046115	absent
Streptococcus	39.7	2078953	absent
Streptococcus pneumoniae	39.6	2245615	absent
Streptococcus	39.7	2120234	absent
Streptococcus pneumoniae P1031	39.7	2111882	absent
Streptococcus	39.7	2038615	absent
Streptococcus pneumoniae	39.8	2112148	absent
Streptococcus pneumoniae	39.8	2088772	absent
Streptococcus pneumoniae TIGR4	39.7	2160842	absent
Streptococcus pyogenes M1 GAS	38.5	1852441	absent
Streptococcus pyogenes MGAS10270	38.4	1928252	absent

Streptococcus pyogenes MGAS10394	38.7	1899877	absent
Streptococcus pyogenes MGAS10750	38.3	1937111	absent
Streptococcus pyogenes MGAS2096	38.7	1860355	absent
Streptococcus pyogenes MGAS315	38.6	1900521	absent
Streptococcus pyogenes MGAS5005	38.5	1838554	absent
Streptococcus pyogenes MGAS6180	38.4	1897573	absent
Streptococcus pyogenes MGAS9429	38.5	1836467	absent
Streptococcus pyogenes NZ131	38.6	1815785	absent
Streptococcus pyogenes	38.6	1894275	absent
Streptococcus pyogenes str. Manfredo	38.6	1841271	absent
Streptococcus pyogenes strain MGAS8232	38.5	1895017	absent
Streptococcus sanguinis	43.4	2388435	absent
Streptococcus suis	41.1	2096309	absent
Streptococcus suis	41.1	2095698	absent
Streptococcus suis	41.1	2146229	present
Streptococcus suis P1/7	41.3	2007491	absent
Streptococcus suis	41.1	2095898	absent
Streptococcus suis ST3	41.3	2028815	absent
Streptococcus thermophilus	39.1	1796226	absent
Streptococcus thermophilus LMD-9	39.1	1856368	present
Streptococcus thermophilus LMG	39.1	1796846	absent
Streptococcus uberis	36.6	1852352	absent
Streptomyces avermitilis MA-4680	70.7	9025608	present
Streptomyces coelicolor	72.1	8667507	present
Streptomyces griseus subsp. griseus NBRC	72.2	8545929	absent
Streptomyces scabiei	71.5	10148695	absent
Streptosporangium roseum DSM 43021	70.9	10341314	present
Sulfuricurvum kujiense DSM 16994	45	2574824	present
Sulfurihydrogenibium azorense Az-Fu1	32.8	1640877	absent
Sulfurihydrogenibium sp. YO3AOP1	32	1838442	absent
Sulfurimonas autotrophica DSM	35.2	2153198	absent
Sulfurospirillum deleyianum DSM 6946	39	2306351	absent
Sulfurovum sp. NBC37-	43.9	2562277	absent

Symbiobacterium thermophilum IAM	68.7	3566135	absent
Synechococcus elongatus PCC 6301	55.5	2696255	absent
Synechococcus elongatus PCC 7942	55.5	2695903	present
Synechococcus sp.	52.4	2606748	absent
Synechococcus sp.	59.2	2510659	absent
Synechococcus sp.	54.2	2234828	absent
Synechococcus sp. JA-2-3B'a(2-13)	58.5	3046682	absent
Synechococcus sp. JA-	60.2	2932766	absent
Synechococcus sp. PCC	49.6	3008047	present
Synechococcus sp.	60.8	2224914	absent
Synechococcus sp. WH	60.2	2366980	absent
Synechococcus sp.	59.4	2434428	absent
Synechocystis sp. PCC	47.7	3573470	absent
Syntrophobacter fumaroxidans MPOB	59.9	4990251	absent
Syntrophobotulus glycolicus DSM 8271	46.4	3406739	absent
Syntrophomonas wolfei subsp. wolfei str.	44.9	2936195	absent
Syntrophothermus lipocalidus DSM 12680	51	2405559	absent
Syntrophus	51.5	3179300	absent
Taylorella equigenitalis	37.4	1695860	absent
Tepidanaerobacter sp.	37.5	2759867	absent
Teredinibacter turnerae	50.9	5193164	absent
Terriglobus saanensis	57.3	5095226	absent
Thauera sp. MZ1T	68.4	4496212	present
Thermaerobacter marianensis DSM	72.5	2844696	absent
Thermanaerovibrio acidaminovorans DSM	63.8	1848474	absent
Thermincola sp. JR	45.9	3157416	absent
Thermoanaerobacter brockii subsp. finnii	34.5	2344824	absent
Thermoanaerobacter italicus Ab9	34.1	2451061	absent
Thermoanaerobacter mathranii subsp.	34.3	2306092	absent
Thermoanaerobacter pseudethanolicus	34.5	2362816	absent
Thermoanaerobacter sp.	34.5	2456520	absent
Thermoanaerobacter sp.	34.5	2457259	absent
Thermoanaerobacter tengcongensis strain	37.6	2689445	absent
Thermoanaerobacterium thermosaccharolyticum	34.1	2785752	absent
Thermoanaerobacterium xylanolyticum LX-11	35	2534358	absent

Thermobaculum terrenum ATCC BAA-	48.1	2026947	absent
Thermobaculum terrenum ATCC BAA-798 chromosome 2	63.8	1074634	absent
Thermobifida fusca YX	67.5	3642249	absent
Thermobispora bispora DSM 43833	72.4	4189976	absent
Thermocrinis albus DSM 14484	46.9	1500577	absent
Thermodesulfobium narugense DSM 14796	33.9	1898865	absent
Thermodesulfovibrio yellowstonii DSM	34.1	2003803	absent
Thermomicrobium roseum DSM 5159	63.6	2003006	present
Thermomonospora curvata DSM 43183	71.6	5639016	absent
Thermosediminibacter oceani DSM 16646	46.8	2280035	absent
Thermosipho africanus TCF52B	30.8	2016657	absent
Thermosipho melanesiensis BI429	31.4	1915238	absent
Thermosynechococcus elongatus BP-1	53.9	2593857	absent
Thermotoga lettingae	38.7	2135342	absent
Thermotoga maritima	46.2	1860725	absent
Thermotoga naphthophila RKU-10	46.1	1809823	absent
Thermotoga neapolitana DSM 4359	46.9	1884562	absent
Thermotoga petrophila	46.1	1823511	absent
Thermotoga sp. RQ2	46.2	1877693	absent
Thermovibrio ammonificans HB-1	52.1	1682965	present
Thermus scotoductus	64.9	2346803	present
Thermus thermophilus	69.4	1894877	present
Thermus thermophilus	69.5	1849742	present
Thioalkalimicrobium cyclicum ALM1	47	1932455	absent
Thioalkalivibrio sp. HL-	65.1	3464554	absent
Thioalkalivibrio sp.	65.9	2744800	absent
Thiobacillus denitrificans ATCC	66.1	2909809	absent
Thiomicrospira crunogena XCL-2	43.1	2427734	absent
Thiomicrospira denitrificans ATCC	34.5	2201561	absent
Thiomonas intermedia	63.9	3396378	present
Tolumonas auensis	49	3471292	absent
Treponema azotonutricium ZAS-9	49.8	3855671	absent

Treponema brennaborense DSM	51.5	3055580	absent
Treponema denticola ATCC 35405	37.9	2843201	absent
Treponema pallidum	52.8	1138011	absent
Treponema pallidum subsp. pallidum SS14	52.8	1139457	absent
Treponema primitia	50.8	4059867	absent
Treponema succinifaciens DSM	39.2	2731853	present
Trichodesmium erythraeum IMS101	34.1	7750108	absent
Tropheryma whipplei	46.3	925938	absent
Tropheryma whipplei	46.3	927303	absent
Truepera radiovictrix DSM 17093	68.1	3260398	absent
Tsukamurella paurometabola DSM	68.4	4379918	present
Ureaplasma parvum serovar 3 str. ATCC	25.5	751679	absent
Ureaplasma urealyticum	25.5	751719	absent
Ureaplasma urealyticum serovar 10 str. ATCC	25.8	874478	absent
Variovorax paradoxus	66.5	6550056	absent
Variovorax paradoxus	67.6	5626353	absent
Variovorax paradoxus S110 chromosome 2	67	1128644	absent
Veillonella parvula	38.6	2132142	absent
Verminephrobacter eiseniae EF01-2	65.3	5566749	present
Verrucosispora maris AB-18-032	70.9	6673976	present
Vibrio anguillarum 775	44.6	3063912	absent
Vibrio anguillarum 775 chromosome II	44.1	988135	absent
Vibrio cholerae	47.7	2961149	absent
Vibrio cholerae	46.9	1072315	absent
Vibrio cholerae M66-2	47.8	2892523	absent
Vibrio cholerae M66-2 chromosome II	47	1046382	absent
Vibrio cholerae MJ-	47.5	3149584	absent
Vibrio cholerae MJ-1236 chromosome 2	46.7	1086784	absent
Vibrio cholerae O395 chromosome 1	46.9	1108250	absent
Vibrio cholerae O395 chromosome 2	47.8	3024069	absent
Vibrio fischeri ES114	39	2897536	absent
Vibrio fischeri ES114 chromosome II	37	1330333	absent
Vibrio fischeri MJ11	38.9	2905029	present
Vibrio fischeri MJ11 chromosome II	37.2	1418848	present

Vibrio harveyi ATCC BAA-1116	45.5	3765351	present
Vibrio harveyi ATCC BAA-1116 chromosome	45.3	2204018	present
Vibrio parahaemolyticus	45.4	3288558	absent
Vibrio parahaemolyticus	45.4	1877212	absent
Vibrio sp. Ex25	44.9	3259580	absent
Vibrio sp. Ex25	44.9	1829445	absent
Vibrio splendidus	44	3299302	absent
Vibrio splendidus LGP32 chromosome 2	43.6	1675519	absent
Vibrio vulnificus	46.4	3281944	absent
Vibrio vulnificus CMCP6 chromosome II	47.1	1844853	absent
Vibrio vulnificus MO6-	46.7	3194232	absent
Vibrio vulnificus MO6-24/O chromosome II	47.4	1813536	absent
Vibrio vulnificus YJ016	46.4	3354505	present
Vibrio vulnificus YJ016 chromosome II	47.2	1857073	present
Waddlia chondrophila WSU 86-1044	43.8	2116312	present
Weeksella virosa DSM	35.9	2272954	absent
Wigglesworthia	22.5	697724	absent
Wolbachia endosymbiont of Culex	34.2	1482455	absent
Wolbachia endosymbiont of	35.2	1267782	absent
Wolbachia endosymbiont strain	34.2	1080084	absent
Wolbachia sp. wRi	35.2	1445873	absent
Wolinella succinogenes	48.5	2110355	absent
Xanthobacter autotrophicus Py2	67.5	5308934	present
Xanthomonas	63	3768695	absent
Xanthomonas axonopodis pv. citri str.	64.8	5175554	absent
Xanthomonas campestris pv.	65	5079002	absent
Xanthomonas campestris pv.	65	5148708	absent
Xanthomonas campestris pv.	65.1	5076188	absent
Xanthomonas campestris pv.	64.7	5178466	present
Xanthomonas oryzae pv. oryzae KACC10331	63.7	4941439	absent
Xanthomonas oryzae pv. oryzae MAFF	63.7	4940217	absent
Xanthomonas oryzae pv. oryzae PXO99A	63.6	5240075	absent
Xenorhabdus bovienii	45	4225498	absent

Xenorhabdus nematophila ATCC	44.2	4432590	present
Xylanimonas cellulosilytica DSM	72.5	3742776	present
Xylella fastidiosa	51.8	2519802	present
Xylella fastidiosa 9a5c	52.7	2679306	present
Xylella fastidiosa M12	51.9	2475130	absent
Xylella fastidiosa M23	51.8	2535690	present
Yersinia enterocolitica subsp. enterocolitica	47.3	4615899	present
Yersinia enterocolitica subsp. palearctica	47	4552107	present
Yersinia pestis Angola	47.6	4504254	present
Yersinia pestis Antiqua	47.7	4702289	present
Yersinia pestis biovar Microtus str. 91001	47.7	4595065	present
Yersinia pestis KIM	47.6	4600755	absent
Yersinia pestis	47.6	4534590	present
Yersinia pestis	47.6	4517345	present
Yersinia pestis strain	47.6	4653728	present
Yersinia pestis Z176003	47.7	4553586	present
Yersinia pseudotuberculosis IP	47.5	4723306	present
Yersinia pseudotuberculosis IP	47.6	4744671	present
Yersinia pseudotuberculosis	47.5	4695619	present
Yersinia pseudotuberculosis	47.5	4689441	absent
Zunongwangia profunda SM-A87	36.2	5128187	absent
Zymomonas mobilis subsp. mobilis NCIB	46.8	2124771	present
Zymomonas mobilis subsp. mobilis ZM4	46.3	2056416	absent
