# Supplementary material for: Comparative Analyses of Base Compositions, DNA Sizes, and Dinucleotide Frequency Profiles in Archaeal and Bacterial Chromosomes and Plasmids
Source: Int J Evol Biol. 2012 Mar 26;2012:342482. doi: 10.1155/2012/342482 (PMC3321278; doi:10.1155/2012/342482)
Supplement: Supplementary file 4 [file 342482.f4.pdf]

Supplementary Table S4. Archaeal chromosomes compared in this analysis.

Organism	GC content (%)	Chromosome size (bp)	Plasmid
Acidianus hospitalis W1	34.1	2137654	absent
Acidilobus saccharovorans 345-15	57.2	1496453	absent
Aciduliprofundum	39.2	1486778	absent
Aeropyrum pernix	56.3	1669696	absent
Archaeoglobus fulgidus	48.6	2178400	absent
Archaeoglobus profundus DSM 5631	42	1560622	presnet
Archaeoglobus	47	1901943	absent
Caldivirga	43.1	2077567	absent
Candidatus Korarchaeum	49	1590757	absent
Candidatus Methanoregula boonei	54.5	2542943	absent
Candidatus Methanosphaerula	55.4	2922917	absent
Cenarchaeum	57.4	2045086	absent
Desulfurococcus kamchatkensis 1221n	45.3	1365223	absent
Desulfurococcus mucosus DSM 2162	53.1	1314639	absent
Ferroglobus placidus	44.1	2196266	absent
Halalkalicoccus jeotgali	65	2809118	presnet
Haloarcula marismortui ATCC 43049	62.4	3131724	presnet
Haloarcula marismortui ATCC 43049	57.2	288050	present
Halobacterium	68	2000962	present
Halobacterium sp.	67.9	2014239	present
Haloferax volcanii DS2	66.6	2847757	present
Halogeometricum borinquense DSM	61.1	2820544	present
Halomicrobium mukohataei DSM 12286	65.6	3110487	present
Halopiger xanaduensis	66	3668009	present
Haloquadratum walsbyi DSM 16790	47.9	3132494	present
Halorhabdus utahensis DSM 12940	62.9	3116795	absent
Halorubrum lacusprofundi ATCC	66.7	2735295	present
Halorubrum lacusprofundi ATCC	57.1	525943	present
Haloterrigena turkmenica DSM 5511	65.8	3889038	present
Hyperthermus butylicus DSM 5456	53.7	1667163	absent
Ignicoccus hospitalis	56.5	1297538	absent
Ignisphaera aggregans DSM 17230	35.7	1875953	absent
Metallosphaera cuprina	42	1840348	absent

Metallosphaera sedula DSM 5348	46.2	2191517	absent
Methanobacterium sp.	35.8	2583753	absent
Methanobacterium sp.	35.7	2546541	absent
Methanobrevibacter ruminantium M1	32.6	2937203	absent
Methanobrevibacter smithii ATCC 35061	31	1853160	absent
Methanocaldococcus fervens AG86	32.2	1485061	present
Methanocaldococcus infernus ME	33.6	1328194	absent
Methanocaldococcus sp. FS406-22	32	1760939	present
Methanocaldococcus vulcanius M7	31.5	1746329	present
Methanocella paludicola	54.9	2957635	absent
Methanococcoides burtonii DSM 6242	40.8	2575032	absent
Methanococcus aeolicus Nankai-3	30	1569500	absent
Methanococcus	31.4	1664970	present
Methanococcus maripaludis (strain S2)	33.1	1661137	abesnt
Methanococcus	33	1780761	present
Methanococcus	33.4	1744193	absent
Methanococcus	33.3	1772694	absent
Methanococcus	31.3	1720048	absent
Methanococcus voltae	28.6	1936387	absent
Methanocorpusculum labreanum Z	50	1804962	absent
Methanoculleus	62.1	2478101	absent
Methanohalobium evestigatum Z-7303	36.6	2242317	present
Methanohalophilus mahii DSM 5219	42.6	2012424	absent
Methanoplanus petrolearius DSM 11571	47.4	2843290	absent
Methanopyrus kandleri	61.2	1694969	absent
Methanosaeta concilii	51	3008626	present
Methanosaeta	53.5	1879471	absent
Methanosarcina acetivorans str. C2A	42.7	5751492	absent
Methanosarcina barkeri str. fusaro	39.3	4837408	present
Methanosarcina mazei strain Goe1	41.5	4096345	absent
Methanosphaera stadtmanae DSM 3091	27.6	1767403	absent
Methanospirillum	45.1	3544738	absent
Methanothermobacter marburgensis str.	48.6	1634695	present
Methanothermobacter thermoautotrophicus str.	49.5	1751377	absent

Methanothermococcus okinawensis IH1	29.3	1662525	present
Methanothermus fervidus DSM 2088	31.6	1243342	absent
Methanotorris igneus	32.3	1854197	absent
Nanoarchaeum equitans	31.6	490885	absent
Natrialba magadii	61.4	3751858	presnet
Natronomonas pharaonis DSM 2160	63.4	2595221	present
Nitrosopumilus	34.2	1645259	absent
Picrophilus torridus	36	1545895	absent
Pyrobaculum	51.4	2222430	absent
Pyrobaculum arsenaticum DSM	55.1	2121076	absent
Pyrobaculum calidifontis JCM 11548	57.2	2009313	absent
Pyrobaculum islandicum DSM 4184	49.6	1826402	absent
Pyrococcus abyssi	44.7	1765118	absent
Pyrococcus furiosus	40.8	1908256	absent
Pyrococcus horikoshii	41.9	1738505	absent
Pyrococcus sp. NA2	42.7	1861320	absent
Staphylothermus hellenicus DSM 12710	36.8	1580347	absent
Staphylothermus	35.7	1570485	absent
Sulfolobus acidocaldarius DSM	36.7	2225959	absent
Sulfolobus islandicus	35.3	2722032	present
Sulfolobus islandicus	35.1	2736272	absent
Sulfolobus islandicus	35.1	2608832	absent
Sulfolobus islandicus	35	2692402	absent
Sulfolobus islandicus	35	2586647	absent
Sulfolobus islandicus	35.4	2702058	absent
Sulfolobus islandicus	35.3	2812165	present
Sulfolobus solfataricus	35.8	2992245	absent
Sulfolobus tokodaii	32.8	2694756	absent
Thermococcus	41.8	2010078	absent
Thermococcus gammatolerans EJ3	53.6	2045438	absent
Thermococcus kodakaraensis KOD1	52	2088737	absent
Thermococcus	51.3	1847607	absent
Thermococcus sibiricus	40.2	1845800	absent
Thermofilum pendens	57.7	1781889	absent
Thermoplasma	46	1564906	absent
Thermoplasma	39.9	1584804	absent
Thermoproteus neutrophilus V24Sta	59.9	1769823	absent
Thermoproteus	59.7	1936063	absent
Thermosphaera aggregans DSM 11486	46.7	1316595	absent
Uncultured methanogenic archaeon	54.6	3179916	absent

Vulcanisaeta distributa DSM 14429	45.4	2374137	absent
Vulcanisaeta moutnovskia 768-28	42.4	2298983	absent
